# The development of a novel SNP genotyping assay to differentiate cacao clones

**DOI:** 10.1038/s41598-019-45884-8

**Published:** 2019-07-02

**Authors:** Jocelyn De Wever, Helena Everaert, Frauke Coppieters, Hayley Rottiers, Koen Dewettinck, Steve Lefever, Kathy Messens

**Affiliations:** 1 Research unit Molecular Biology, Department of Biotechnology, Faculty of Bioscience Engineering, Ghent University, Ghent, 9000 Belgium; 2 Center for Medical Genetics Ghent (CMGG), Ghent University Hospital, Ghent, 9000 Belgium; 3 Laboratory of Food Technology and Engineering (FTE), Department of Food Technology, Safety and Health, Faculty of Bioscience Engineering, Ghent University, Ghent, 9000 Belgium; 4 Cancer Research Institute Ghent (CRIG), Ghent University, Ghent, 9000 Belgium; 5 Bioinformatics Institute Ghent (BIG), Ghent University, Ghent, 9000 Belgium

**Keywords:** Genetic markers, Plant biotechnology, Plant genetics

## Abstract

In this study, a double-mismatch allele-specific (DMAS) qPCR SNP genotyping method has been designed, tested and validated specifically for cacao, using 65 well annotated international cacao reference accessions retrieved from the Center for Forestry Research and Technology Transfer (CEFORTT) and the International Cocoa Quarantine Centre (ICQC). In total, 42 DMAS-qPCR SNP genotyping assays have been validated, with a 98.05% overall efficiency in calling the correct genotype. In addition, the test allowed for the identification of 15.38% off-types and two duplicates, highlighting the problem of mislabeling in cacao collections and the need for conclusive genotyping assays. The developed method showed on average a high genetic diversity (H_e_ = 0.416) and information index (I = 0.601), making it applicable to assess intra-population variation. Furthermore, only the 13 most informative markers were needed to achieve maximum differentiation. This simple, effective method provides robust and accurate genotypic data which allows for more efficient resource management (e.g. tackling mislabeling, conserving valuable genetic material, parentage analysis, genetic diversity studies), thus contributing to an increased knowledge on the genetic background of cacao worldwide. Notably, the described method can easily be integrated in other laboratories for a wide range of objectives and organisms.

## Introduction

The beans of *Theobroma cacao* L. (*T. cacao*) (2n = 20) are worldwide acknowledged for their application in the chocolate industry^1^. They are harvested in the tropical regions between 10 to 20 degrees north and south of the equator. Although the South-American Amazon region is recognized as the center of its origin, the three main cacao producing countries are Ivory Coast, Ghana and Ecuador^2,3^. The flavor characteristics of cacao are mainly influenced by genotype and origin, although growth conditions of the cacao tree and post-harvest processing factors have also shown to be of importance^4,5^. *T. cacao* consists of numerous morphologically distinct populations, mainly subdivided in *Criollo, Trinitario* and *Forastero*. They can all be crossed reciprocally, which is valuable for breeding and propagation. Such crop improvement programs primarily focus on cultivation of new, resistant and high-yielding cacao varieties using characterized genetic resources (germplasm)^6,7^. So far, up to 15 to 44% of the cacao accessions have been estimated to be mislabeled – called off types – as they show different genetic profiles than expected^8–10^. Therefore, more straightforward identification techniques based on genetic markers, also known as DNA fingerprinting methods, have been used.

Simple sequence repeats (SSRs) and single nucleotide polymorphisms (SNPs) are mainly used for genotyping and classifying cacao varieties. Both have been employed successfully in cacao for genetic diversity studies, detecting mislabeling and establishing the genetic relationships within and between populations or individuals. Moreover, they proved to facilitate cacao conservation and domestication, and conjointly assisted in mapping useful genes and selecting breeding parents. In short, the use of genetic markers resulted in a reduction of inbreeding, mislabeling, unwanted progeny, and genetic drift, playing a main role in long-term breeding gains by enhancing efficient and substantiated cacao management^9,11–15^.

SSRs are the most commonly used type of marker for genotyping plants and animals. These tandem nucleotide repeats are highly polymorphic, abundantly present in the genome, co-dominant and suitable for automatization. Their efficiency in cacao genotyping has been demonstrated successfully for multiple applications such as off-type detection^16,17^, progeny improvement^16^, parentage analysis^18^ and diversity assessments^19,20^. Since 2004, a set of fifteen SSRs has been recognized as the international standard for genotyping cacao^21^. In 2008, Motamayor *et al*. used these markers to classify Amazonian cacao germplasm (living genetic resources) more accurately into 10 major genetic clusters, namely *Amelonado, Contamana, Criollo, Curaray, Guiana, Iquitos, Marañon, Nacional, Nanay* and *Purús*^8^. This technique, in combination with the proposed classification, allows to reflect on the genetic diversity of cacao varieties worldwide. Unfortunately, many disadvantages are inherently linked to SSR. Besides the lack of associations with genes and traits^22^, this electrophoresis-based analysis often delivers ambiguous results making inter-lab comparisons difficult. Moreover, the technique is costly and requires specialized training and instrumentation, both usually limited in cacao producing countries and therefore often outsourced and unaffordable for small scale studies^2,8,12,13^.

Alternative molecular markers such as SNPs have been proposed. They constitute the largest class of polymorphisms observed in plant genomes and can be found in coding regions, making associations with traits feasible^23,24^. Approximately 1,560 SNP candidates from a wide range of cacao organs have already been published in Tropgenedb^25^. Recently, Livingstone *et al*. (2015) identified 330,000 SNPs by mapping RNA-seq data from 16 diverse cacao cultivars to the Matina 1–6 genome^26^. Despite the fact that SNPs are biallelic in nature and hence less polymorphic and informative than SSRs, they have shown to be 98% as efficient in calling off-types^27^. Furthermore, SNP-based genotyping is more robust and less ambiguous as it requires only a (quantitative) PCR instrument facilitating data publication in databases accessible worldwide, such as the International Cocoa Germplasm Database (ICGD).

Currently, Maldi-TOF mass spectrometry (MS)^6^ and TaqMan-based quantitative PCR (qPCR) are the golden standard for SNP detection in cacao studies^23,28^. TaqMan-based genotyping has already been applied on-field and both methods can be used in multiplex and automated high-throughput contexts^12^. However, Maldi-TOF MS needs specialized equipment and training, whereas TaqMan-based qPCR assays depend on fluorescently labeled probes. This increases design complexity and cost in comparison to SYBR-green-based qPCR – especially for low throughput genotyping assays. Recently, a cost-effective qPCR-based method has been proposed for SNP genotyping purposes, coined DMAS qPCR. The method is based on straightforward readout of DNA-binding dye based qPCR technology. It entails two parallel qPCR reactions, each including a different allele-specific (AS) primer harboring an artificial mismatch to increase robustness and discriminating power. The advantage of DMAS-qPCR is its ease of use, high analytical sensitivity and specificity (matching TaqMan-based methods) without the need for expensive fluorescently labeled probes^29^. Moreover, a similar SNP genotyping method, known as AS Primer (ASP) PCR, was published in 2010 with promising results in genotyping rice^30^.

This paper describes the development of successful DMAS-qPCR SNP genotyping assays that are useable for cacao mislabeling and genetic diversity studies. Two main objectives can be distinguished, namely (1) the establishment of cacao specific DMAS-qPCR SNP assays, and (2) the testing and validation of resulting DMAS-qPCR assays on a subset of international cacao reference accessions. In general, the applicability, reliability and fast multi-sample and multi-locus SNP genotyping capacity of DMAS-qPCR will be demonstrated.

## Results

### Genotyping models

**Figure 1:**
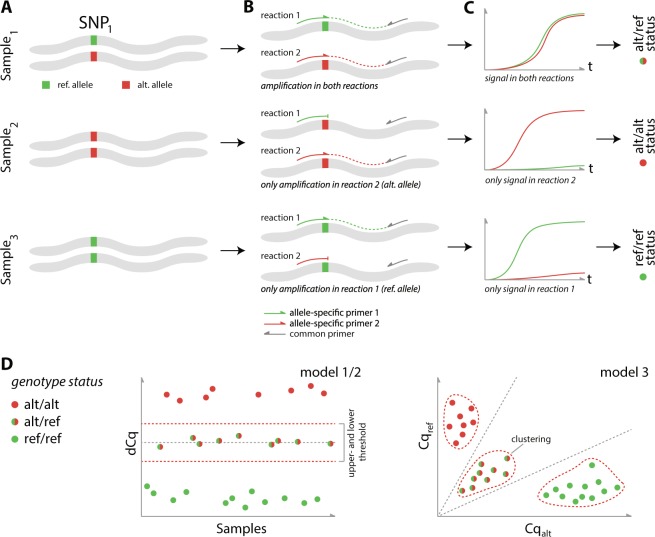
DMAS qPCR principle. Each DMAS-qPCR assay consists of a common primer (grey) and allele-specific primers targeting either the REF (green) or ALT (red) allele. Allele-specific primers are located with their 3′ terminal end on the SNP of interest and include an additional mismatch at the fourth nucleotide from the 3′ terminal end. For each allele-specific primer, an individual PCR reaction is performed, in combination with the common primer in parallel. Depending on the genotype status of the sample, both (heterozygous sample) or only one of the allele-specific primers (homozygous sample) will result in elongation and the generation of a signal. (**B**) By combining the signal status of both reactions, the genotype status of the sample can be deduced. (**C**) In models 1 and 2, this is achieved using the difference in Cq values from both reactions/signals (heterozygous samples will result in a dCq value approximating zero). In model 3, the Cq values of both reactions are plotted on opposite sides of a scatter plot, followed by genotype calling by means of clustering (**D**).

Each DMAS qPCR assay, consists of reference (REF) and alternative (ALT) allele specific primers, matching the SNP at their 3′ and containing an additional mismatch at the 4^th^ nucleotide of this end, and a common primer. Both combinations are used in parallel qPCR reactions and allows SNP detection by analysis of Cq values retrieved. Three genotyping models were proposed, all using the ΔCq – the difference in Cq between the REF and ALT allele reaction – from validated accessions to assign classify the SNP status. The first model (1) exploits setting manual thresholds around the heterozygous samples in the difference plots. Thresholds were based on critical assessment of the scatter and difference plots, found in Supplementary 1 and 2, while taking into account the expected status of all assessed cacao accessions e.g. Homozygotes (HOM) and Heterozygotes (HET). In the second model (2) the threshold in the difference plot was automatically set at µ ± 3xSD(H), with µ and SD(H) representing the average and standard deviation for all ΔCq values from the validated heterozygous samples (Supplementary 3). In this automatic threshold-based model, SNPs having ΔCq lying between these thresholds were called as heterozygous, with a 95% accuracy. The third model (3) employs unsupervised k-means clustering, pairing the REF and ALT Cq values. In this model, three distinct clusters could be differentiated, namely one for the heterozygous and two for the homozygous samples (ALT and REF allele). The SNP status of a sample was determined automatically based on the cluster it was assigned to (Fig. 1).

### DMAS-qPCR design and assay testing

For each of the 115 selected SNP targets, the SNP context sequence was extracted from literature and used to determine the exact genomic location on the cacao genome build by means of BLAST. The latter failed for 13 SNPs, including 12 disease-associated SNPs, due to incorrect/absent context sequences or non-specific alignments. For the majority (n = 88) of the 102 remaining SNP targets, genotype status of different cacao accessions has been published in ICGD, allowing comparison with DMAS-qPCR results later on. For the other 14 SNPs, genotype status was determined by means of in-house sequencing-based genotyping (SBG)^31^. DMAS-qPCR assay design was attempted for these 102 targets, for which 10 failed due to the limited design space and low genomic complexity at the associated regions. The remaining 92 DMAS-qPCR assays were first tested by qPCR on 6 to 10 cacao accessions for which a corresponding reference genotype status was available – through ICGD or SBG (Supplementary 4). In this phase, DMAS-qPCR-based SNP genotypes were determined by applying the first genotyping model, using the thresholds displayed in Supplementary File 3. Solely DMAS-qPCR assays for which all data complied with the available reference genotype status were retained for further validation. In total, 47 assays were able to discriminate the three SNP genotypes correctly (Supplementary 4).

### DMAS-qPCR screening and mislabeling detection

The additional 55 international cacao accessions were genotyped using the 47 successful DMAS-qPCR assays (Supplementary 5). Based on the initial results, five more SNPs were excluded from the set. Three of them (Tcm004s13887242, Tcm009s17898807 and TcSNP469) failed to make a clear differentiation in genotype status, while for the other two SBG predicted genotype status matched for less than 90% with the reference genotype status called (TcSNP25 and TcSNP1350) (data not shown). The final set of 42 validated DMAS-qPCR assays can be used to successfully genotype different international cacao accessions in a correct and easy manner.

Performance of each of these assays was assessed using their average ΔCq value and its standard deviation (SD) (Supplementary 3). Optimal assays can be recognized by overall low SD and low (approximating zero) and high absolute ΔCq values for heterozygous and homozygous samples, respectively. Higher absolute ΔCq values for homozygotes result in larger ΔCq_HOM/HET_ distances (i.e. abs(ΔCq_HOM_ − ΔCq_HET_) values) and thus increased discriminating power and more accurate genotype calling. When focusing on heterozygous calls, 92.9% (n = 39) assays showed an average ΔCq < 0.5 and 69.1% (n = 29) <0.25. For homozygous samples, ΔCq_HOM/HET_ values >5 could be observed for 92.9% (n = 39) and 88.1% (n = 37) of the assays in reference and alternative samples, respectively, while 69.1% (n = 29) and 57.1% (n = 24) of the ΔCq_HOM/HET_ distances exceeded 7.5. Overall, 78.6% of the assays generated ΔCq_HOM/HET_ >5 (for both alleles), allowing straightforward genotype calling. However, an assay having lower ΔCq_HOM/HET_ distances and very low SD for both the homozygous and heterozygous calls could still achieve similar (or even better) genotyping accuracy compared to an assay with perfect ΔCq_HOM/HET_ distances.

To assess this, all proposed genotyping models were applied on the obtained data and – for a subset of the accessions – results were compared with reference genotyping calls. Due to lack of sufficient heterozygous samples, no genotype could be called for TcSNP786 using the second model. Concordance of the genotype calls generated by each of the models with the reference status (i.e. ICGD or SBG) was overall high, from 89.97% for the second model (automated thresholds), over 91.51% for the third model (clustering), and up to 92.16% for the first model (manual threshold). In general, an overall average genotyping efficiency of 88.47% was observed when combining all models. By comparing models among themselves, similar trends could be observed with average genotype overlap reaching 97.60%. When taking the first model as a reference, the clustering-based model seems to outperform the automatic threshold-based model slightly (98.97% versus 98.46%, respectively). Overlap between the second and third model is substantially lower (97.75%) (Supplementary 3). For the remainder of the analysis, the genotype calls generated using the first model were selected due to its flexibility and their high concordance with the reference status.

During these analyses, an overall 15.38% mislabeling was detected by comparing the multi-locus SNP profiles of the accessions with their reference status. The reference status of ICS1(CEFORTT), LCTEEN62/S, IMC67, PA120, SCA9(CEFORTT), NA33, NA399 and SCA11 differed on more than 13 loci from our genotype calls and were thus classified as off-types. In contrast to Takrama *et al*. (2012) – who considered cacao clones as dissimilar when they differed on at least two loci^23^ – and Padi *et al*. (2015) – who permitted only a single mismatch to account for genotyping errors^9^, we only classified samples as off-type if they differed in at least four loci from the reference accession, e.g. EET 95 and U45. CEFFORT carried a greater amount of off-types in contrast to ICQC, with 24% and 9.76% respectively.

### Genetic diversity analysis

For all validated assays, key descriptive analysis was computed using GenAIEx v6.5^32^ (Table 1). At least three markers per linkage group (LG) were included in the validated marker set, which is important to accurately assess identity and genetic diversity. First, minor and major allele frequencies (*mAF* and *MAF*) were calculated, ranging from 0.046 and 0.945 to 0.492 and 0.508 with an average of 0.338 and 0.647, respectively. The large range of *MAF* suggests a good level of genetic diversity across the selected SNPs. This determines if a specific SNP is abundantly present in a population, making it useable as a selection criterion. From these frequencies expected and observed heterozygosity (*H*_*e*_ and *H*_*o*_*)* was calculated the first represents the proportion of heterozygosity expected under random mating and is proportional with genetic variability within the population, which should be high in a diverse population as the one used here whereas the second is inversely proportional with inbreeding. The *H*_*e*_ ranged from 0.088 to 0.500 (the highest possible value for biallelic SNPs) with an overall average of 0.416, while an average of 0.298 was observed for *H*_*o*_, ranging from 0.062 to 0.462. For all markers, except TcSNP878, *H*_*e*_ was higher than *H*_*o*_ suggesting an isolate-breaking effect (the mixing of two previously isolated populations).

**Table 1:** Key descriptive analysis of all validated loci on the analyzed international cacao accessions, LG = linkage group, mAF = minor allele frequency, MAF = Major alelel frequency, I = Shannons information index, H_o_ = observed heterozygosity, H_e_ = effective heterozygosity, F = fixation index, PIDsib = probability of identity siblings.

Locus	LG	mAF	MAF	Ne	Ho	He	I	F	PIDsib
TcSNP139	8	0.354	0.646	1.843	0.369	0.457	0.650	0.193	0.621
TcSNP144	10	0.408	0.592	1.934	0.323	0.483	0.676	0.331	0.605
TcSNP151	8	0.446	0.554	1.977	0.431	0.494	0.687	0.128	0.597
TcSNP174	4	0.133	0.867	1.299	0.141	0.230	0.392	0.390	0.790
TcSNP193	9	0.414	0.586	1.943	0.391	0.485	0.678	0.195	0.603
TcSNP226	9	0.246	0.754	1.590	0.246	0.371	0.558	0.337	0.681
TcSNP230	10	0.492	0.508	2.000	0.462	0.500	0.693	0.077	0.594
TcSNP364	9	0.146	0.854	1.333	0.200	0.250	0.416	0.199	0.774
TcSNP372	4	0.254	0.746	1.610	0.323	0.379	0.567	0.147	0.675
TcSNP413	3	0.400	0.600	1.923	0.338	0.480	0.673	0.295	0.606
TcSNP448	4	0.192	0.808	1.451	0.138	0.311	0.490	0.554	0.726
TcSNP577	5	0.485	0.515	1.998	0.415	0.500	0.693	0.168	0.594
TcSNP591	1	0.392	0.608	1.911	0.323	0.477	0.670	0.322	0.608
TcSNP602	6	0.215	0.785	1.511	0.277	0.338	0.521	0.181	0.705
TcSNP606	7	0.238	0.762	1.570	0.200	0.363	0.549	0.449	0.686
TcSNP751	5	0.208	0.792	1.491	0.231	0.329	0.511	0.299	0.712
TcSNP786	1	0.046	0.954	1.097	0.062	0.088	0.187	0.301	0.915
TcSNP852	3	0.323	0.677	1.777	0.246	0.437	0.629	0.437	0.634
TcSNP860	2	0.359	0.641	1.853	0.344	0.460	0.653	0.253	0.619
TcSNP872	4	0.446	0.554	1.977	0.277	0.494	0.687	0.440	0.597
TcSNP878	3	0.215	0.785	1.511	0.369	0.338	0.521	−0.092	0.705
TcSNP891	2	0.354	0.646	1.843	0.277	0.457	0.650	0.394	0.621
TcSNP994	6	0.485	0.515	1.998	0.354	0.500	0.693	0.292	0.594
TcSNP1041	10	0.154	0.846	1.352	0.215	0.260	0.429	0.173	0.765
TcSNP1060	2	0.215	0.785	1.511	0.308	0.338	0.521	0.090	0.705
TcSNP1111	5	0.223	0.777	1.531	0.200	0.347	0.531	0.423	0.698
TcSNP1126	7	0.092	0.908	1.201	0.154	0.168	0.308	0.082	0.843
TcSNP1280	1	0.408	0.592	1.934	0.323	0.483	0.676	0.331	0.605
TcSNP1331	10	0.477	0.523	1.996	0.400	0.499	0.692	0.198	0.594
TcSNP1439	9	0.262	0.738	1.629	0.308	0.386	0.575	0.203	0.670
TcSNP1458	1	0.485	0.515	1.998	0.262	0.500	0.693	0.476	0.594
Tcm002s00644224	2	0.446	0.554	1.977	0.338	0.494	0.687	0.315	0.597
Tcm002s29938429	2	0.400	0.600	1.923	0.462	0.480	0.673	0.038	0.606
Tcm002s34015437	2	0.385	0.615	1.899	0.277	0.473	0.666	0.415	0.611
Tcm003s12502217	3	0.438	0.562	1.970	0.415	0.492	0.686	0.156	0.599
Tcm003s20315420	3	0.400	0.600	1.923	0.277	0.480	0.673	0.423	0.606
Tcm003s05554949	3	0.408	0.592	1.934	0.262	0.483	0.676	0.458	0.605
Tcm003s27807955	3	0.485	0.515	1.998	0.354	0.500	0.693	0.292	0.594
Tcm006s26507164	6	0.423	0.577	1.954	0.385	0.488	0.681	0.212	0.601
Tcm008s17168944	8	0.477	0.523	1.996	0.400	0.499	0.692	0.198	0.594
Tcm009s28255143	9	0.438	0.562	1.970	0.262	0.492	0.686	0.469	0.599
Tcm009s41415628	9	0.469	0.531	1.992	0.415	0.498	0.691	0.166	0.595
Mean	—	0.336	0.647	1.726	0.297	0.409	0.591	0.266	3.4 × 10^−22^*
SE	—	0.127	0.147	0.365	0.100	0.118	0.146	0.143	/

*Accumulated PIDsibs for 42 SNP locus combinations.

Additionally, Shannon’s information index (*I)* – representing the informativeness of each marker – ranged from 0.187 (TcSNP786) to 0.693 for multiple loci, with a high average of 0.601. Fixation index (*F)* values approximating zero are expected under random mating, while substantial positive values indicate inbreeding. Negative *F*-values indicate excess of heterozygosity due to negative assortative mating or selection for heterozygotes^32^. Here, an *F*-value of 0.277 was observed, indicated random mating in the population. Further, the discriminating power of the SNP loci was computed through the probability of idenitity amongst siblings (PIDsib) over all analyzed loci and 65 cacao accessions counting for 3.4 × 10^−22^, indicating that the chance of finding two individuals with the same genotype in the population was close to zero. Identification of the previously detected off-types through pair-wise multi-locus analysis using all SNP data accessible in ICGD failed^33^ (data not shown). However, two duplicates could be identified, namely APA4 and SPEC41/6-18 – and – U45 and U70. Following ranking of the markers according to *I*, only 13 markers (TcSNP230, TcSNP577, TcSNP994, TcSNP1458, Tcm003s27807955, TcSNP1331, Tcm008s17168944, Tcm009s41415628, TcSNP151, TcSNP872, Tcm002s00644224, Tcm003s12502217 and Tcm009s28255143, in increasing order of *I*) were deemed necessary for differentiation with over 99.999% certainty as analyzed by matching and unique genotypes (data not shown). Furthermore, the validated assays were on average 98.05% efficient in calling the correct SNP genotype from unmislabeled cacao accessions with a 95% certainty (Supplementary 6).

## Discussion

Most of the published genetic studies on cacao involve genotyping and genetic diversity studies using molecular markers. In addition to gene-mapping and the construction of genetic linkage maps, these are considered the main tool to assess genetic relationships among populations and individuals. Over the last years, SNP-based genotyping has been on the rise. Even though SNPs are less informative in comparison to SSR markers, they provide higher inter-laboratory allele calling consistencies and their results are less ambiguous as the error rate is much lower. SNP analyses are also relatively cheap, easy to analyze, more stable and amply present in the genome with a wide spread of applications. Various SNP-based genotyping methods are currently available, enabling high-throughput genotype calling without the need for electrophoresis^34^. Many SNP based genotyping methods – specific for cacao research – have been described in literature, ranging from TaqMan-based assays^12,16,23^, over Maldi TOF MS^6,24,35^, and Fluidigm SNP genotyping^15,36,37^ to the competitive allele specific PCR KASPar chemistry^9^. Yet, no standard SNP genotyping method has been proposed for cacao genetic diversity studies. A drawback of these methods is that they often require expensive machinery or consumables, making their application in the poorer cacao-producing countries unfeasible. In this context, DMAS-qPCR can be considered a good alternative^29^. By combining fluorescent DNA binding dyes with qPCR readout, cost-efficient genotyping – rivaling similar TaqMan-based methods when looking at specificity and sensitivity – can be achieved.

First, literature was mined for known SNPs in *T. cacao*, taking into account several criteria. Since available chromosomal locations are often lacking, inaccurate or ambiguous, published context sequences were aligned to the cacao genome to determine the exact SNP location. However, when comparing results to the annotation available in ICGD, chromosomal positions did not seem to match and a systematic 60 bp shift could be observed for some of the SNPs. Correct positions were obtained from the authors and have been listed in Supplementary 4^26^.

DMAS-qPCR design was attempted for 102 SNPs, which failed for 10 markers. These failures could be attributed to the setup of DMAS-qPCR requiring allele-specific primers to align with their 3′ terminal nucleotide on the SNP of interest. This leaves little room to reposition the oligo, thus only its length, desired annealing temperature and GC content can be adjusted. Secondly, the cacao genome is less well annotated, only two genome builds are currently available^38,39^. Chromosomal regions with low complexity or uncertain nucleotide content are bound to hamper efficient assay design. After testing the assays, an additional set of 45 SNPs was excluded from further analyses and wet-lab testing, mostly due to amplification failure or limited discrimination/genotyping power (i.e. small ΔCq_HOM/HET_ distances, observed in ~38% of the markers). Different explanations for the high wet-lab failure rate can be proposed. Reaction conditions could be sub-optimal. Although the most critical factors known to affect PCR efficiency were taken into account during assay design, it is currently still difficult to accurately simulate reaction dynamics *in silico*. Another explanation includes unanticipated variation, such as polymorphisms, present in the primer sites. This could be confirmed for some failed assays – e.g. TcSNP90, TcSNP702 and TcSNP1439 – through targeted sequencing of the primer annealing sites. Variation in primer annealing sites has been shown to impede proper binding of a primer to its target, thus blocking or hampering efficient amplification, which could also be observed with the ASP-SNP genotyping method^30^. Since variation in *T. cacao* is less well characterized, this feature is most likely the major cause for some of the DMAS-qPCR failures.

Quality assessment and comparison of genotype calls obtained with the final set of 42 DMAS-qPCR showed the high reproducibility and robustness of the method, having a 98.05% overall efficiency. Global standard deviation for all assays across all samples was low, while discriminating power (ΔCq_HOM/HET_ ≥5) for most assays was sufficient to allow accurate genotyping. Evaluation of DMAS-qPCR-based genotyping accuracy was assessed by comparison with reference genotyping calls in corresponding accessions. Concordance of all tested genotyping models with reference calls was very high (88.47% on average), while overlap between the three models exceeded 97.60%. This shows genotyping efficiency and accuracy is independent of the classification method (manual vs automated), allowing automated calling approaches to streamline and simplify genotype calling. Discrepancies between DMAS-qPCR calls and reference genotype status could be explained by the uncertainty and inaccuracy associated with any experimental method, including DMAS-qPCR, and potential errors that could have slipped in ICGD – we have already identified duplicates in this repository as have others in the same or different collections and in greater amounts^15,40^. The level of inaccuracy associated with DMAS-qPCR can only be tested by applying this method on a sufficiently large sample population, and comparing the calls with results generated using multiple different techniques (e.g. TaqMan qPCR, sequencing and MS). The DMAS-qPCR results have shown to be independent from technical variations however, specificity could be increased by optimizing the additional internal mismatch, as shown by Hirotsu *et al*.^30^.

Following testing and validation, additional issues were encountered. It was noticed that for some SNPs, published annotation in ICGD showed the reverse complement call in comparison with the DMAS-qPCR status (e.g. A/G in ICGD, while T/C using DMAS-qPCR). It appeared some of the ICGD calls were called on the reverse strand, while DMAS-qPCR results were called on the forward strand. This is most likely caused by the fact that the current ICGD data was generated using EST libraries, and ICGD genotype status thus depends on the strand from which the gene was transcribed. More extensive annotation (e.g. template used, genotyping method, …) of genotype status in online repositories or adoption of common reporting guidelines (e.g. only report genotype calls on the forward strand) could prove helpful to prevent such discrepancies in future studies. As a start, the data from this study will be incorporated into the ICGD database to serve as an additional and independent source of genotype information for the corresponding samples.

Comparison of DMAS-qPCR results with publicly available status revealed 15.38% off-types in cacao accessions retrieved from CEFORTT and ICQC. This is undoubtedly less than the suspected level of mislabeling (15 to 44%) in germplasm collections worldwide^41^. Mislabeling and the verification of genetic identity from introduced as well conserved germplasm is an acute problem, as can be seen in this study and different publications over the last ten years^6,9,16,20,24,42^. Despite the fact that different applications and strategies on tackling cacao mislabeling have been provided by Turnbull *et al*.^43^, it still seems challenging to deploy these into practice. This underscores the need for accessible genotyping methods and off-type detection such as the one described in this paper. Introducing these guidelines to minimize mislabeling would facilitate and substantiate cacao research, management and breeding, by preventing the spread of undesired traits and guiding the choice of potential parents to minimize inbreeding^9^. DMAS-qPCR would allow straightforward and cost-efficient genotyping in the country of origin in a more routinely manner, thereby contributing to a more profound insight into the genomic identity of the cultured cacao varieties. Although a limited set of genotyping markers would improve adoption of this method by laboratories in service of local farms, a larger set is bound to better reflect the genomic reality and improve genotyping accuracy. So it is important to find the sweet spot between the marker set size and genotyping power. More studies as the one presented here will allow for better management of any material held in plant collections. Nevertheless, it should be taken into account that this method does not allow multiplex analysis, in contrast to TaqMan based assays. Furthermore, successful design of DMAS qPCR assays does not guarantee successful genotype discrimination, we have tested 102 assays resulting in a 54% overall failure rate.

According to Ji *et al*. (2013) and Livingstone *et al*. (2011), 26 SNPs would be adequate to uniquely identify a cacao clone in a population of 84 accessions and 19 clones, respectively. By selecting the most informative markers and analyzing unique and matching genotypes, it could be shown that a limited set of 13 markers is sufficient to achieve maximum differentiation in this population (n = 65). To obtain a more global insight in the genetic diversity and informativeness of these SNPs, more genotypes of different origins should be analyzed simultaneously. To further validate the applicability of the described DMAS-qPCR SNP assay in genetic diversity studies, analysis using PCoA and STRUCTURE^44^ is recommended, next to descriptive analysis by means of GenAIEx v6.5 to gain insights in marker as well as population characteristics^32^.

In conclusion, we have developed a robust and accurate method for cacao genotype identification using a limited set of SNPs. It can be applied to a wide range of genetic diversity studies, either with or without the inclusion of additional markers. The ease of use and cost-efficiency of the method – without the need of specialized instruments – can contribute to the adoption of routine-based genotyping to prevent mislabeling in germplasm collections and select optimal breeding parents in cacao and other organisms. The described method can easily be implemented in any molecular biology lab in the context of genotyping, genetic diversity studies, parentage analysis, mutation detection and to facilitate gene mapping and marker-assisted selection for breeding purposes.

## Material and Methods

### Plant material

Leaves from 65 international cacao reference accessions, representing the ten genetic groups of Motamayor *et al*.^41^ and seven *Trinitario* genotypes, were obtained from CEFORTT (Nong Lam University, Ho Chi Minh City, Vietnam – 25 samples) and ICQC (University of Reading, Reading, UK – 41 samples) (Table 2). The genetic identities of these accessions – except for U26, MO20, PA27, MO81, IMC53 and NA32 – have been characterized previously through an international initiative for DNA fingerprinting of cacao germplasm^33^ and published in ICGD. The leaf material (transported while wrapped in paper) was freeze-dried upon arrival (24 h, −40 °C, 0.11 mbar) using the ALPHA 1–2 LDplus instrument (Christ) and further crushed with liquid nitrogen, before storage at −18 °C.

**Table 2:** Accession number, name, source and genetic group of the analyzed cacao accessions.

Accession number	Name	Source	Genetic group
LCTEEN37/A	Londan Cocoa Trade Estacion Experimental Napo	CEFORTT	Curaray
NA32*	Nanay	CEFORTT	Iquitos
NA33	Nanay	CEFORTT	Nanay
ICS1	Imperial College Selections	CEFORTT	Trinitario^46^
LCTEEN62/S	Londan Cocoa Trade Estacion Experimental Napo	CEFORTT	unknown
IMC67	Iquitos mixed calabacillo	CEFORTT	Iquitos
PA120[PER]	Parinari	CEFORTT	Marañόn
SCA9	Sabino Contamana	CEFORTT	Contamana
MO81*	Morona	CEFORTT	Nacional
IMC53*	Iquitos mixed calabacillo	CEFORTT	Iquitos
PA127[PER]*	Parinari	CEFORTT	Marañόn
PA137[PER]	Parinari	CEFORTT	Marañόn
IFC5[CIV]	Institut Francais du Café et Cacao	CEFORTT	Forastero^47^
ICS43	Imperial College Selections	CEFORTT	Trinitario^46^
APA4	Amazonico Palmira	CEFORTT	UpperamazonForastero^48^
MA12[BRA]	Manaus	CEFORTT	Amelando
POUND16/B	Pound	CEFORTT	Nanay
MAN15/2[BRA]	MANaus	CEFORTT	LoweramazonForastero^49^
PA88[PER]	Parinari	CEFORTT	Marañόn
PA156[PER]	Parinari	CEFORTT	Marañόn
PA70[PER]	Parinari	CEFORTT	Marañόn
SCA6	Sabino Contamana	CEFORTT	Contanama
AMAZ15/15	Amazonas	CEFORTT	Iquitos
NA149	Nanay	CEFORTT	Nanay
SIAL339	Selecao Instituto Agronomicodo Leste	CEFORTT	Amelonado^50^
SPEC41/6-18	SPECimen	ICQC	Amelonado
FSC13	FSC	ICQC	Amelonado
LCTEEN302	Londan Cocoa Trade Estacion Experimental Napo	ICQC	Amelonado
GU114/P	Guiana	ICQC	Guiana
KER6	River KERinioutou	ICQC	Guiana
KER3	River KERinioutou	ICQC	Guiana
GU133/C	Guiana	ICQC	Guiana
GU261/P	Guiana	ICQC	Guiana
PA121[PER]	Parinari	ICQC	Marañόn
PA13[PER]	Parinari	ICQC	Marañόn
NA26	Nanay	ICQC	Nanay
NA232	Nanay	ICQC	Nanay
NA399	Nanay	ICQC	Nanay
SCA11	scavina	ICQC	Contamana
SCA9	scavina	ICQC	Contamana
U45[PER]	riverUcayali	ICQC	Contamana
U70[PER]	riverUcayali	ICQC	Contamana
IMC60	Iquitos Mixed Calabacillo	ICQC	Iquitos
IMC103	Iquitos Mixed Calabacillo	ICQC	Iquitos
COCA3370/5[CHA]	COCAriver	ICQC	Iquitos
Pound12/A	Pound	ICQC	Iquitos
LCTEEN163/A	Londan Cocoa Trade Estacion Experimental Napo	ICQC	Curaray
LCTEEN401	Londan Cocoa Trade Estacion Experimental Napo	ICQC	Curaray
LCTEEN261/S-4	Londan Cocoa Trade Estacion Experimental Napo	ICQC	Curaray
NAPO25[CHA]	Napo, Oriente,	ICQC	Curaray
RB47[BRA]	RioBranco.	ICQC	Purus
LCTEEN412	Londan Cocoa Trade Estacion Experimental Napo	ICQC	Purus
EBC148	Expedicion Botanico Caqueta	ICQC	Purus
RB46[BRA]	Rio Branco	ICQC	Purus
ICS1	Imperial College Selections	ICQC	Trinitario^46^
UF676	United Fruitselections	ICQC	Trinitario^46^
ICS95	Imperial College Selections	ICQC	Trinitario^46^
UF667	United Fruitselections	ICQC	Trinitario^46^
UF613	United Fruitselections	ICQC	Trinitario^46^
EET183[ECU]	Estacion Experimental Tropical	ICQC	Nacionalcrossings^33^
EET19[ECU]	Estacion Experimental Tropical	ICQC	Nacionalcrossings
EET95[ECU]	Estacion Experimental Tropical	ICQC	Nacionalcrossings
CRIOLLO21[CRI]	Criollo	ICQC	Criollo
MO20*	Morona	ICQC	Nacional
U26[PER]*	Ucayali	ICQC	Nacional
CRIOLLO11[CRI]	Criollo	ICQC	Criollo

The accessions are classified in different genetic groups according to Motamayor *et al*. (2008), if not stated otherwise. *Clones without published SNP genotype data.

### DNA extraction and quantification

Genomic DNA was isolated from lyophilized and crushed cacao leaf material with the Invisorb^®^ Spin Plant Minikit (Stratec). The protocol was executed according to the suggestions of the manufacturer, with following adaptations: (1) the addition and incubation of RNase A (20 mg/mL, Qiagen) for 5 minutes at room temperature after transferring the lysis solution to the Prefilter before adding binding buffer A and (2) two consecutive elution steps of 50 µL instead of a single 100 µL elution. After extraction, DNA was qualitatively and quantitatively analyzed using the Nanodrop 1000 (Thermo Scientific) and Quantus™ Fluorometer (Promega), respectively. All samples were normalized to 0.5 ng/µL.

### SNP selection

A total of 115 SNPs were initially selected from literature^6,12,24,27,33,36^, of which 12 have been associated with disease resistance^27^ (Supplementary 4). Selection criteria included the distribution across the ten chromosomes, the degree of polymorphism, their inclusion in previous cacao research and the availability of cacao genotyping data in ICGD.

### DMAS-qPCR

DMAS-qPCR assays were designed for only 102 SNPs, as 13 failed to map, using the GCA_00403535.1 cacao genome build (Matina 1–6) and the in-house developed primerXL web tool^45^. Each assay consists of one common primer and two AS primers, matching either the REF allele or the ALT allele, overlapping the SNP with their 3′ terminal nucleotide. An additional mismatch at position 3 (i.e. the fourth nucleotide from the primer 3′ end) was introduced to increase genotype discrimination power^29^ (Fig. 1). Primers were purchased from Integrated DNA Technologies (IDT, Carolvilla, Iowa). All DMAS-qPCR assays were run in duplicate on a LightCycler^®^ 480 Real time PCR System (Roche). In brief, each 6 µL reaction contained 1 µL common primer (1.25 µM), 1 µL AS primer (either the reference or alternative allele) (1.25 µM), 3 µL SsoAdvanced SYBR Green Supermix (2×) (Bio-Rad) and 1 µL template DNA (0.5 ng/µL). The following cycling parameters were used for all assays: (1) 2 min at 95 °C, (2) 44 cycles of 5 s at 95 °C, 30 s at 60 °C and 1 s at 72 °C. After amplification, Cq values were collected from the LightCycler^®^ 480 software to assess SNP genotype status by means of scatter and difference plots, as described by Lefever *et al*.^29^.

### Genotyping by MiSeq sequencing

For SNPs without annotation in ICGD, a reference genotype status in the samples was determined by means of SBG using a MiSeq instrument (Illumina). In brief, primers surrounding the SNPs were designed using primerXL, followed by PCR in the samples of interest. After library preparation and sequencing, the reads were aligned to the Matina 1–6 genome using the Burrows-Wheeler aligner (BWA). Reads were sorted and indexed with SAMtools and duplicates removed using Picard. Finally, variants in the aligned reads were identified using the Genome Analysis ToolKit (GATK). Through variant allele frequency (VAF) analysis, the ratio of the number of reads harboring the variant and the total read count are calculated. Subsequently, homozygous (~100% or ~0% VAF) and heterozygous (~50% VAF) SNPs could be differentiated,

### Key descriptive statistics

GenAIEx v6.5 was used to determine key descriptive statistics for the genetic markers tested on the available cacao population^32^. Nucleotides were given the following aliases in the analyses: A = 1, C = 2, G = 3, T = 4 and missing = 0. The program allows the computation of the *mAF*, *MAF* (1 − *mAF*), *I*, *H*_*o*_, *H*_*e*_, *F*, PIDsib and the analysis of matching and unique genotypes, amongst others. Output of the analysis was used to determine the minimum SNP set required to achieve maximum genotype power, sensitivity and specificity. Through pair-wise multi-locus matching, duplicates (synonymous mislabeling – accessions having identical SNP genotypes but different names) and off-types (homonymous mislabeling – accessions with different genotypes but same name) could be identified^24^. Three mismatches between DMAS-qPCR results and reference status were permitted to account for genotyping errors in each analyzed cacao accession.

## Supplementary information

Supplementary Dataset 1

Supplementary Dataset 2

Supplementary Dataset 3

Supplementary Dataset 4

Supplementary Dataset 5

Supplementary Dataset 6

## Data Availability

The datasets generated and/or analyzed during the current study are available in Supplementary, the primer sequences may be obtained from the corresponding author upon reasonable request. In addition, the SNP data retrieved will be submitted to the ICGD (http://www.icgd.reading.ac.uk/acknowledgements.php), hosted by the University of Reading.
