# Supplementary material for: The development of a novel SNP genotyping assay to differentiate cacao clones
Source: Sci Rep. 2019 Jul 2;9:9512. doi: 10.1038/s41598-019-45884-8 (PMC6606624; doi:10.1038/s41598-019-45884-8)
Supplement: Supplementary file 1 — Supplementary Dataset 1 [file 41598_2019_45884_MOESM1_ESM.pdf]

The development of a novel SNP genotyping assay to differentiate cacao clones

Jocelyn De Wever, Helena Everaert, Frauke Coppieters, Hayley Rottiers, Koen Dewettinck, Steve Lefever, Kathy Messens

Appendix 1: DMAS-qPCR Scatterplots

Two dimensional plot created by plotting the mean Cq values of the two parallel DMAS-qPCR reactions on opposite axes, either detecting the reference or alternative allele. In these plots, samples with a heterozygous genotype, having two low Cq values, are grouped in the lower left corner of the plot while homozygous samples appear either in the upper left or lower right corner. The plots below display the results of each DMAS-qPCR SNP assay used for genotyping different cacao accessions.

X/Y values

x-axis: Cq value reference allele for that specific SNP marker

y-axis: Cq value alternative allele for that specific SNP marker

Color code

Black: Left upper corner, Low Cq value for the reference allele in combination with a high Cq value for the alternative allele results in the following SNP genotype for that specific sample and SNP marker: homozygous for the reference allele.

Red: Left lower corner, Low Cq value for the reference allele in combination with a low Cq value for the alternative allele results in the following SNP genotype for that specific sample and SNP marker: heterozygous.

Green: Right lower corner, High Cq value for the reference allele in combination with a low Cq value for the alternative allele results in the following SNP genotype for that specific sample and SNP marker: homozygous for the alternative allele.

Table of content

1. TcSNP139
2. TcSNP144
3. TcSNP151
4. TcSNP174
5. TcSNP193
6. TcSNP226
7. TcSNP230
8. TcSNP364
9. TcSNP372
10. TcSNP413
11. TcSNP448
12. TcSNP577
13. TcSNP591
14. TcSNP602
15. TcSNP606
16. TcSNP751
17. TcSNP786
18. TcSNP852

19. TcSNP860
20. TcSNP872
21. TcSNP878
22. TcSNP891
23. TcSNP994
24. TcSNP1041
25. TcSNP1060
26. TcSNP1111
27. TcSNP1126
28. TcSNP1280
29. TcSNP1331
30. TcSNP1439
31. TcSNP1458
32. Tcm002s00644224
33. Tcm002s29938429
34. Tcm002s34015437
35. Tcm003s05554949
36. Tcm003s12502217
37. Tcm003s20315420
38. Tcm003s27807955
39. Tcm006s26507164
40. Tcm008s17168944
41. Tcm009s28255143
42. Tcm009s41415628

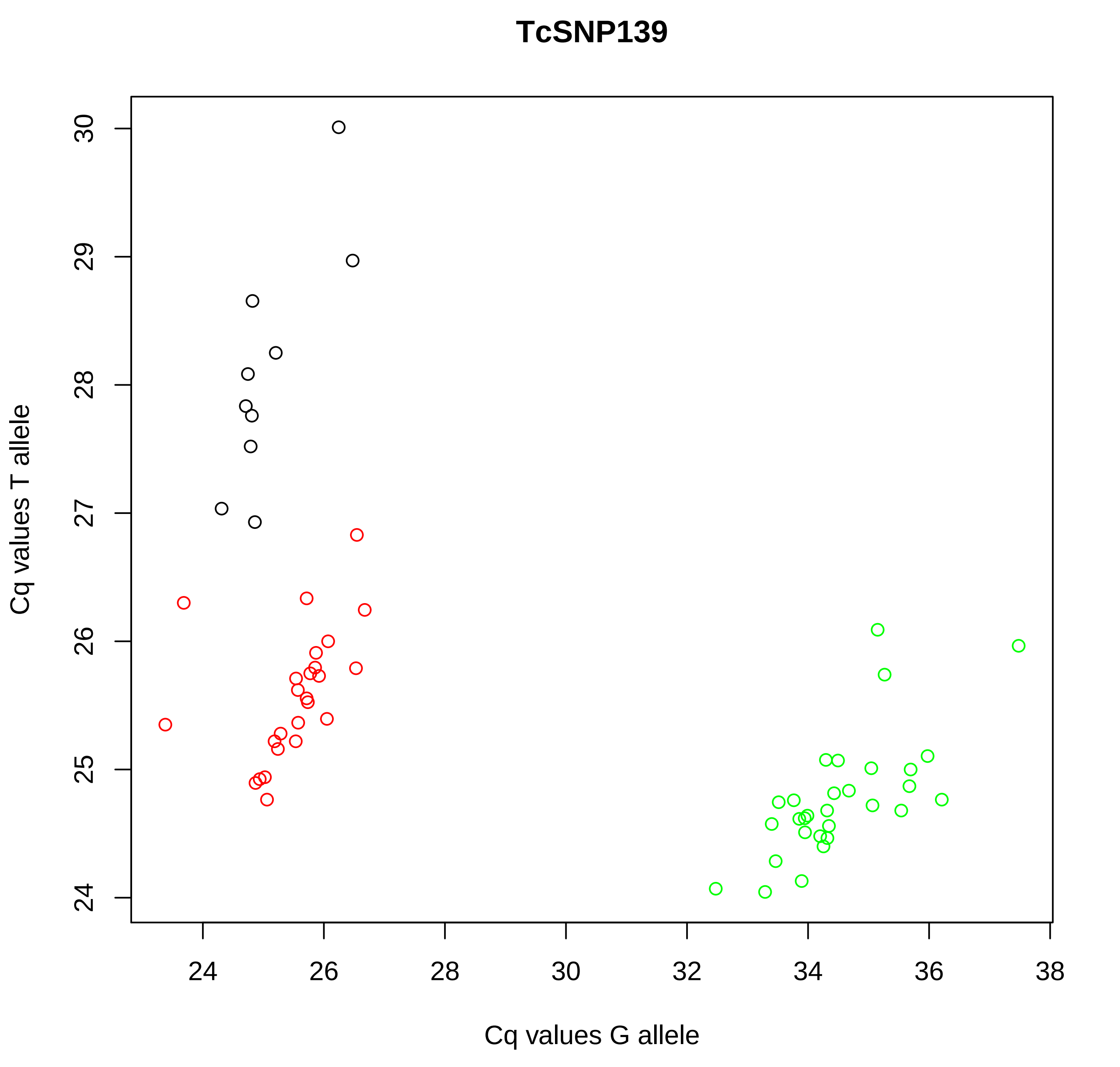
TcSNP139
Cq values T allele
Cq values G allele
24
25
26
27
28
29
30
24
26
28
30
32
34
36
38

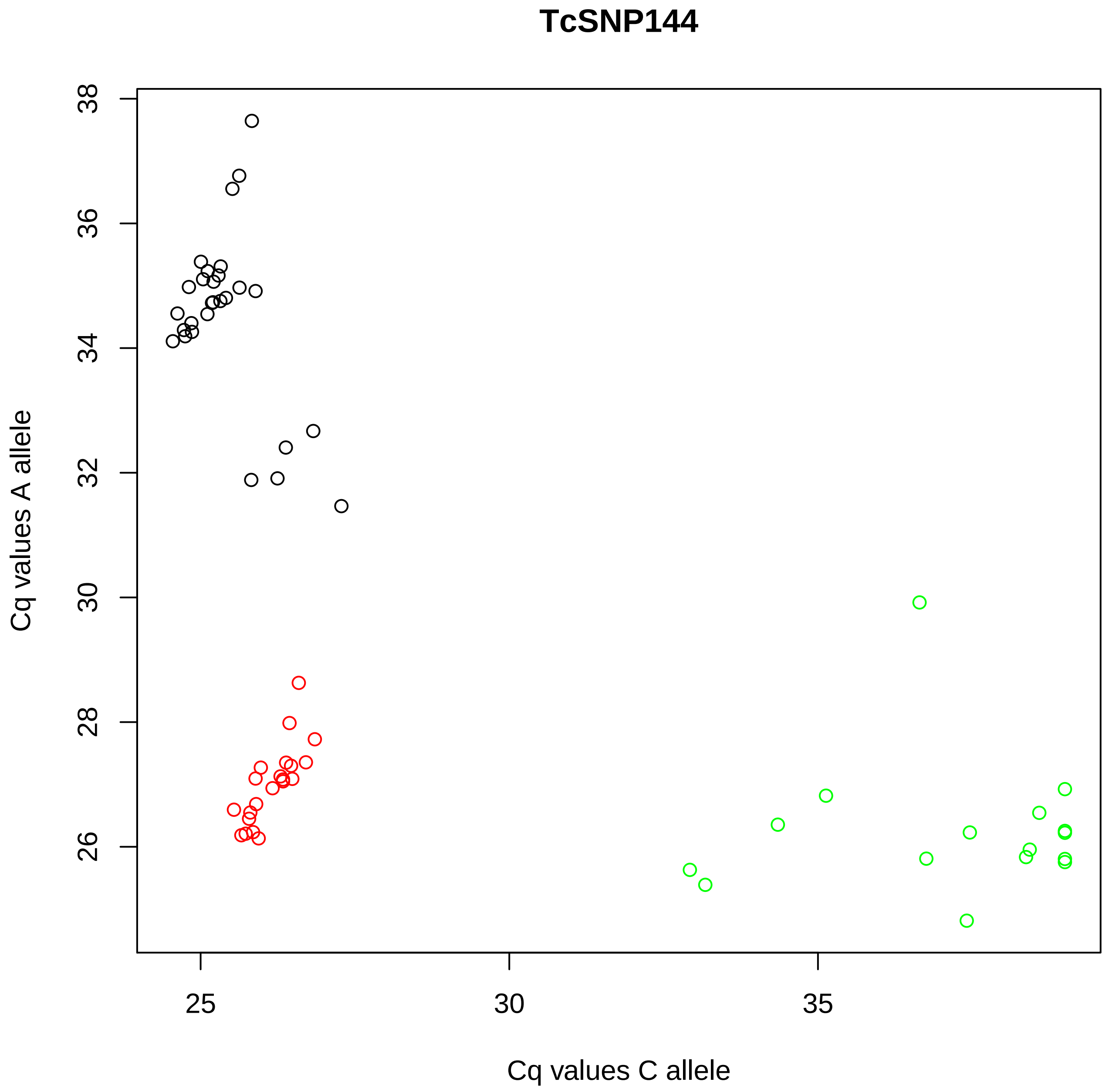
TcSNP144
Cq values A allele
Cq values C allele
38
36
34
32
30
28
26
25
30
35

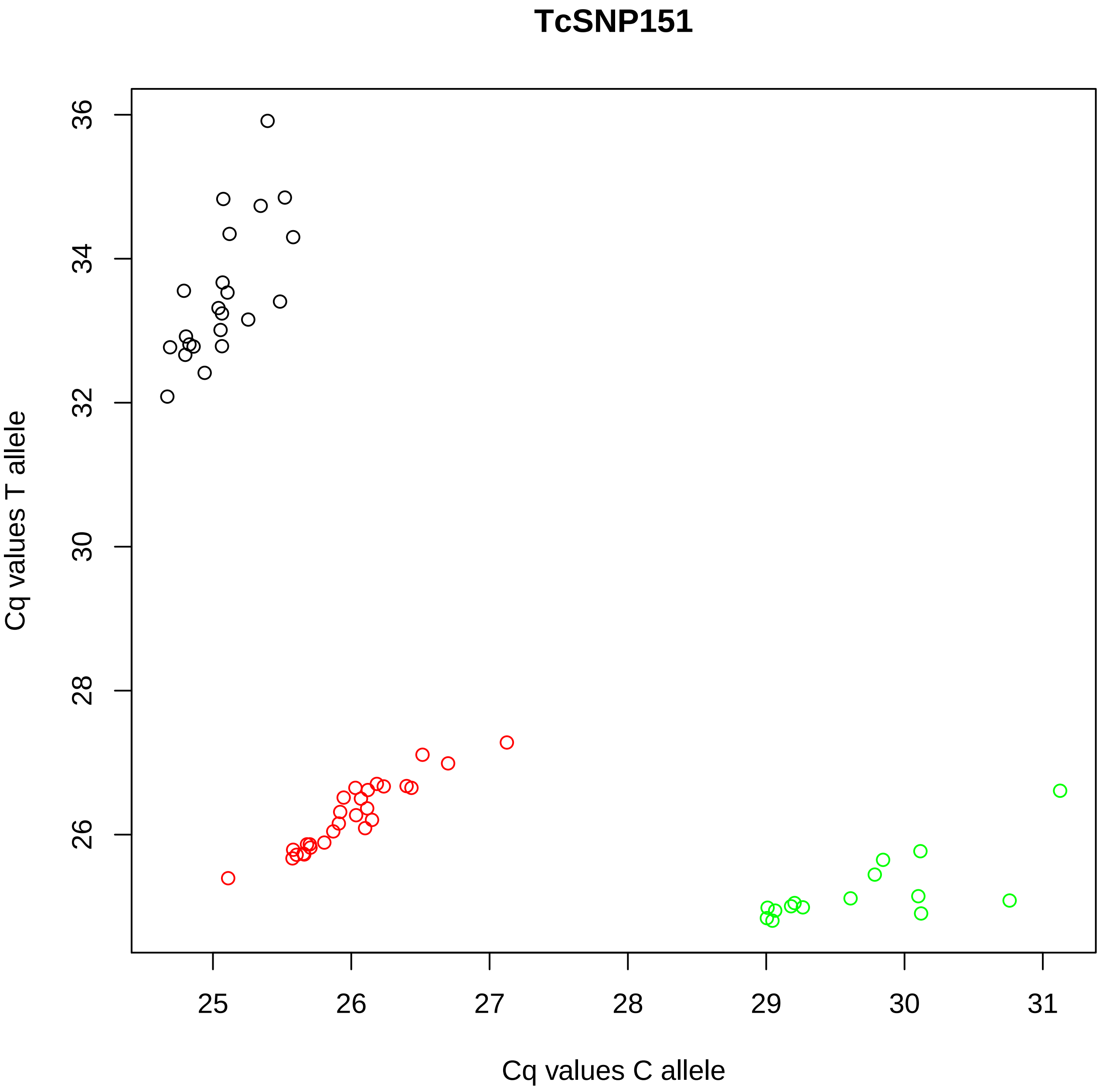

TcSNP151
Cq values T allele
Cq values C allele
36
34
32
30
28
26
25
26
27
28
29
30
31

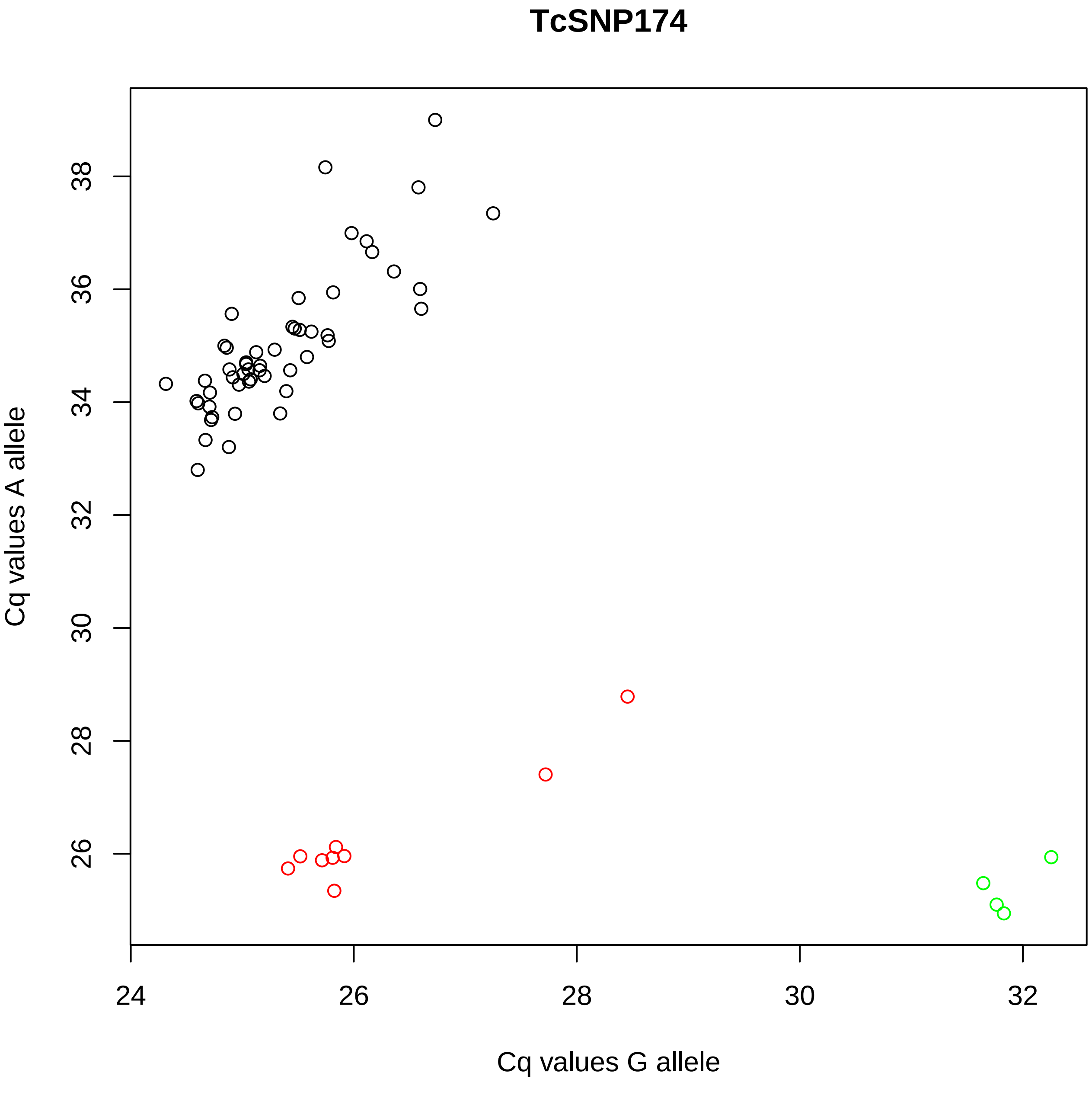

TcSNP174
Cq values A allele
Cq values G allele
24
26
28
30
32
26
28
30
32
34
36
38

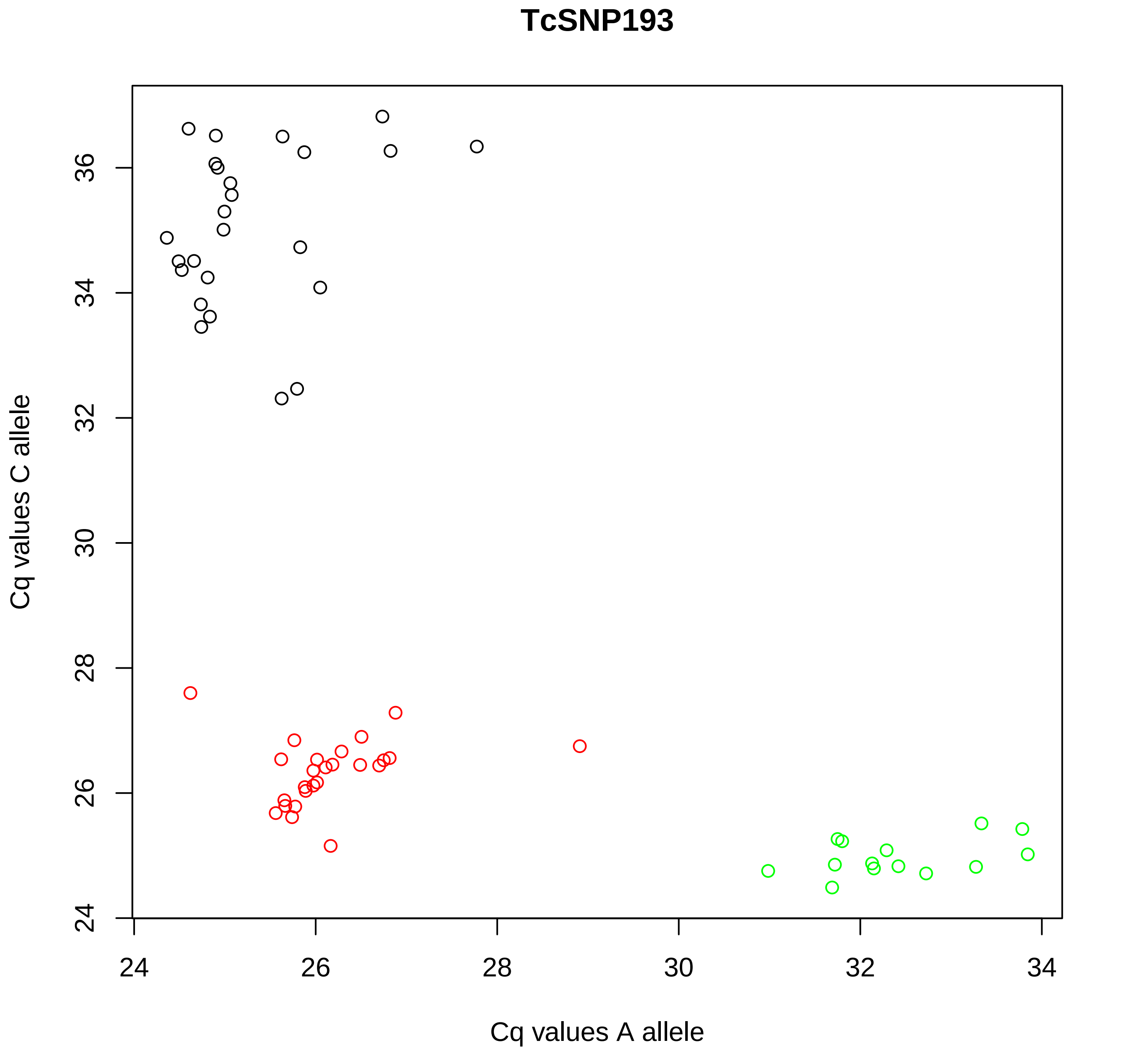
TcSNP193
Cq values C allele
Cq values A allele
24
26
28
30
32
34
36
24
26
28
30
32
34

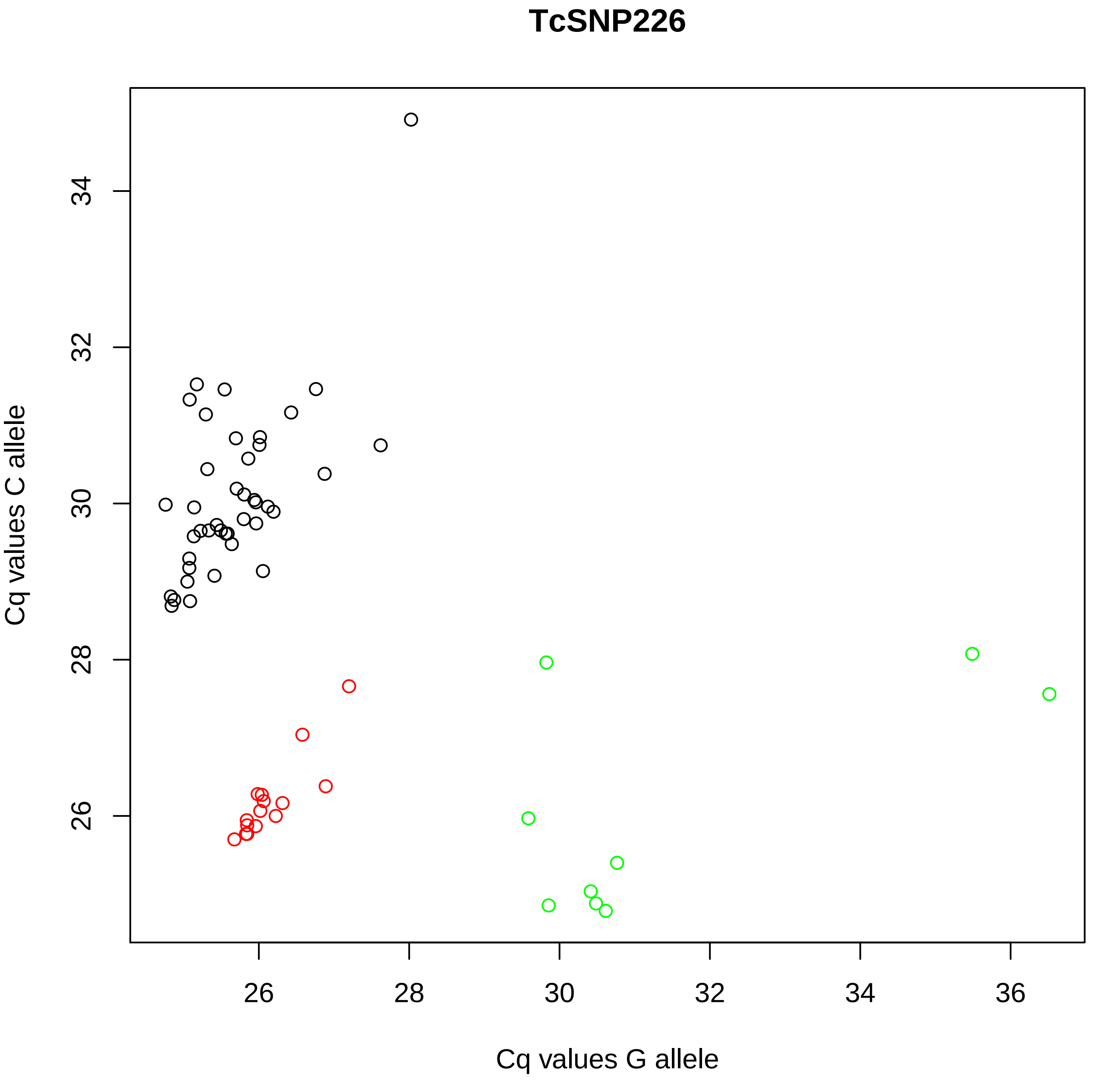
TcSNP226
Cq values C allele
Cq values G allele
26
28
30
32
34
36

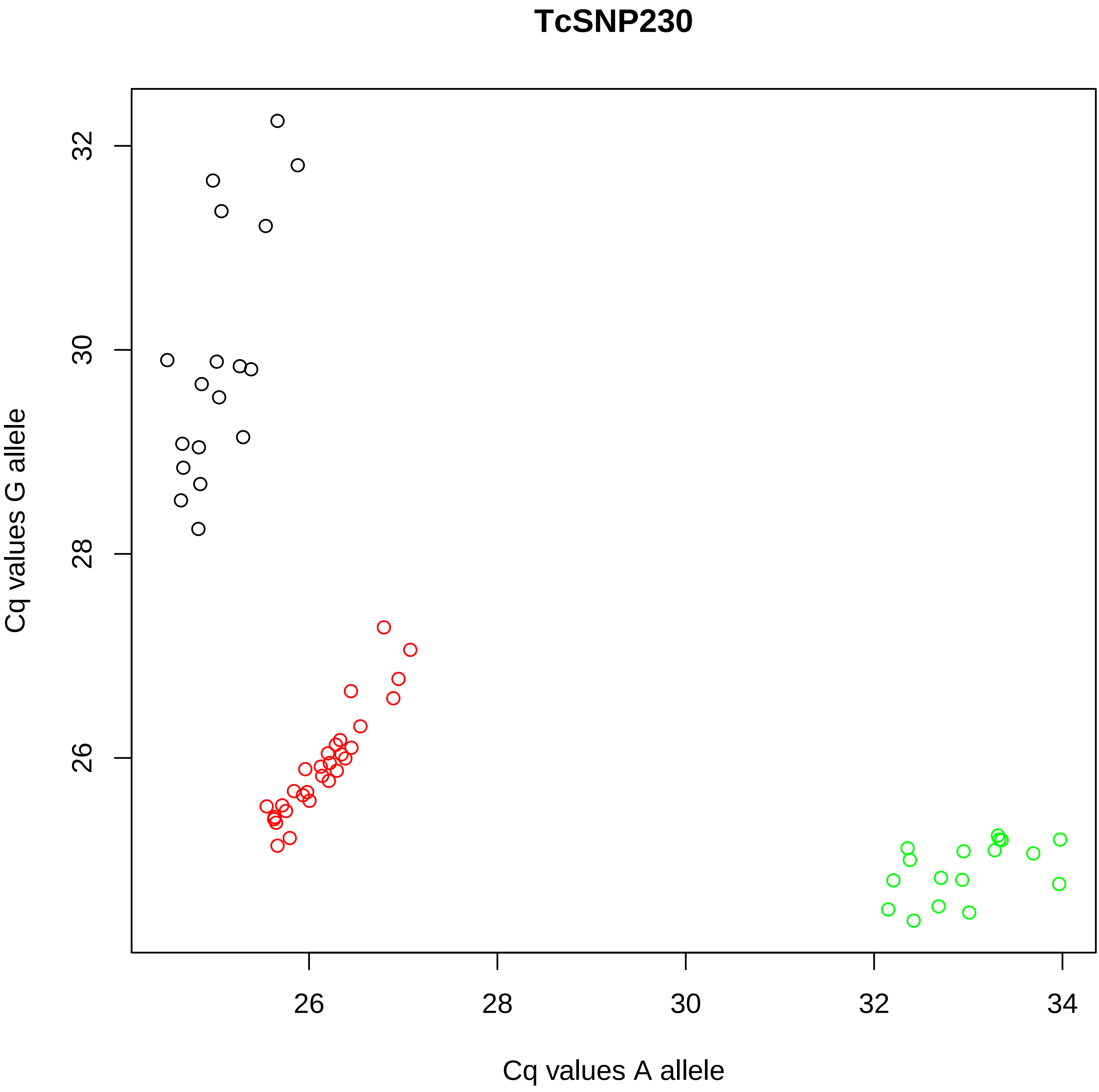
TcSNP230
Cq values G allele
Cq values A allele
26
28
30
32
34
26
28
30
32

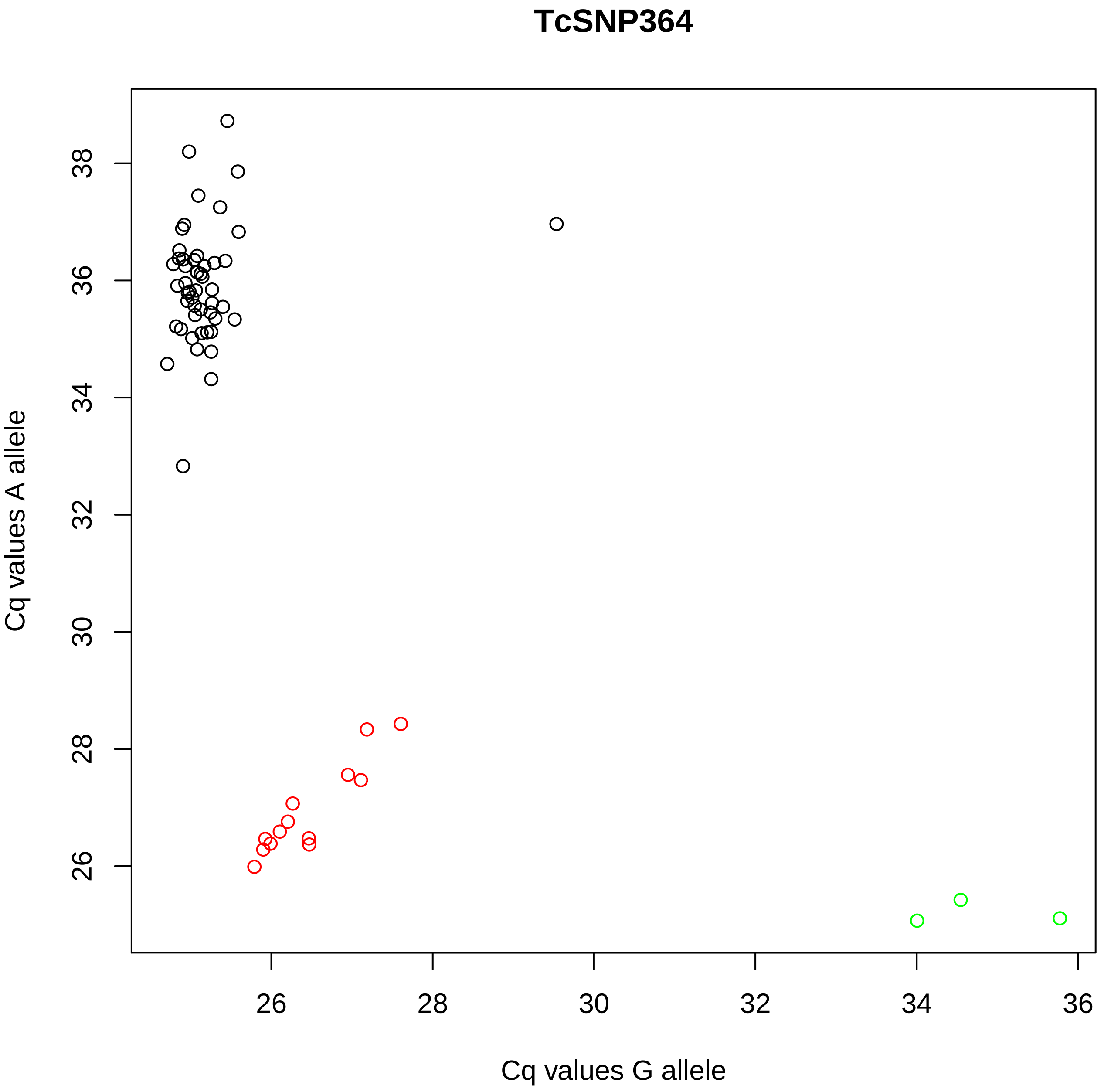
TcSNP364
Cq values A allele
Cq values G allele
26
28
30
32
34
36
38

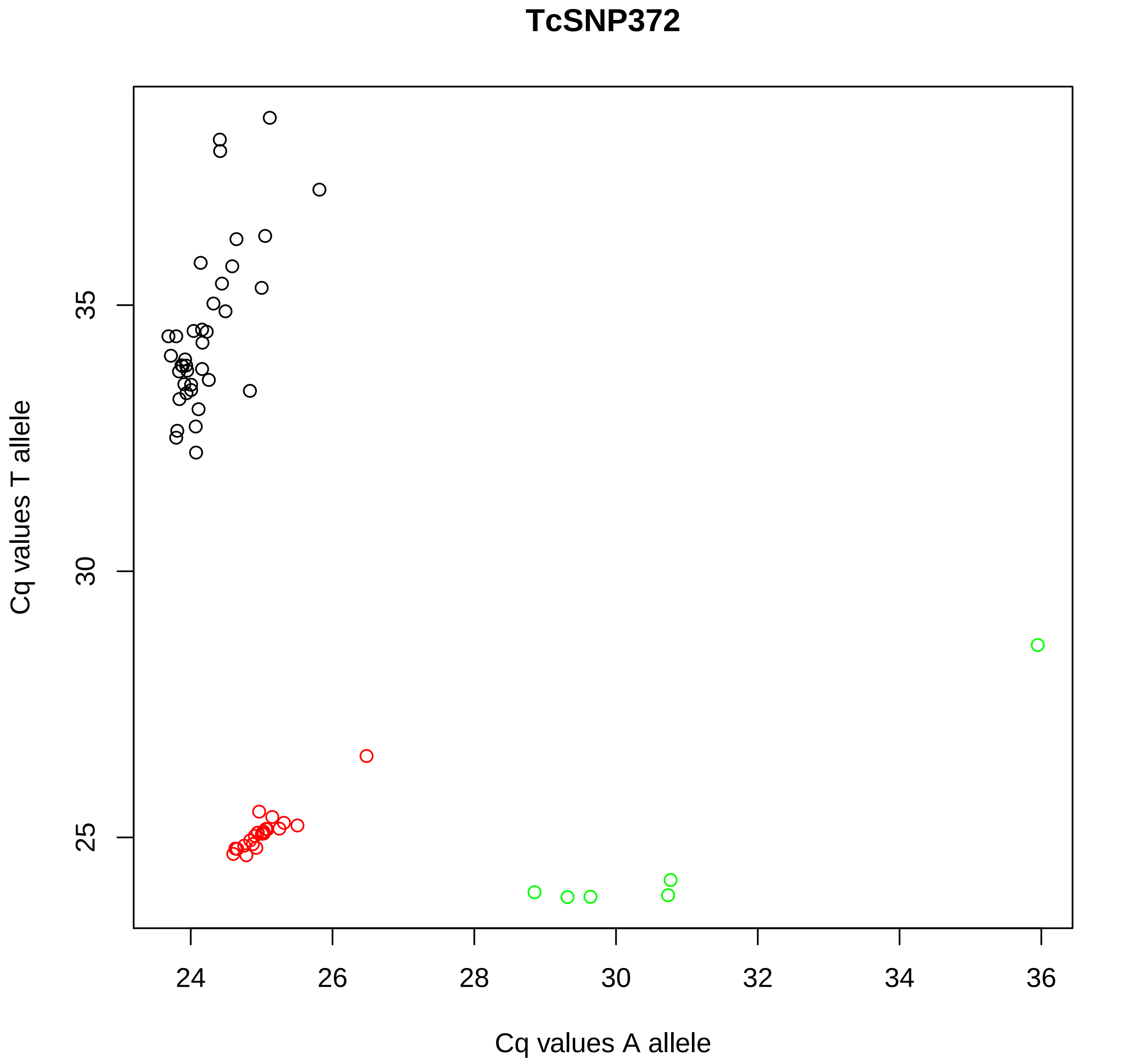
TcSNP372
Cq values T allele
Cq values A allele
35
30
25
24
26
28
30
32
34
36

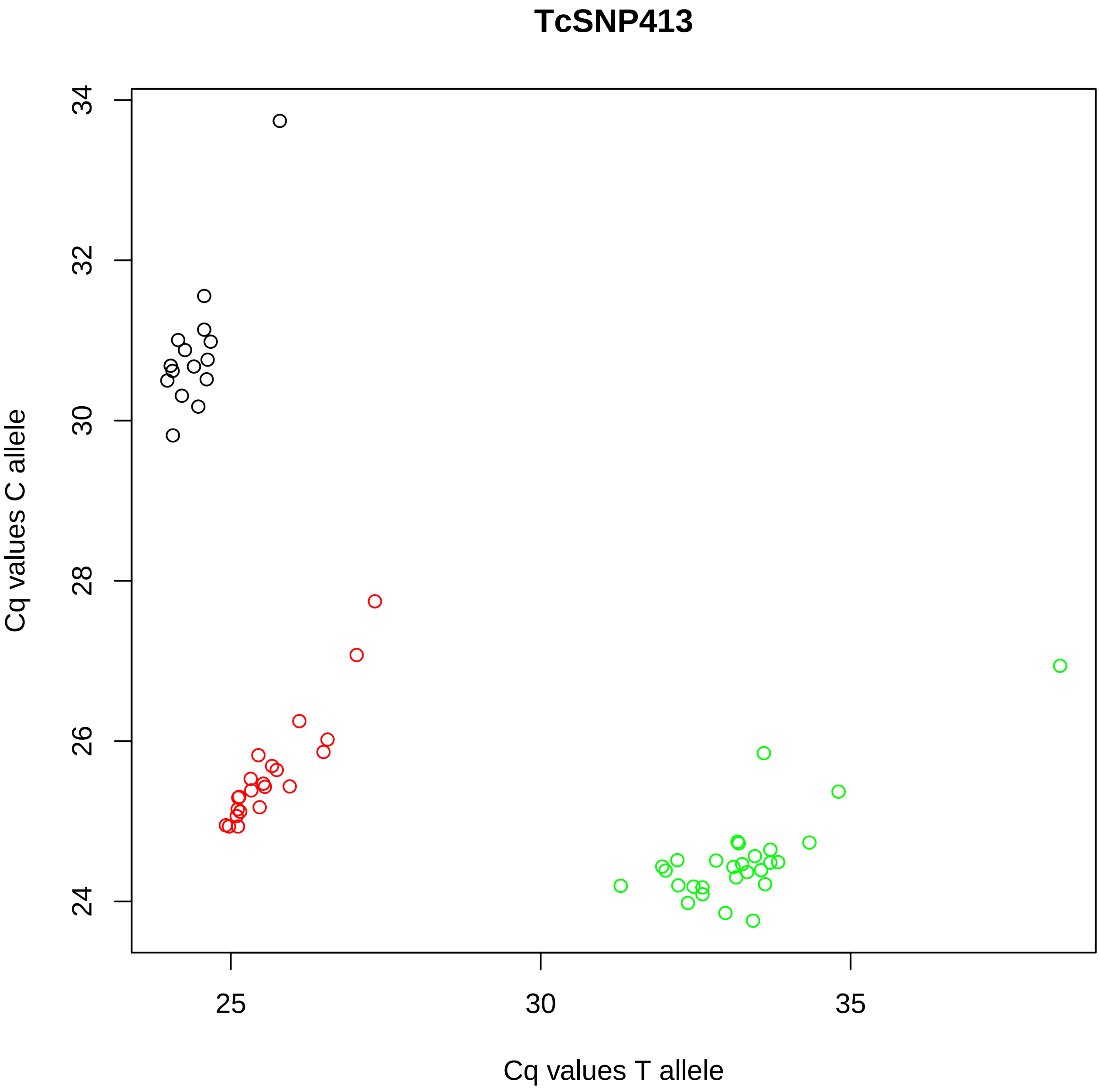
TcSNP413
Cq values C allele
Cq values T allele
34
32
30
28
26
24
25
30
35

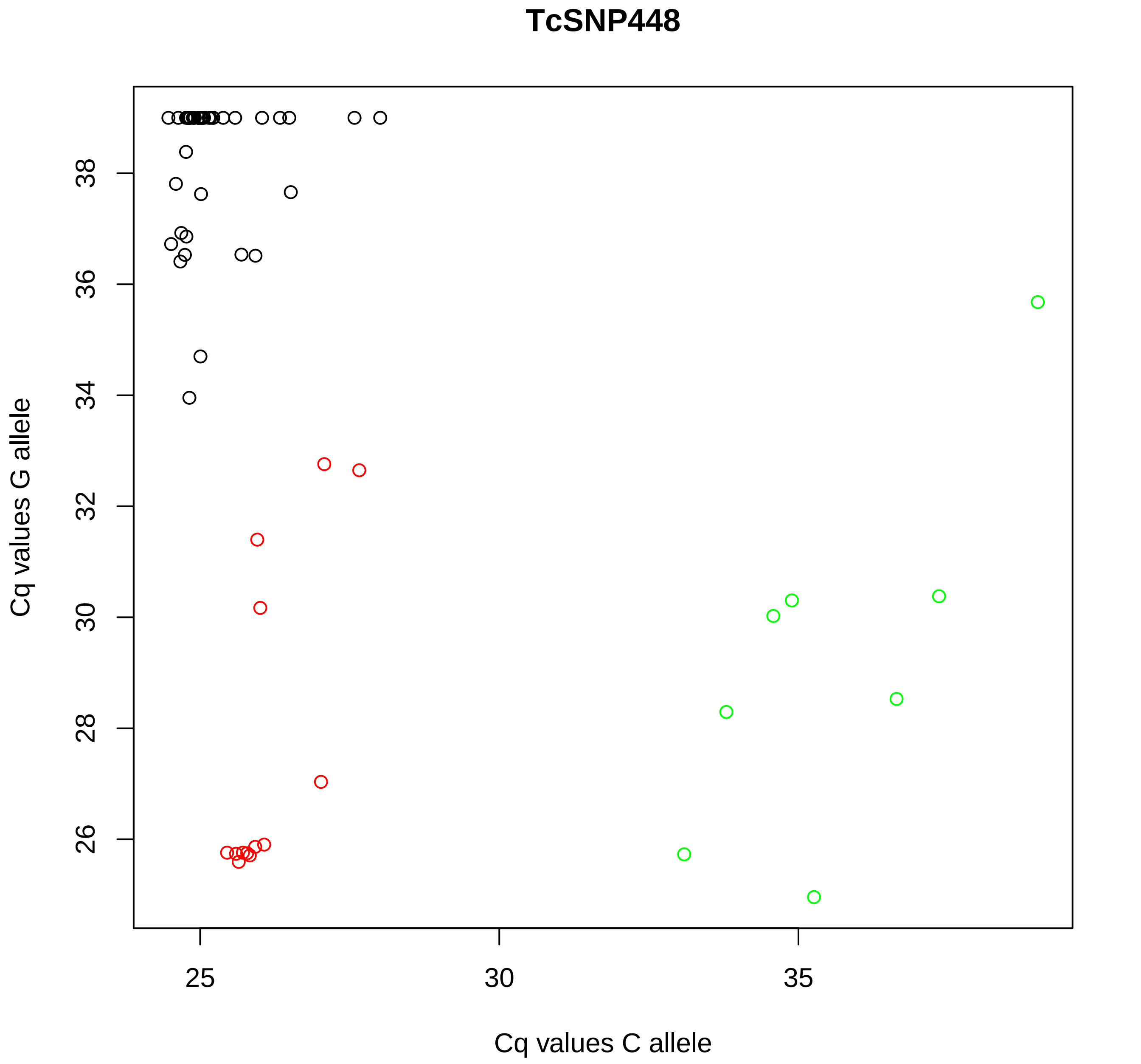
TcSNP448
Cq values G allele
Cq values C allele
26
28
30
32
34
36
38
25
30
35

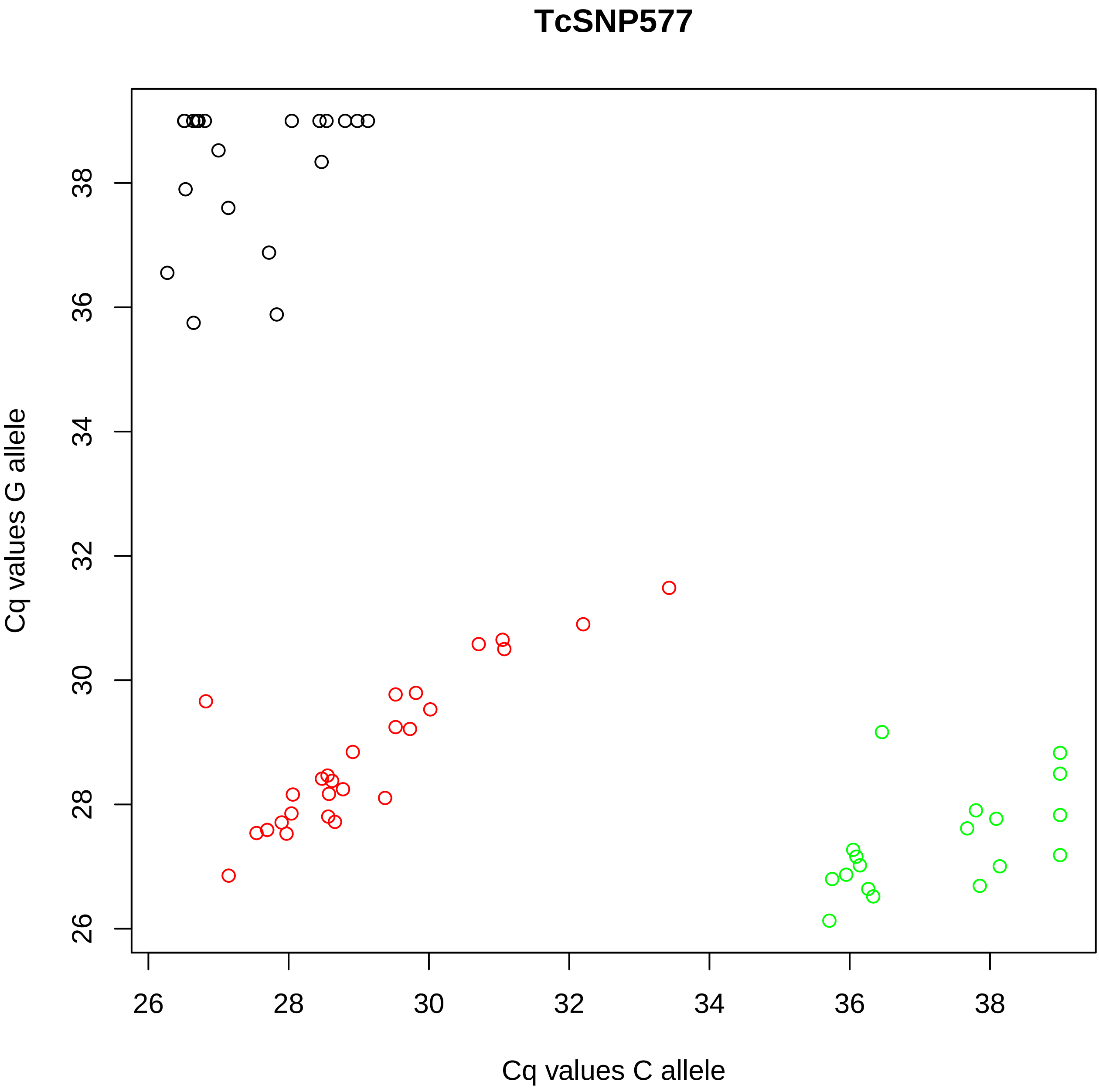

TcSNP577
Cq values G allele
Cq values C allele
26
28
30
32
34
36
38

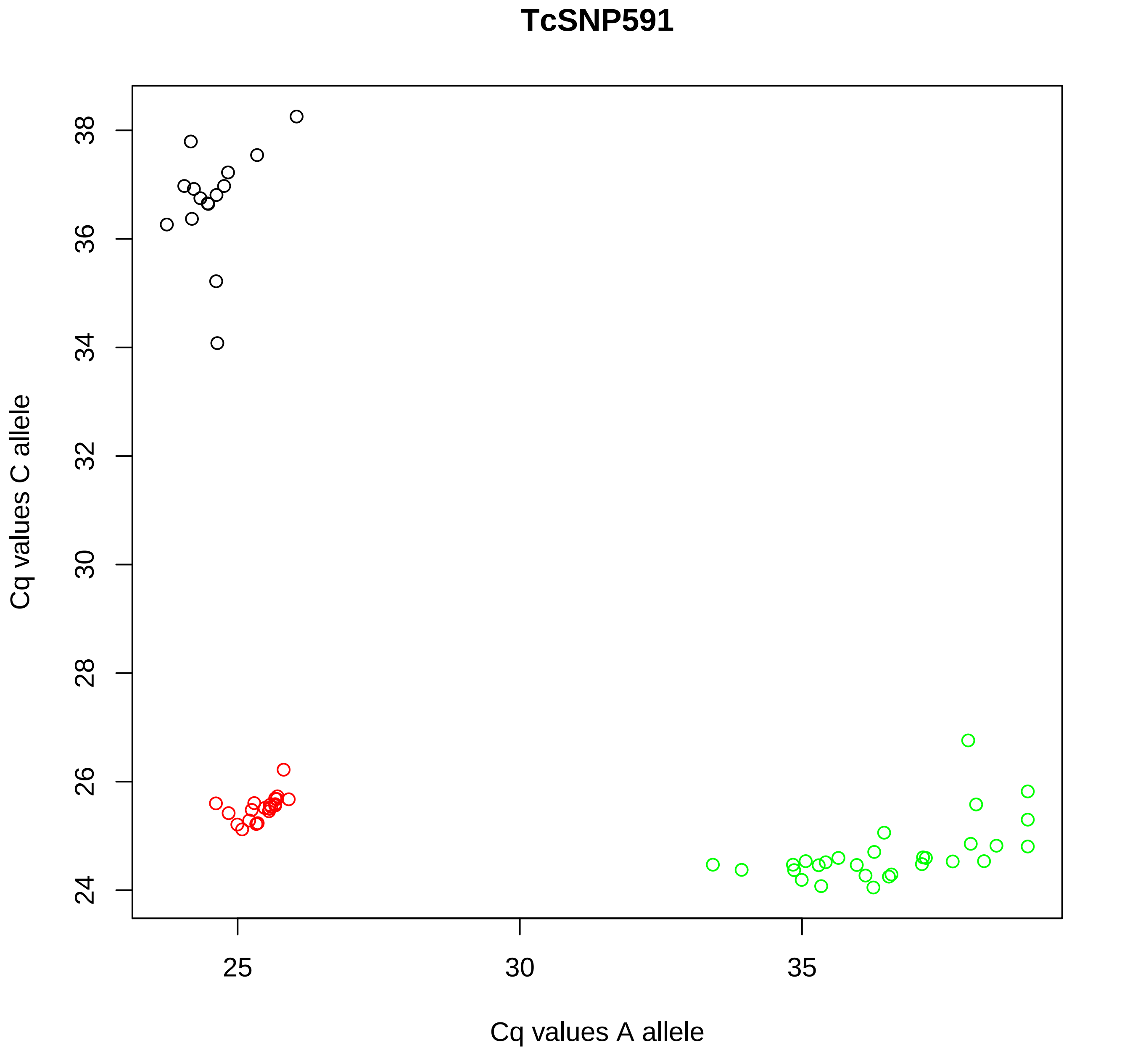

TcSNP591
Cq values C allele
Cq values A allele
24
26
28
30
32
34
36
38
25
30
35

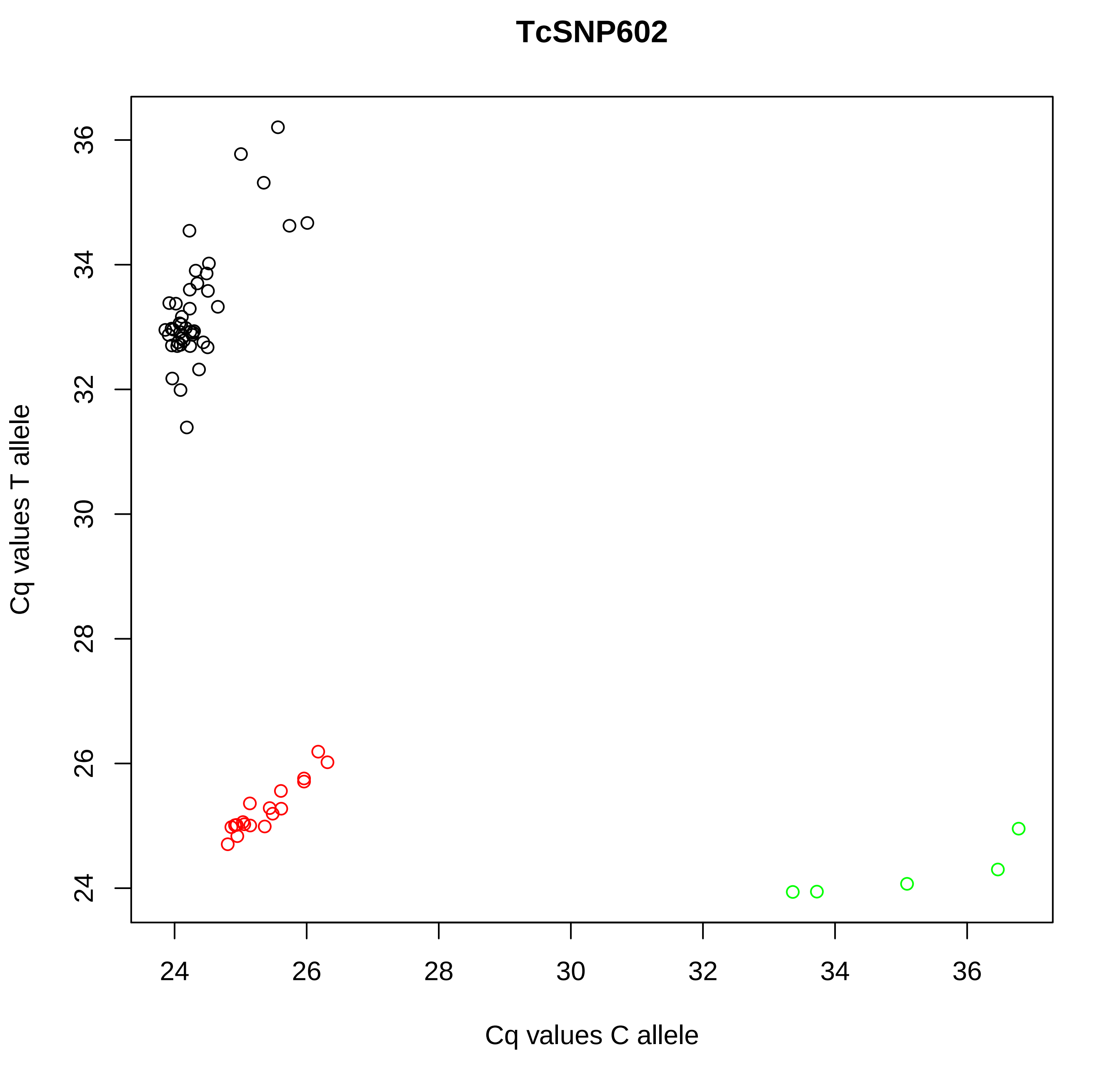
TcSNP602
Cq values T allele
Cq values C allele
24
26
28
30
32
34
36
24
26
28
30
32
34
36

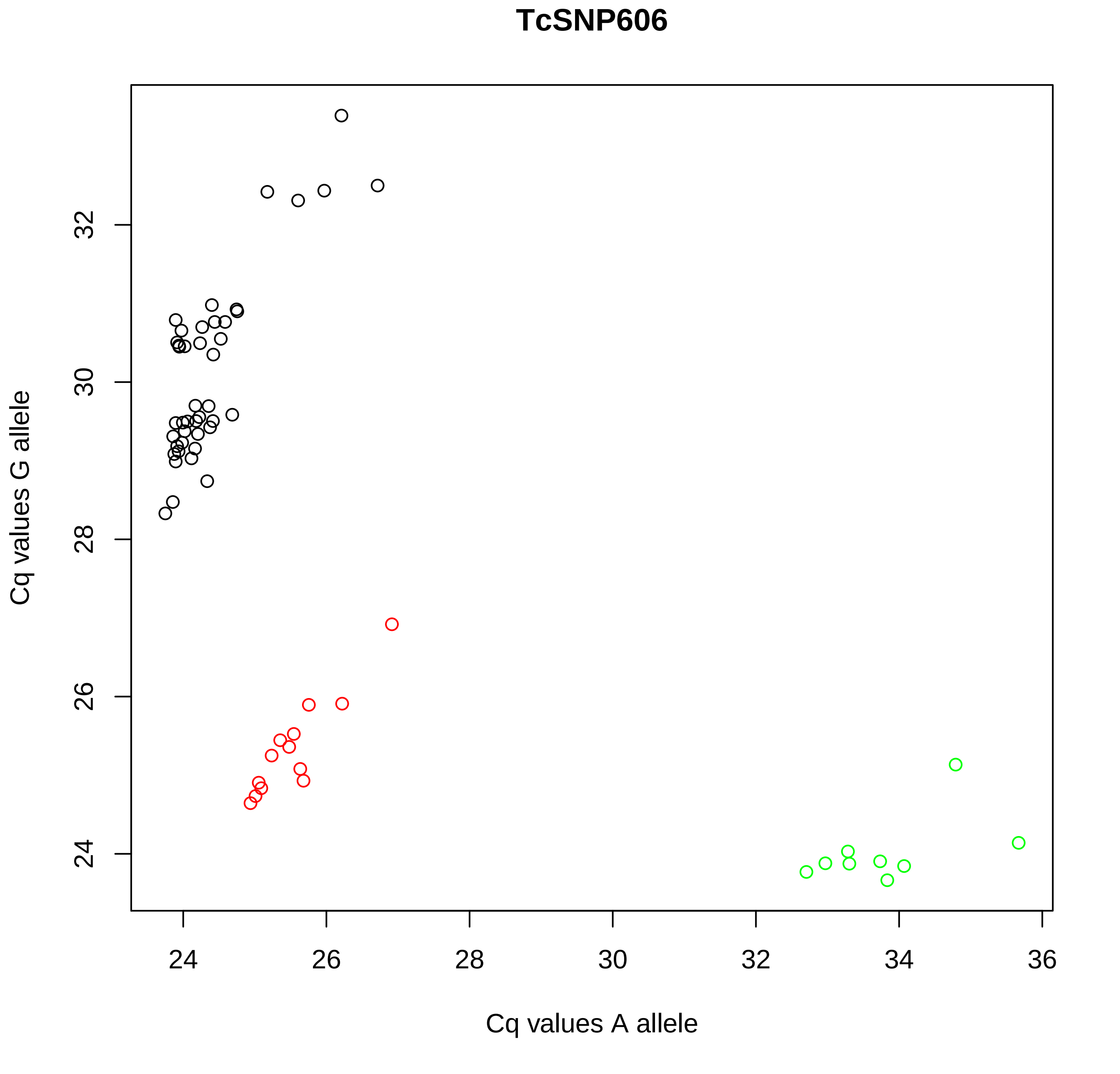

TcSNP606
Cq values G allele
Cq values A allele
24
26
28
30
32
34
36

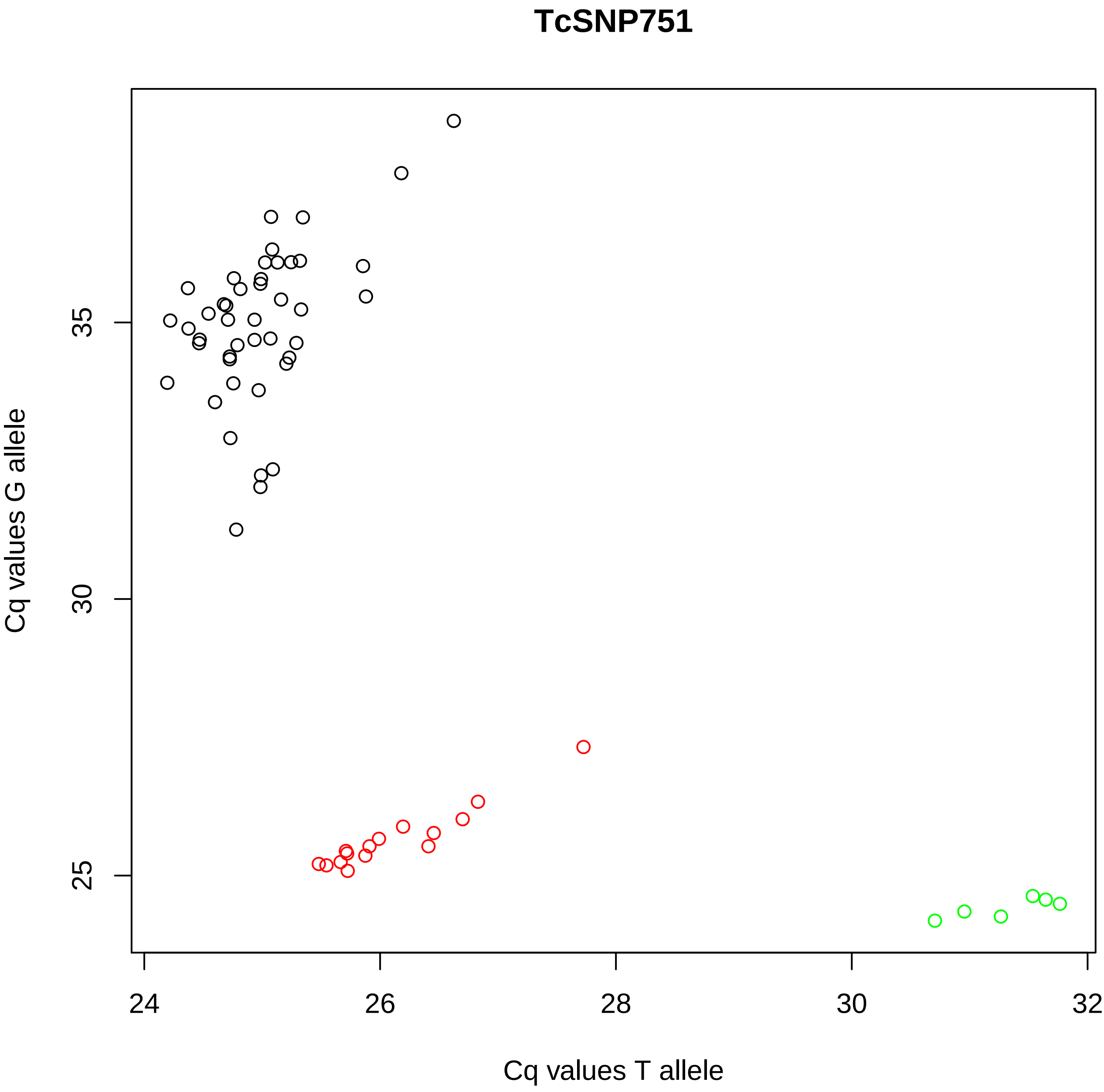
TcSNP751
Cq values G allele
Cq values T allele
25
30
35
24
26
28
30
32

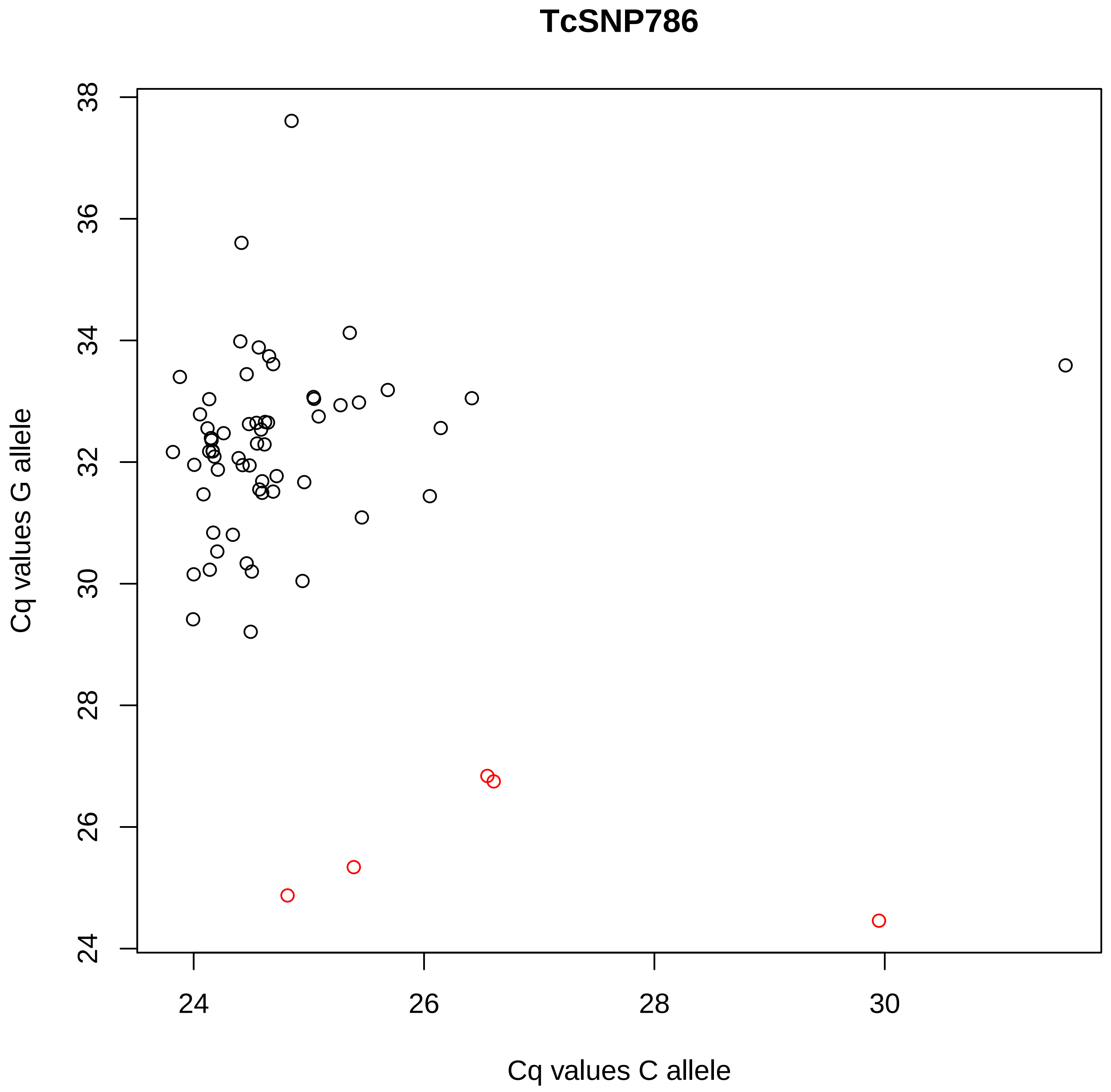

TcSNP786
Cq values G allele
Cq values C allele
24
26
28
30
32
34
36
38
24
26
28
30

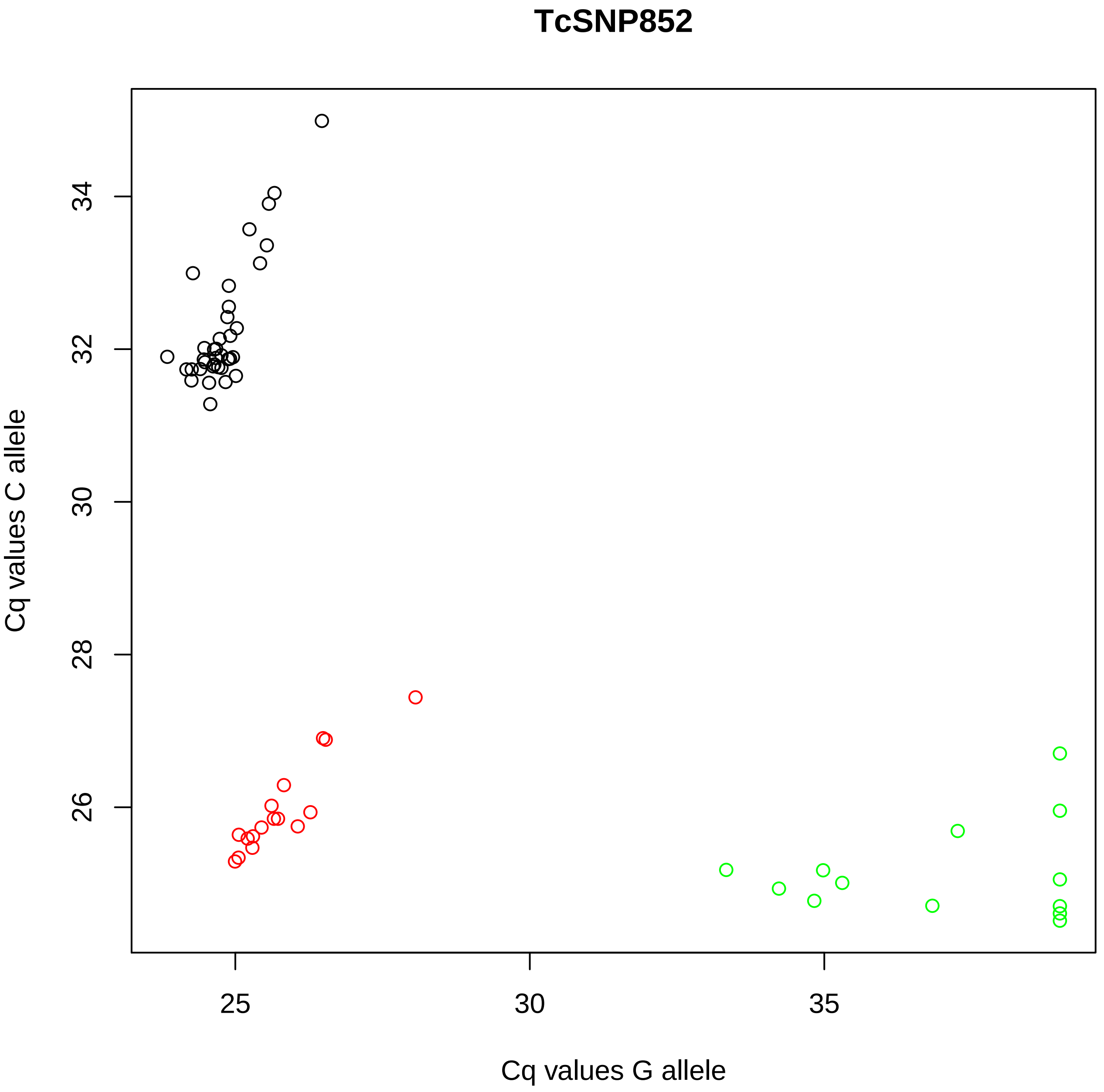
TcSNP852
Cq values C allele
26
28
30
32
34
Cq values G allele
25
30
35

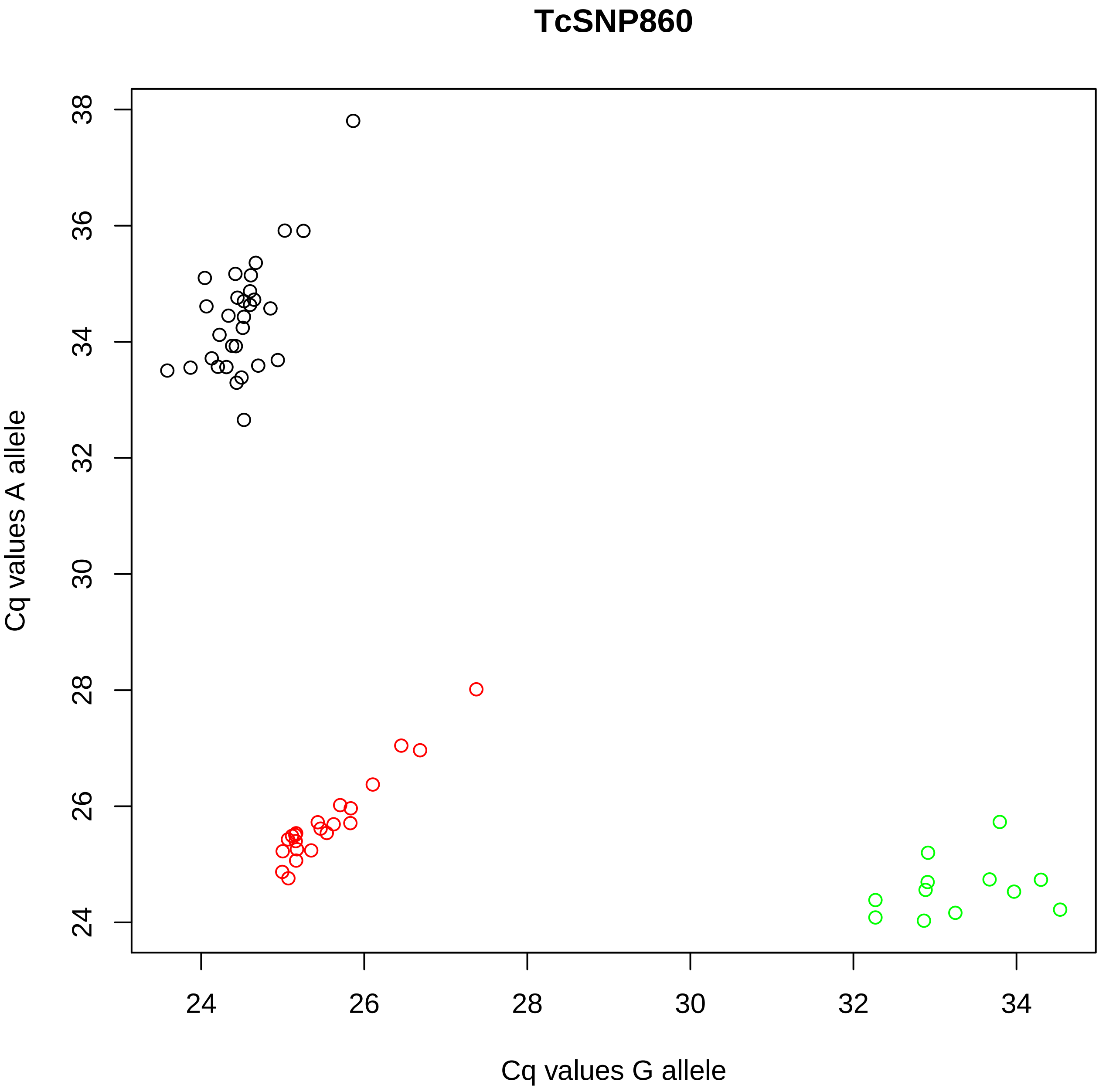
TcSNP860
Cq values A allele
Cq values G allele
24
26
28
30
32
34
36
38
24
26
28
30
32
34

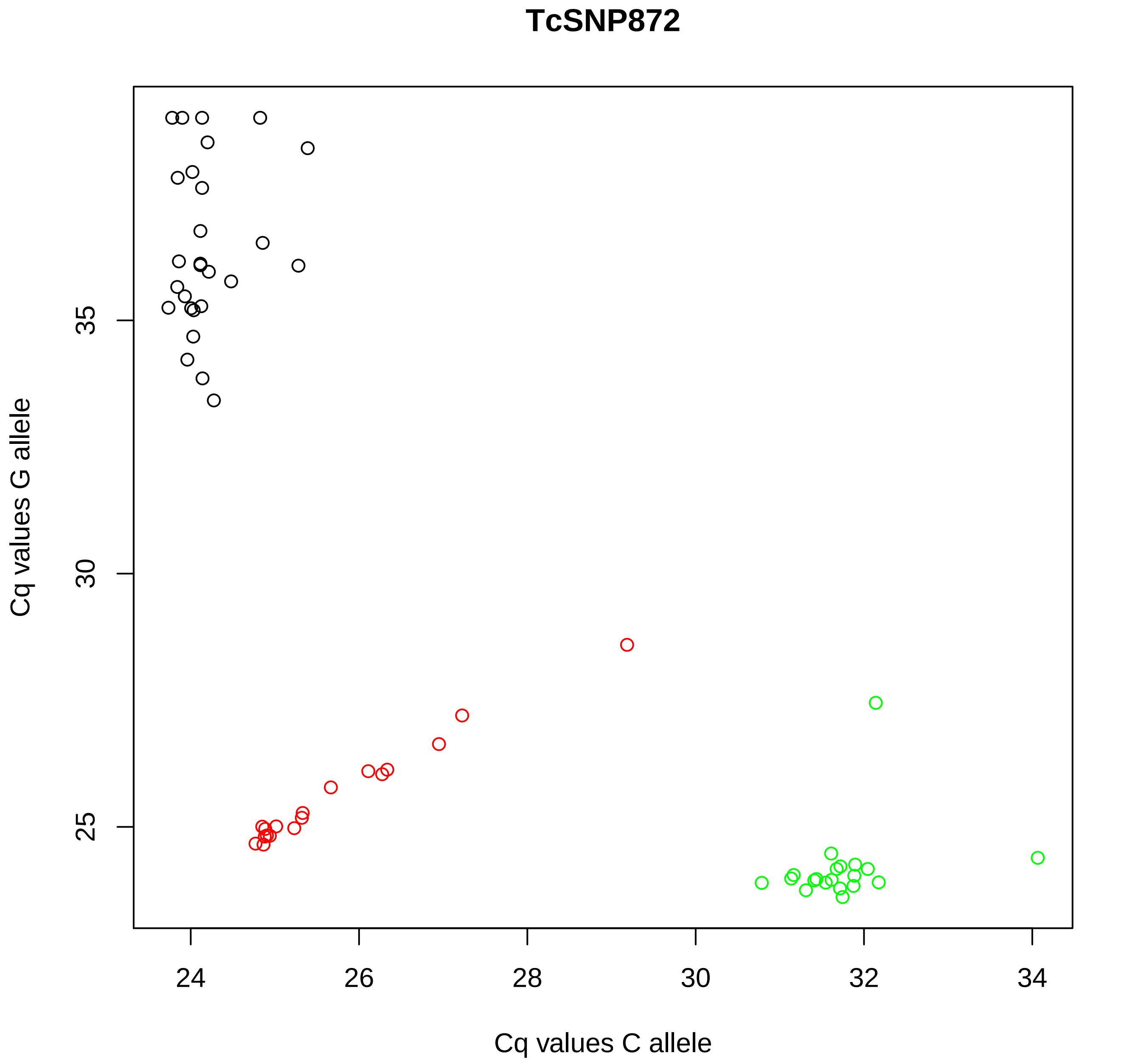
TcSNP872
Cq values G allele
Cq values C allele
25
30
35
24
26
28
30
32
34

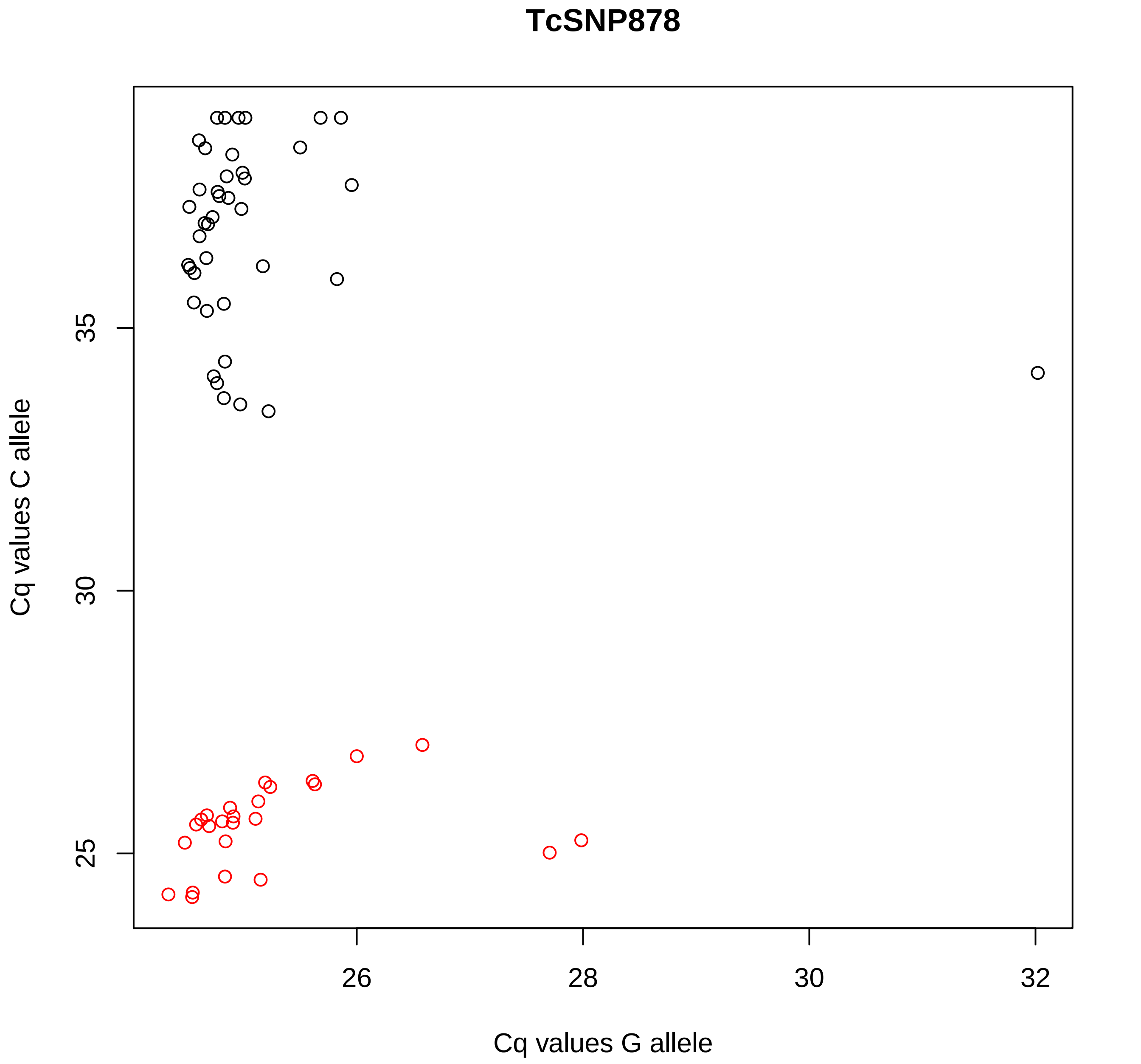
TcSNP878
Cq values C allele
Cq values G allele
25
30
35
26
28
30
32

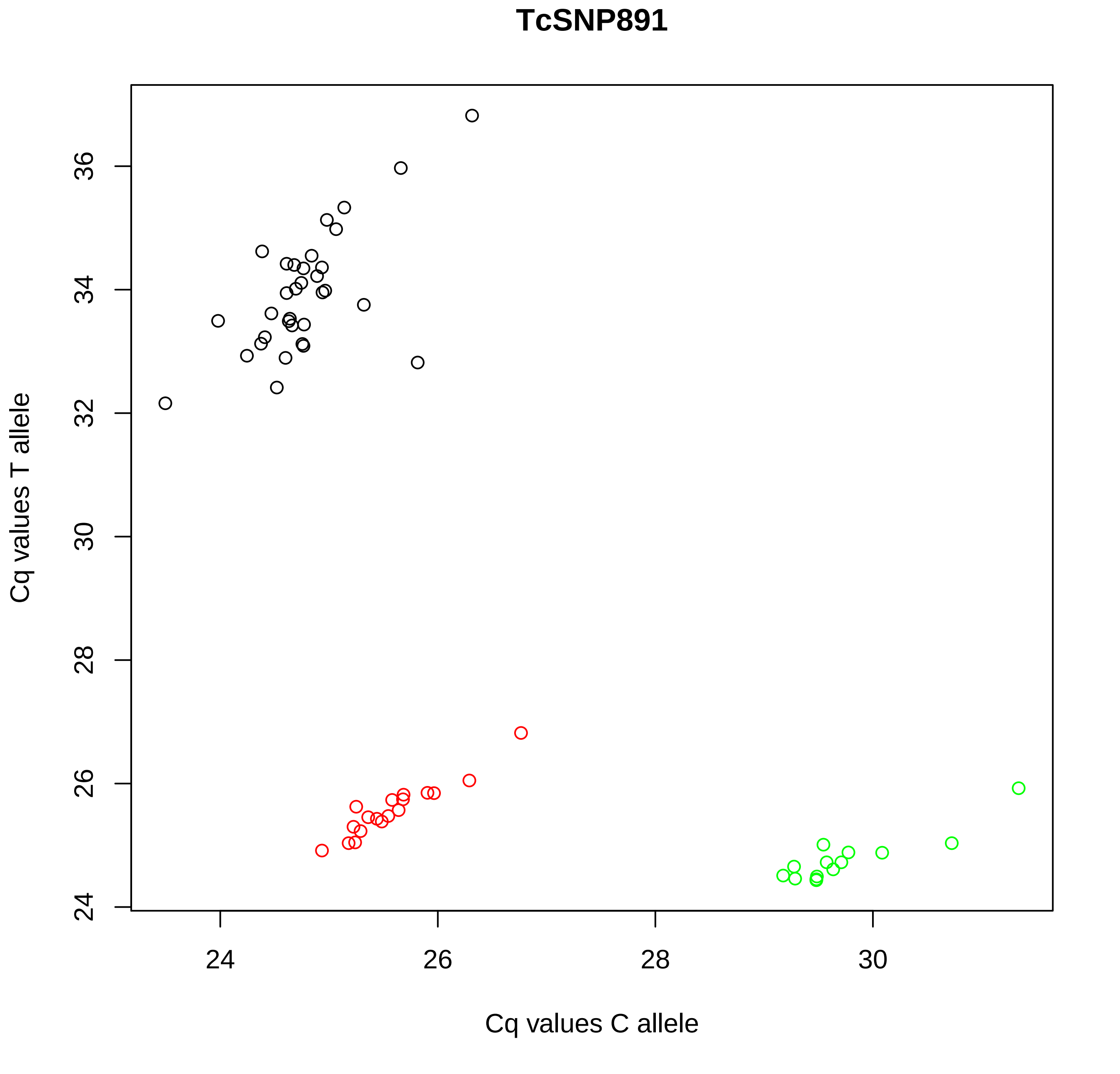
TcSNP891
Cq values T allele
Cq values C allele
24
26
28
30
32
34
36
24
26
28
30

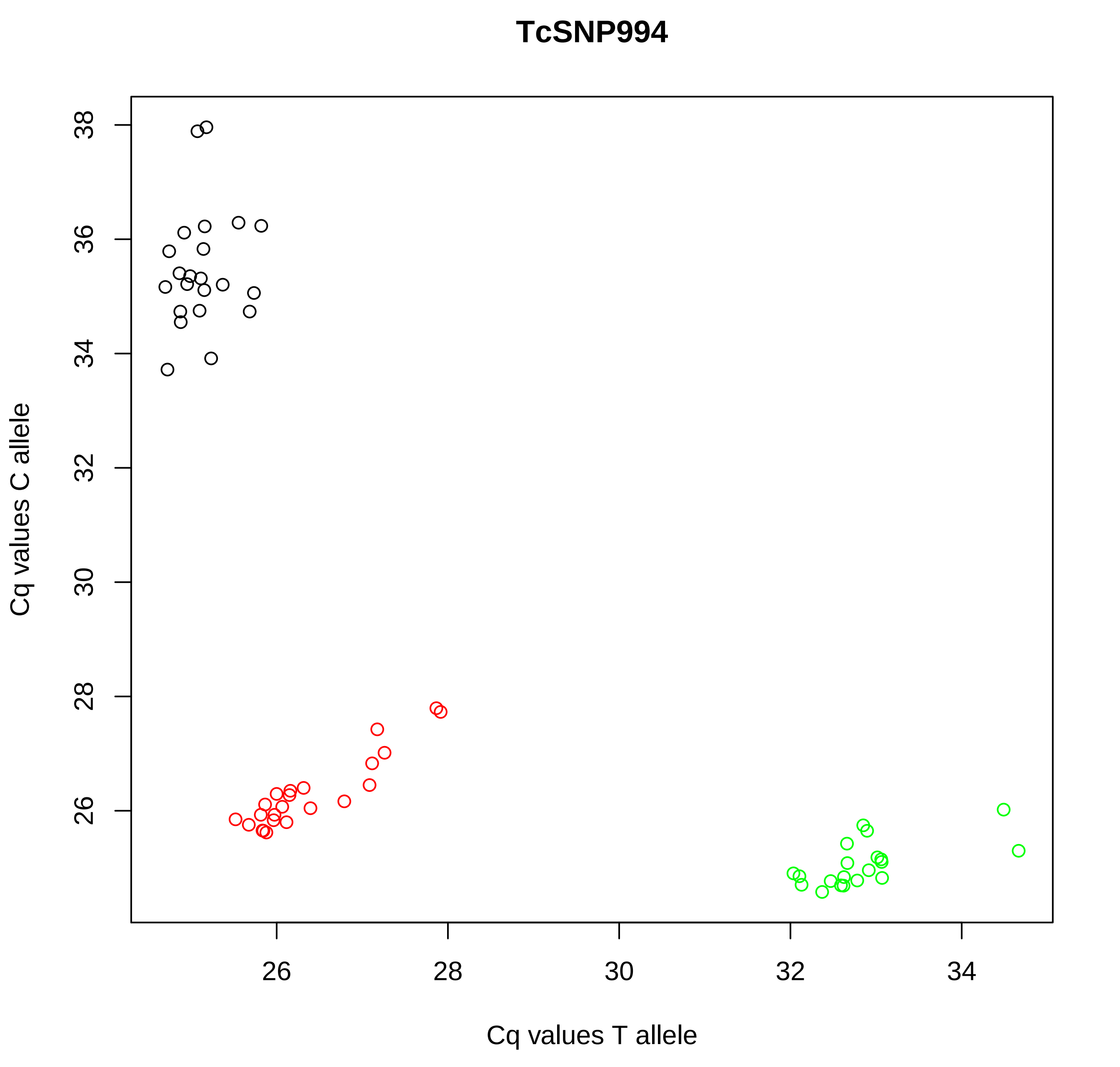
TcSNP994
Cq values C allele
Cq values T allele
38
36
34
32
30
28
26
26
28
30
32
34

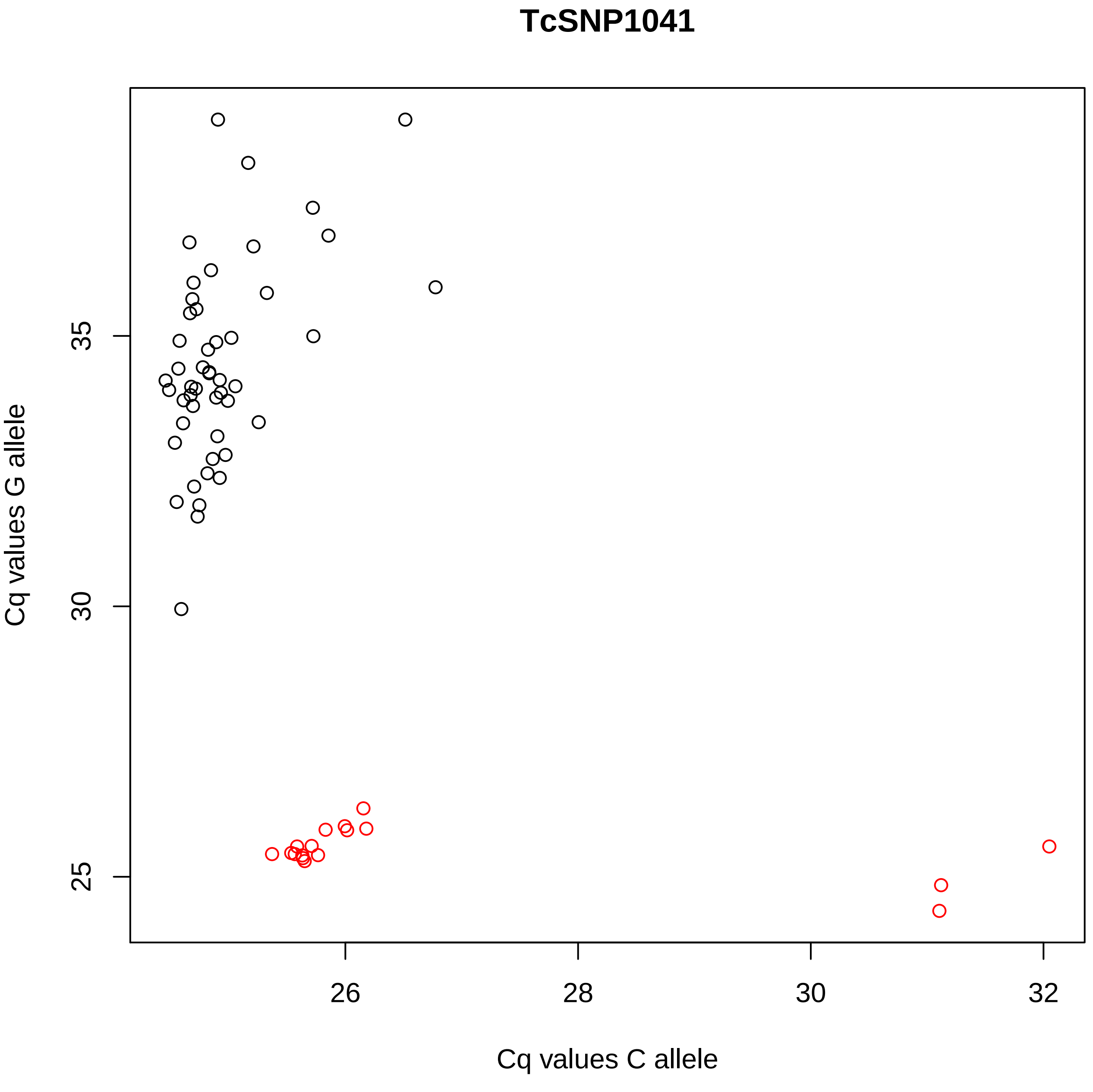
TcSNP1041
Cq values G allele
Cq values C allele
25
30
35
26
28
30
32

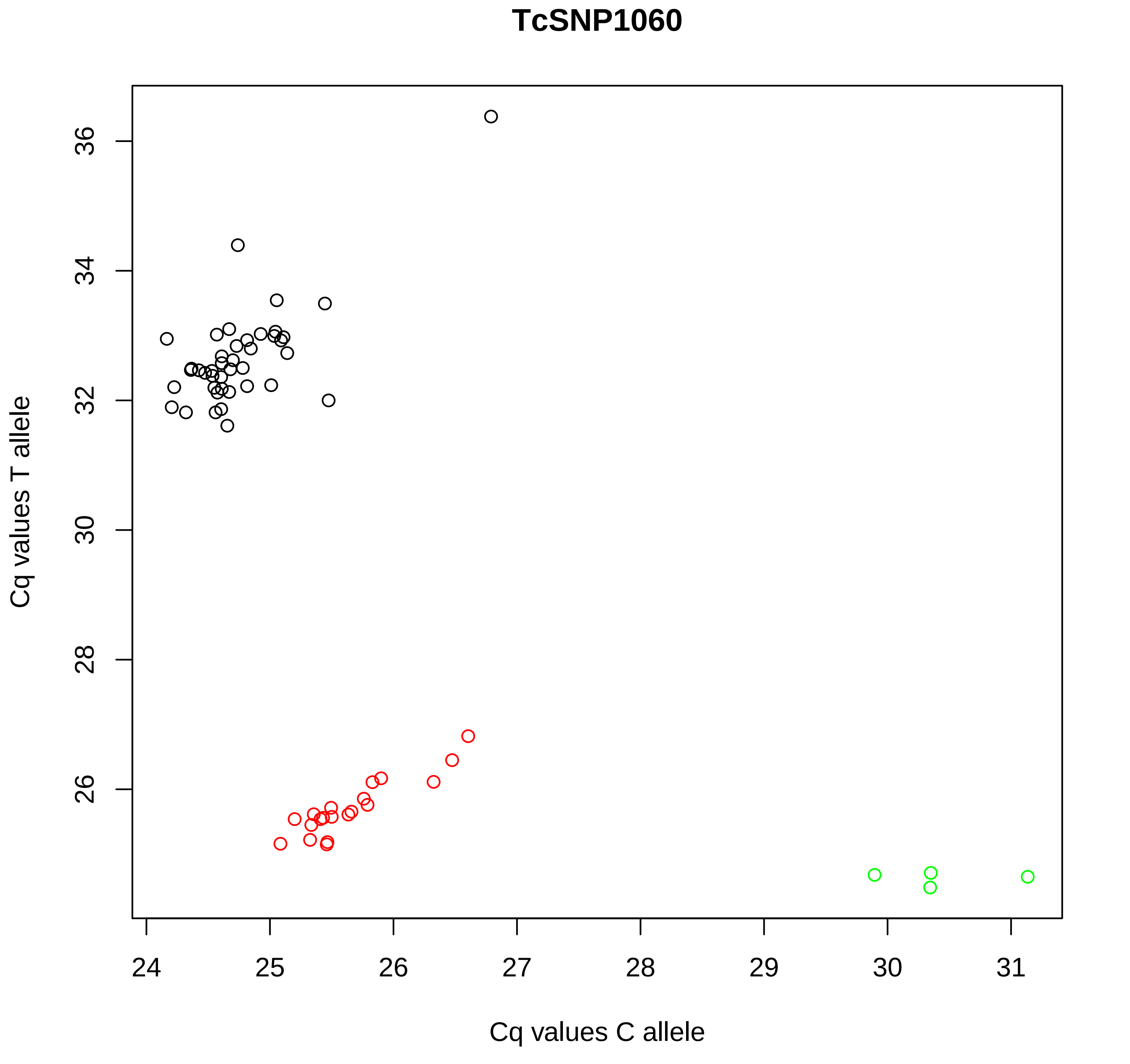
TcSNP1060
Cq values T allele
Cq values C allele
26
28
30
32
34
36
24
25
26
27
28
29
30
31

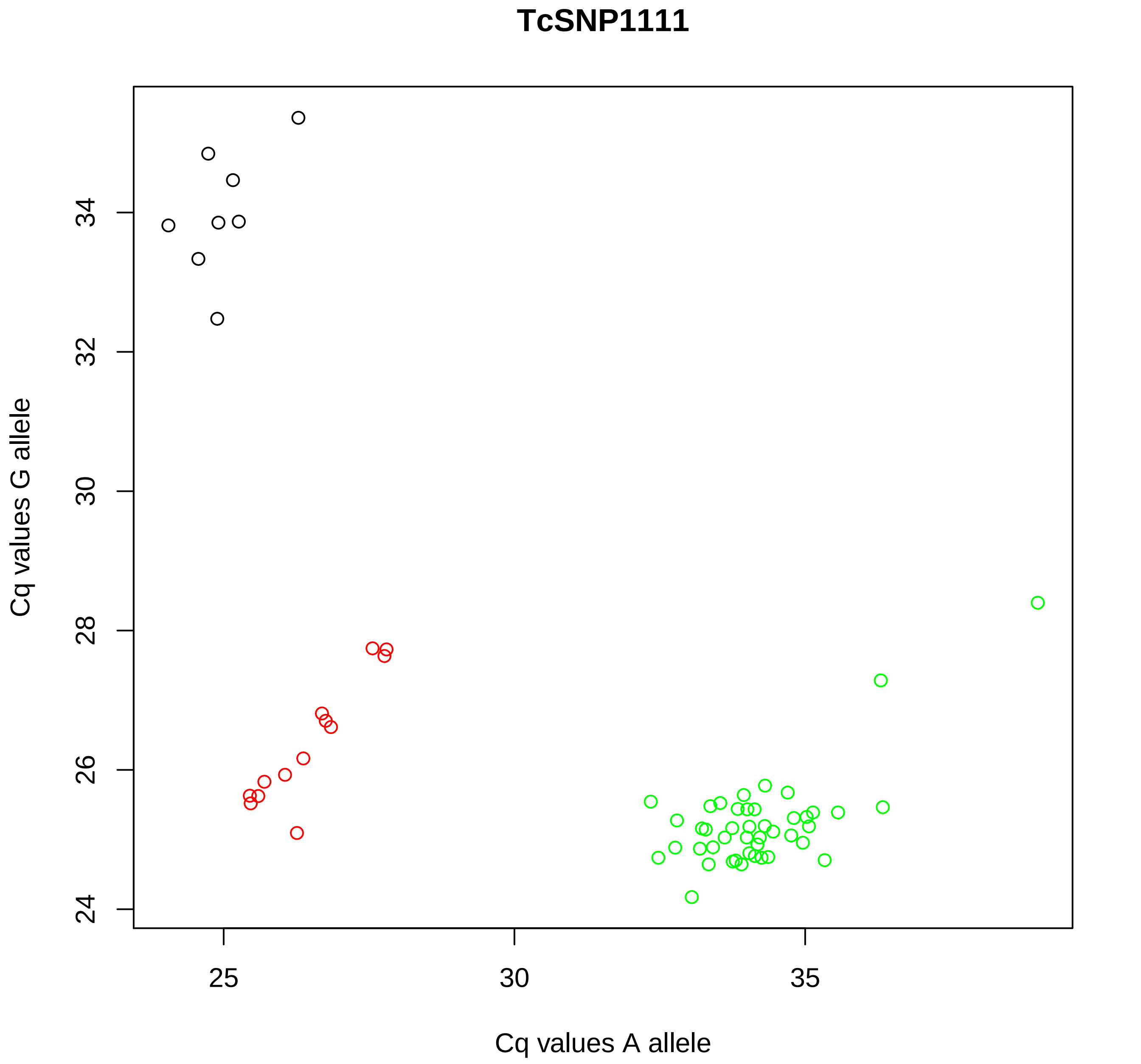
TcSNP1111
Cq values G allele
24
26
28
30
32
34
25
30
35
Cq values A allele

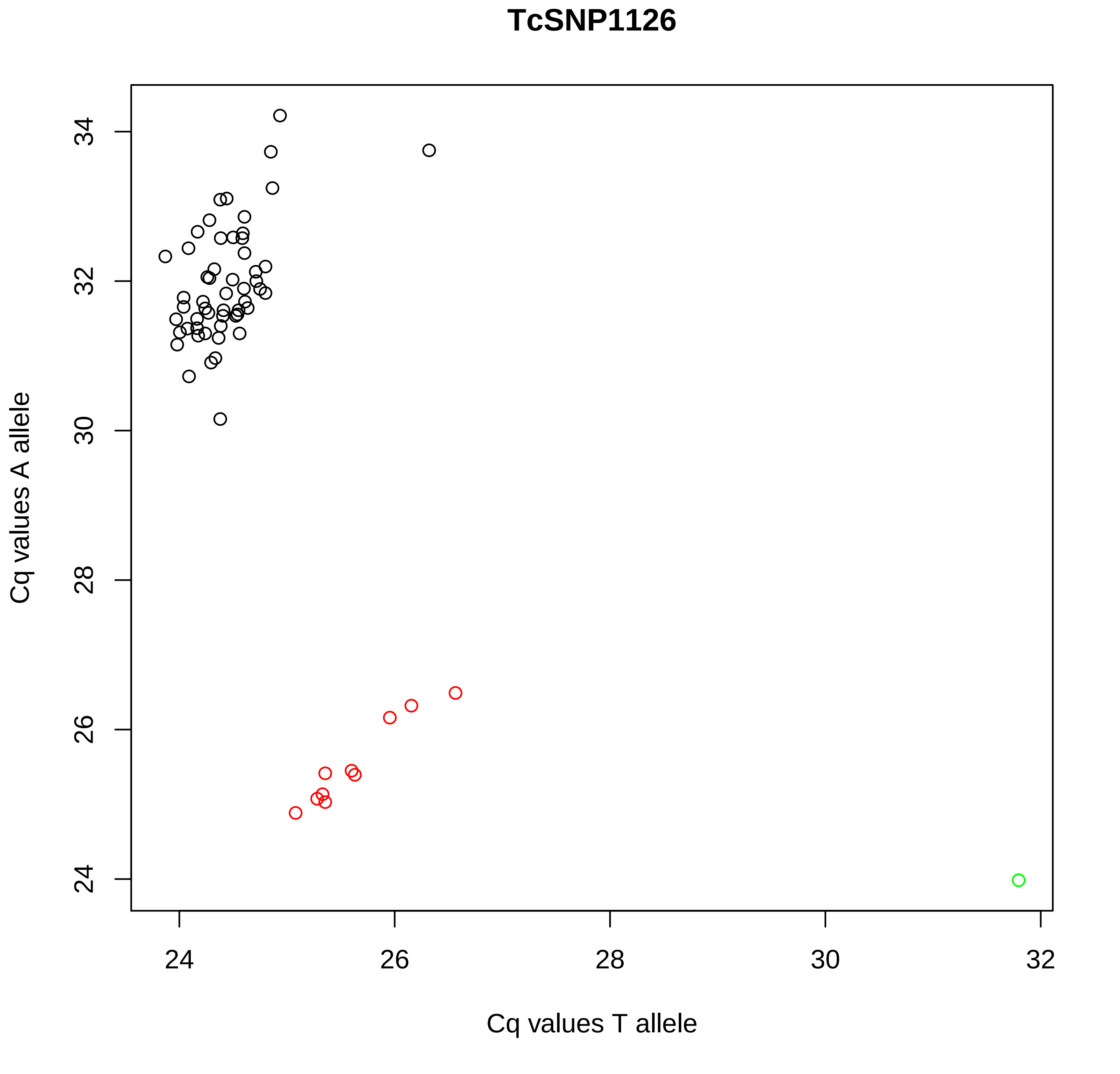
TcSNP1126
Cq values A allele
Cq values T allele
24
26
28
30
32
34
24
26
28
30
32

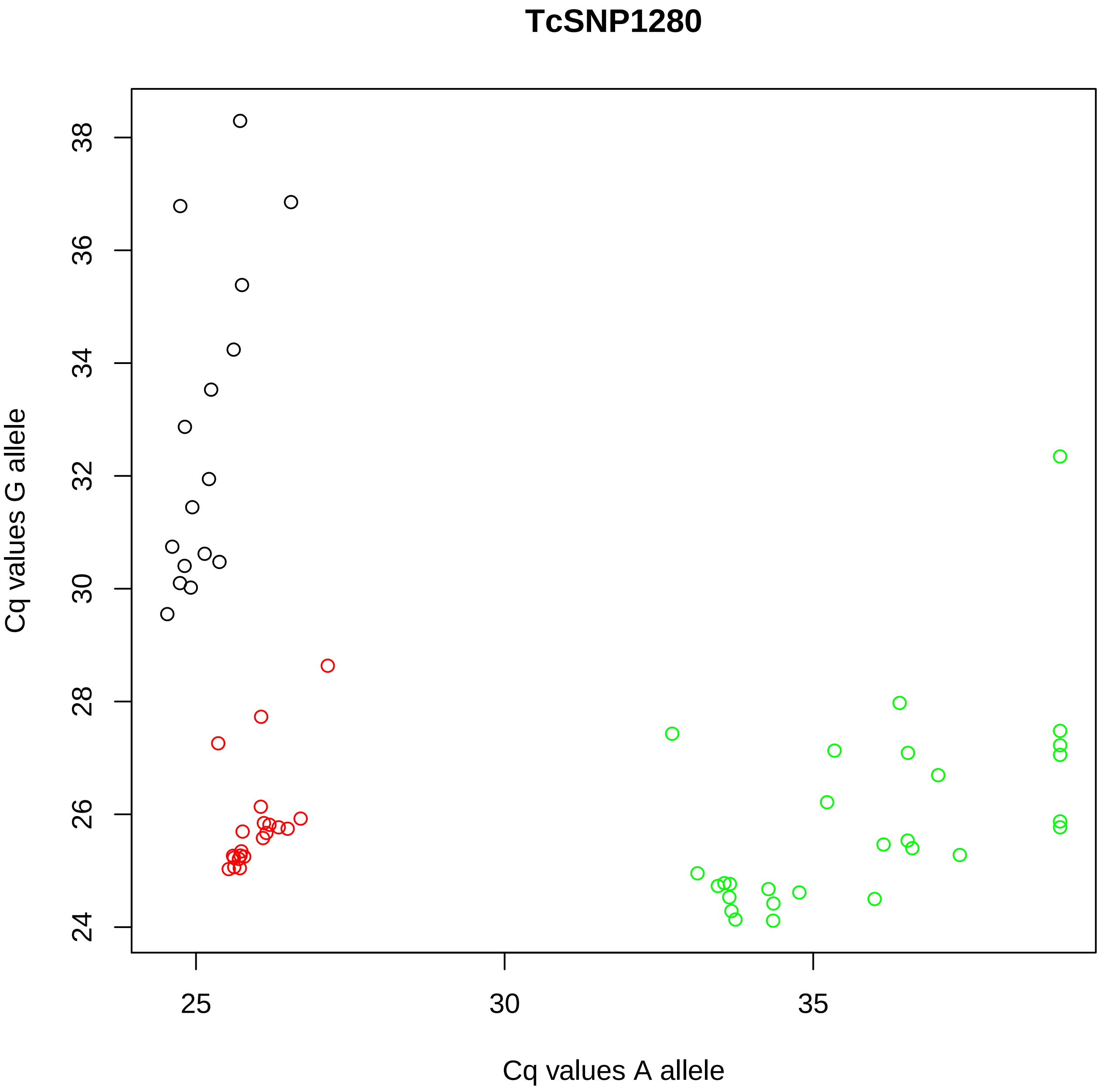
TcSNP1280
Cq values G allele
Cq values A allele
24
26
28
30
32
34
36
38
25
30
35

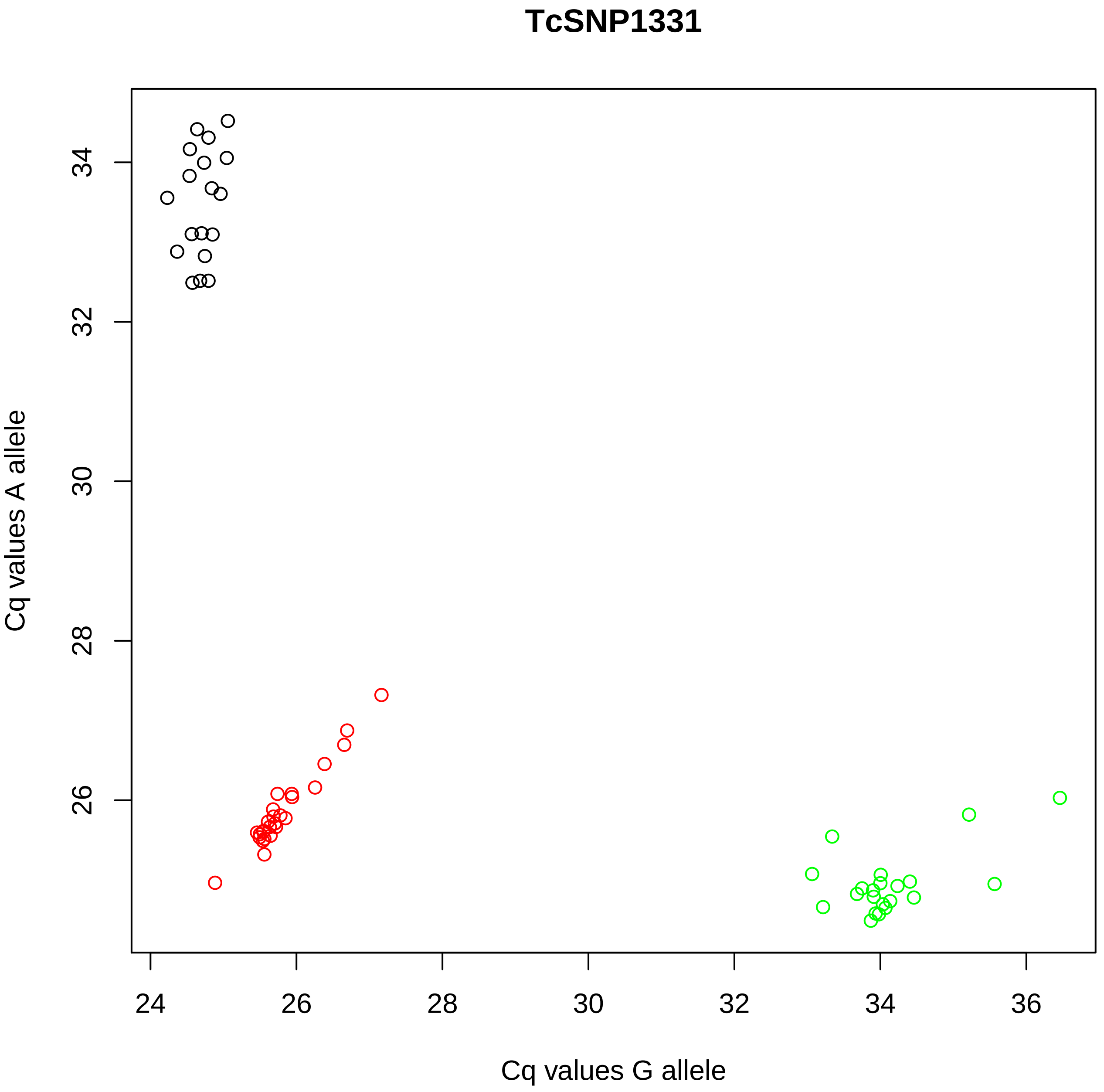
TcSNP1331
Cq values A allele
Cq values G allele
24
26
28
30
32
34
36
26
28
30
32
34

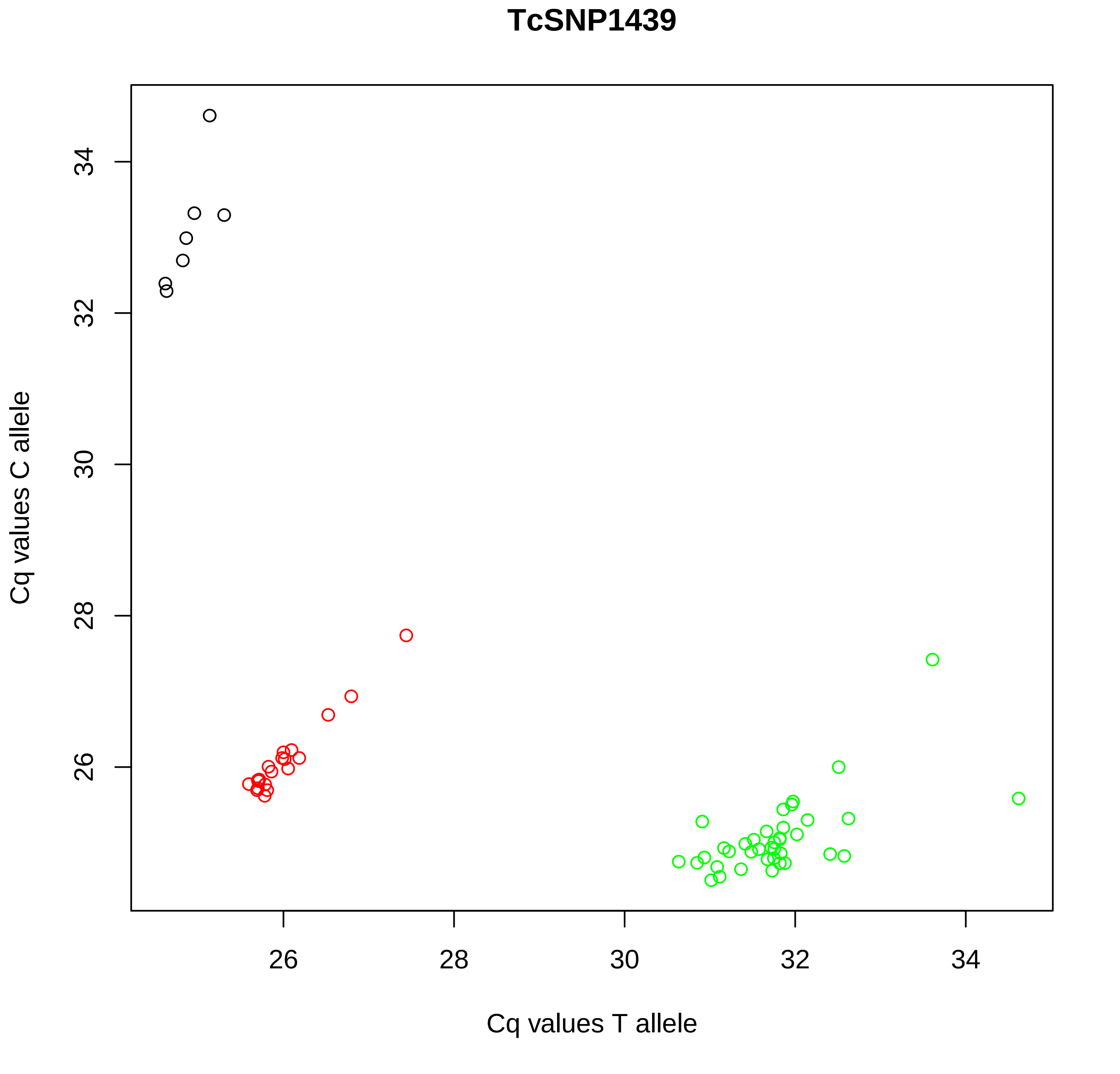
TcSNP1439
Cq values C allele
Cq values T allele
26
28
30
32
34

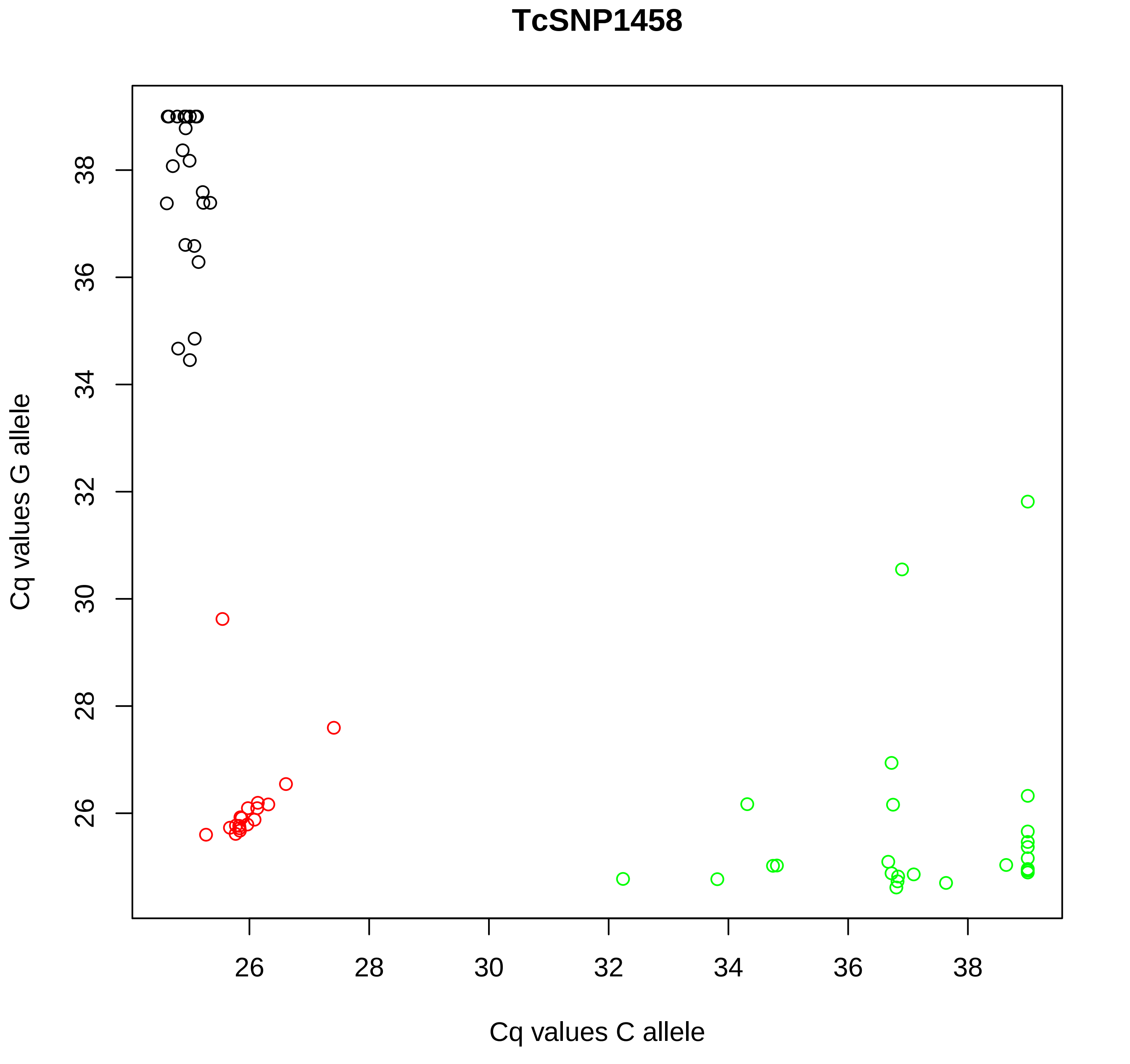
TcSNP1458
Cq values G allele
26
28
30
32
34
36
38
Cq values C allele
26
28
30
32
34
36
38

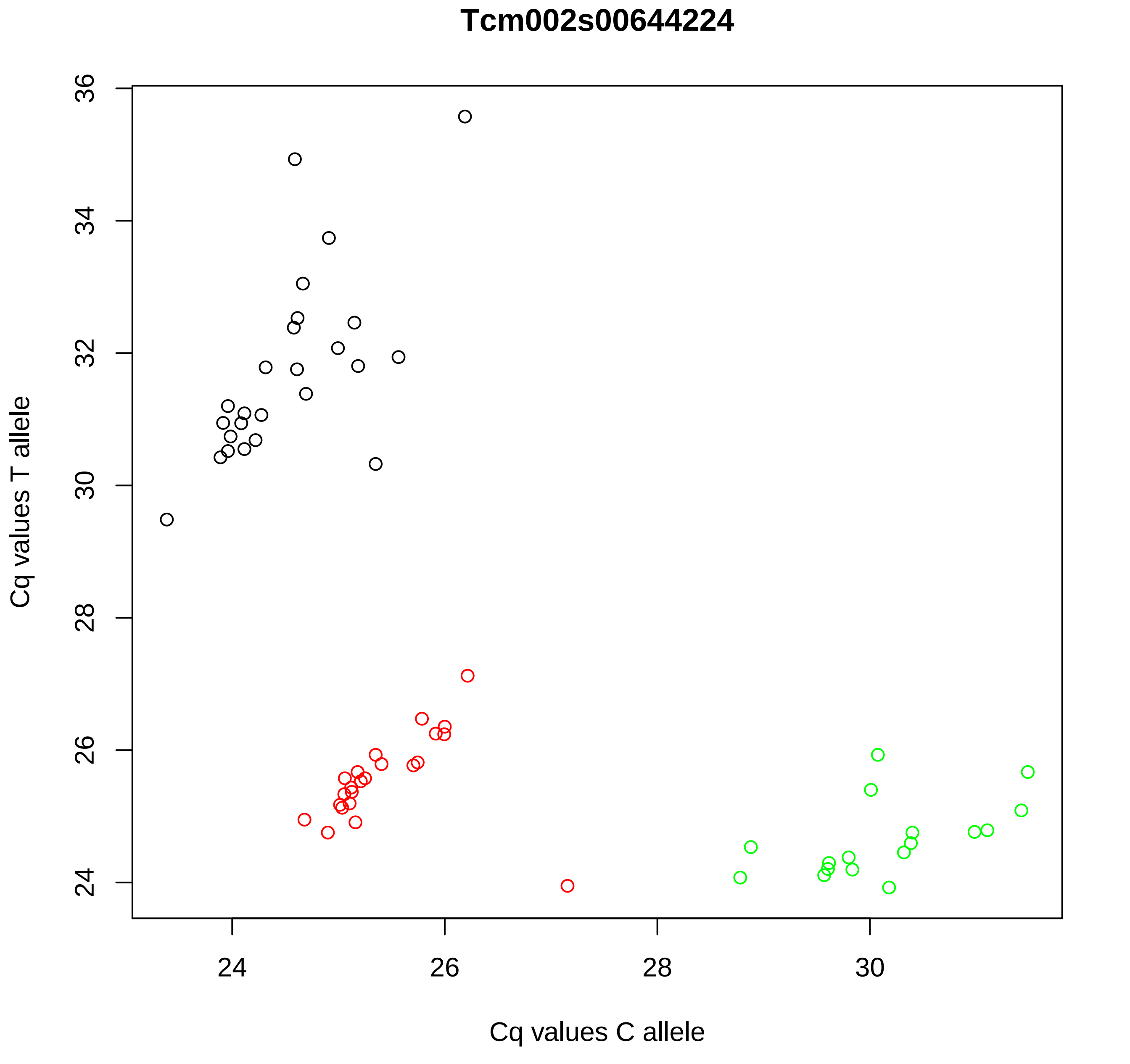
Tcm002s00644224
Cq values T allele
36
34
32
30
28
26
24
Cq values C allele
24
26
28
30

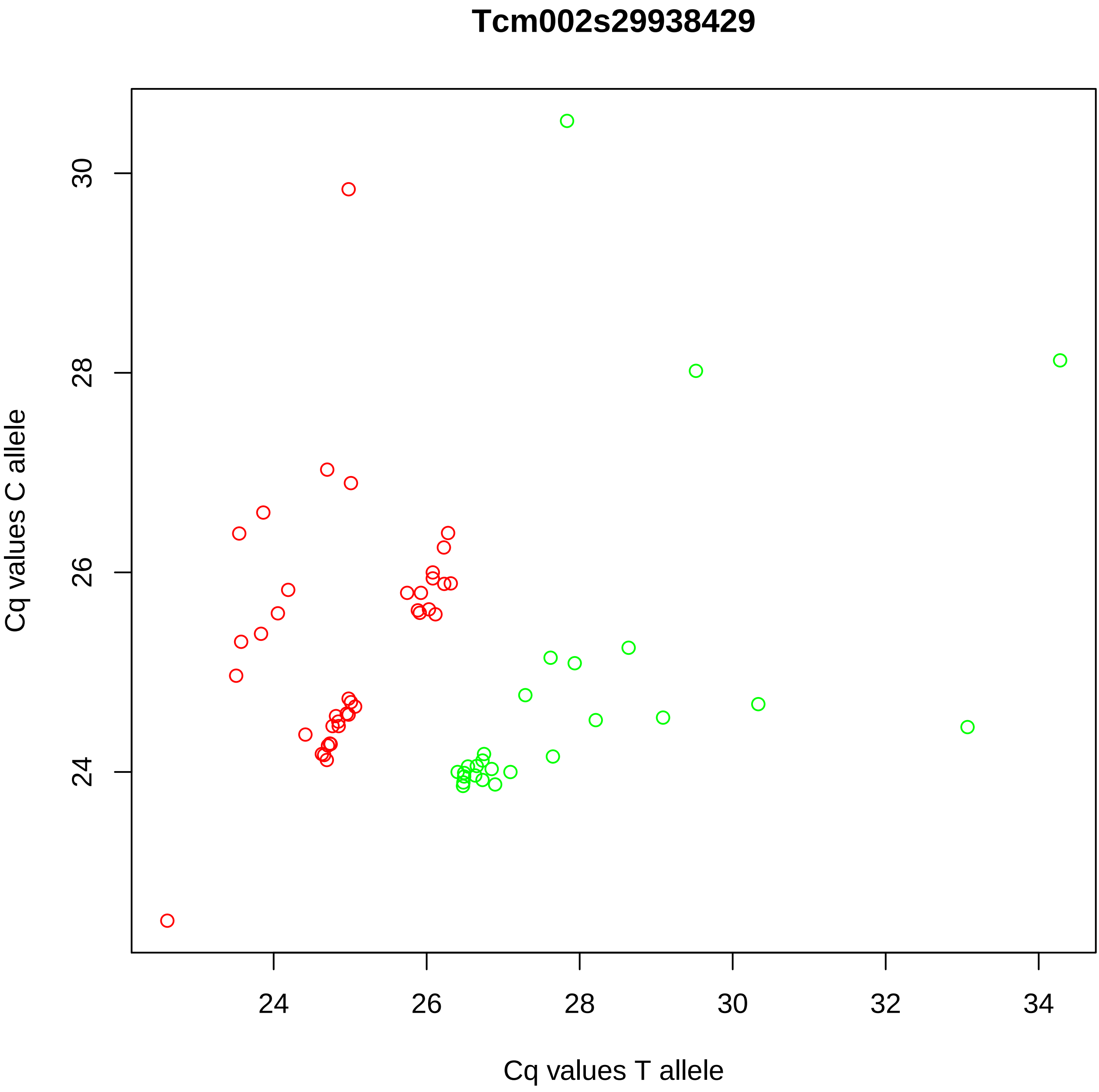
Tcm002s29938429
Cq values C allele
Cq values T allele
24
26
28
30
32
34
24
26
28
30

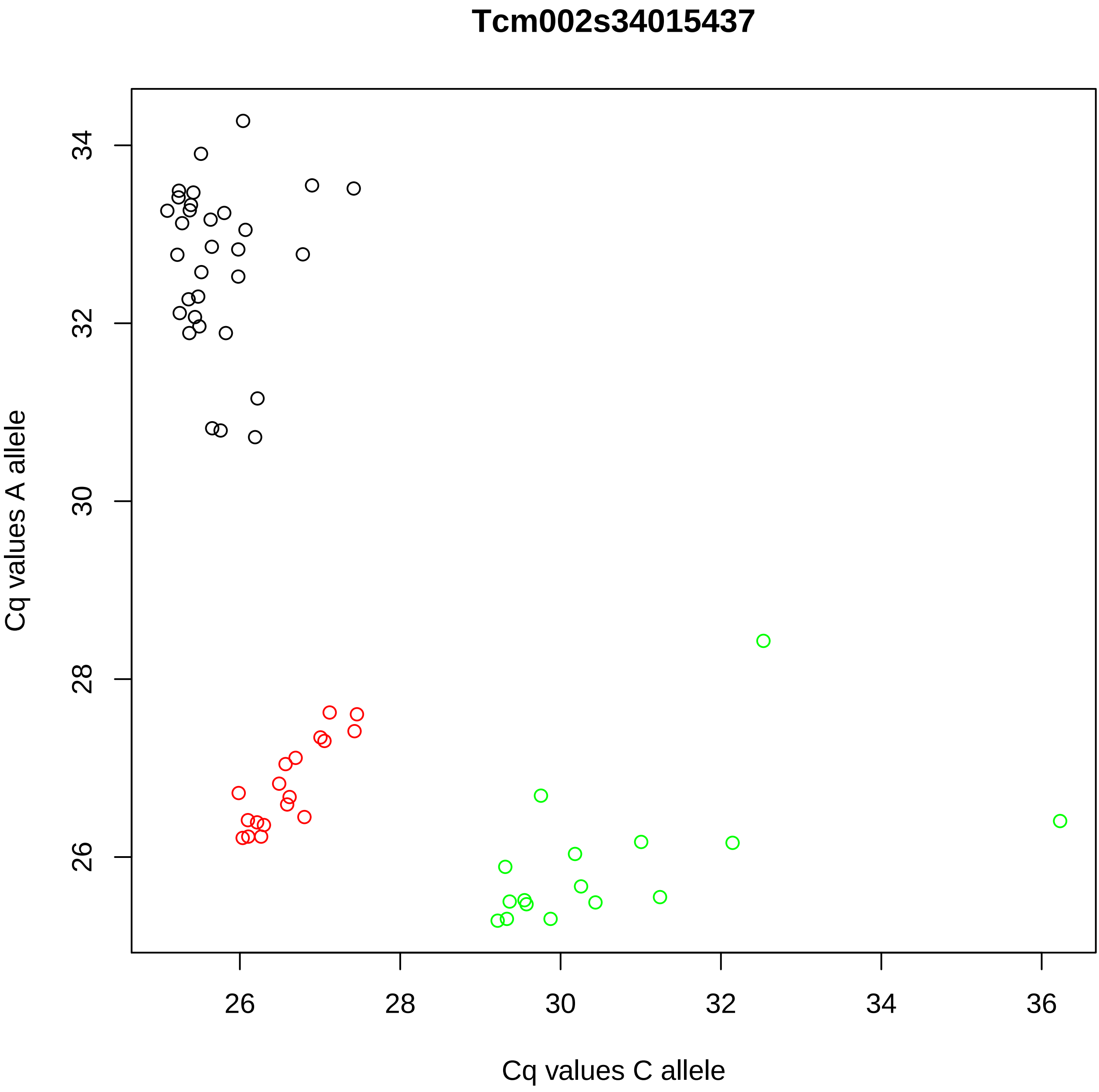

Tcm002s34015437
Cq values A allele
Cq values C allele
26
28
30
32
34
36
26
28
30
32
34

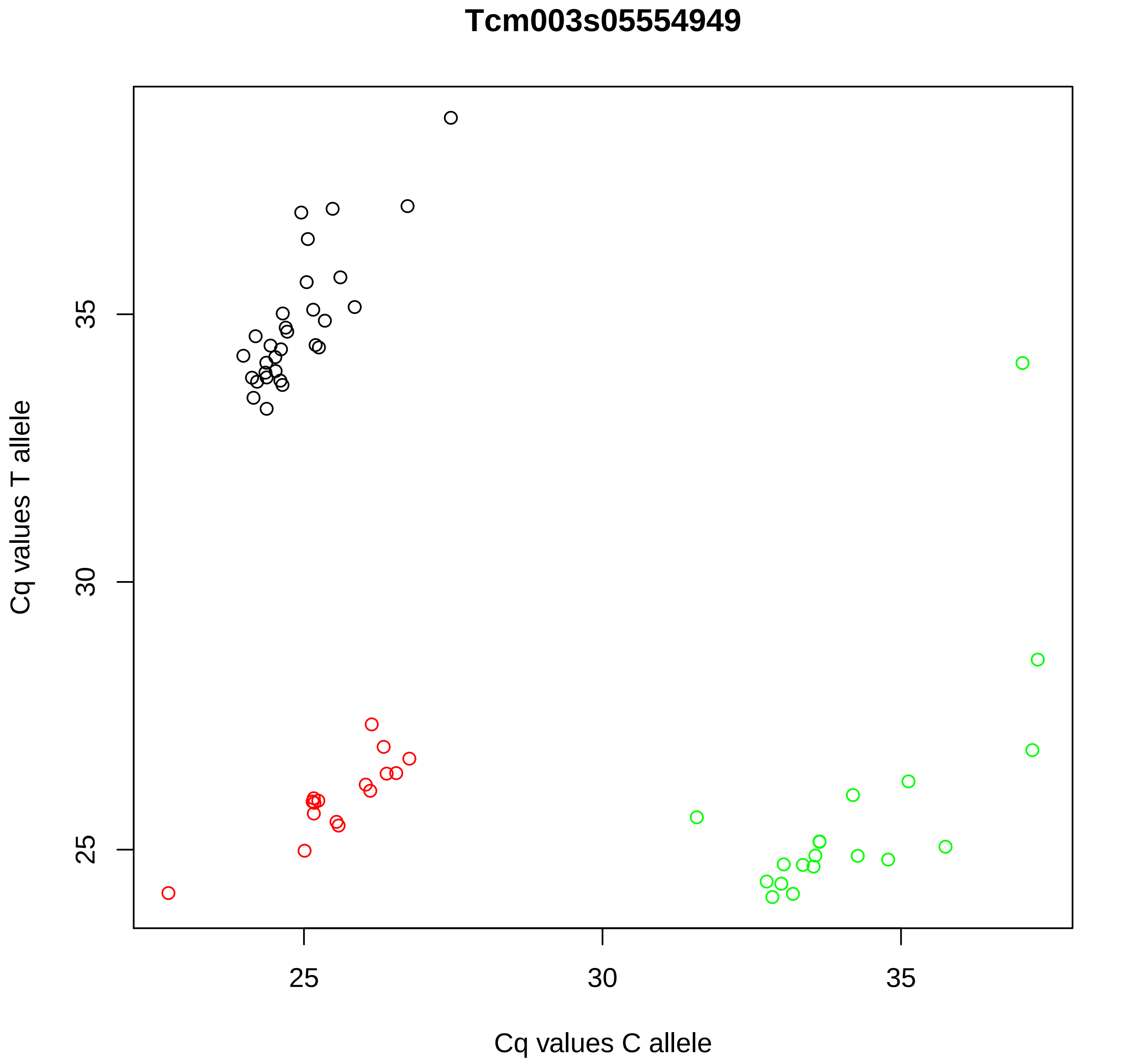
Tcm003s05554949
Cq values T allele
Cq values C allele
25
30
35
25
30
35

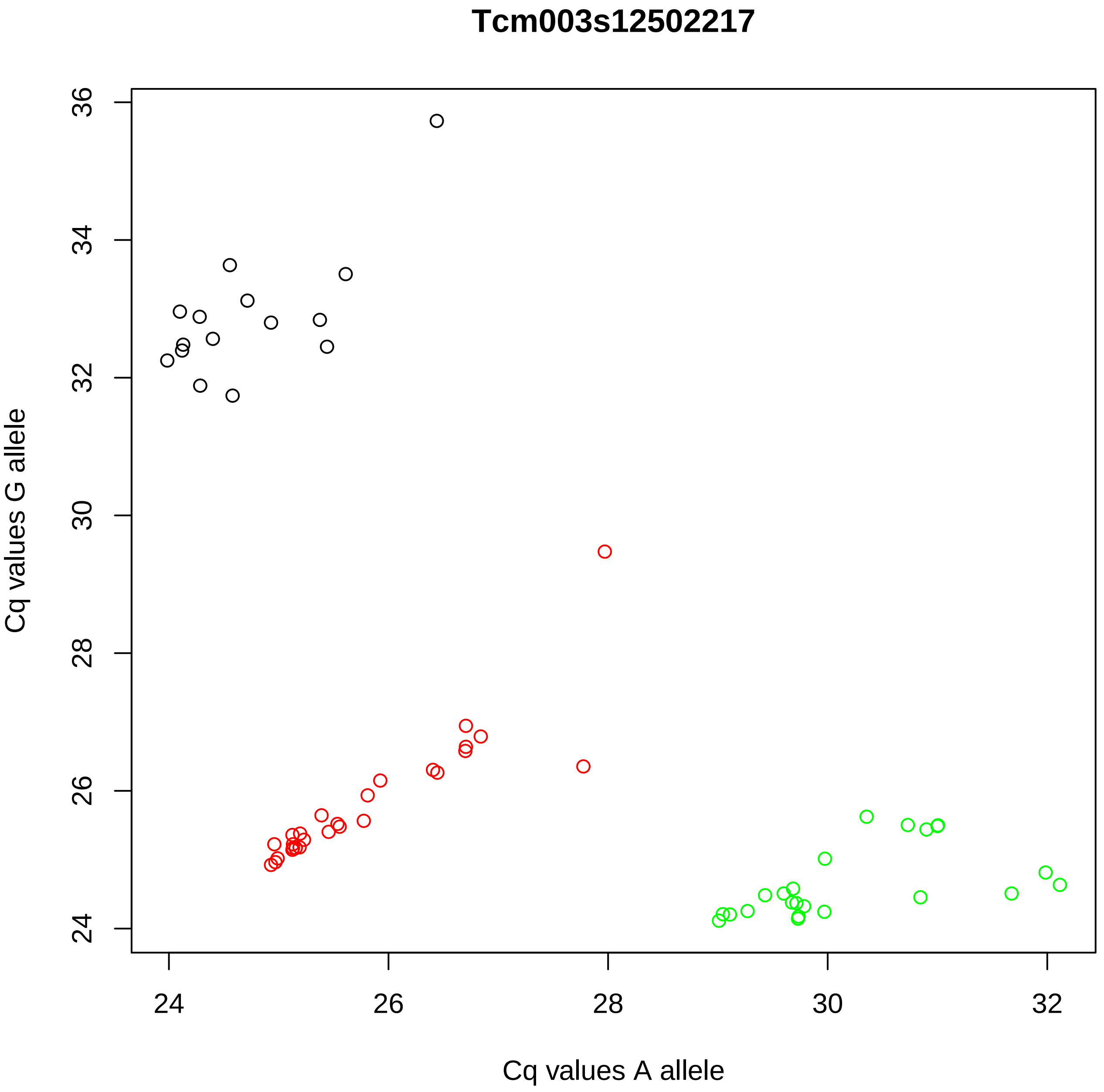
Tcm003s12502217
Cq values G allele
Cq values A allele
24
26
28
30
32
34
36

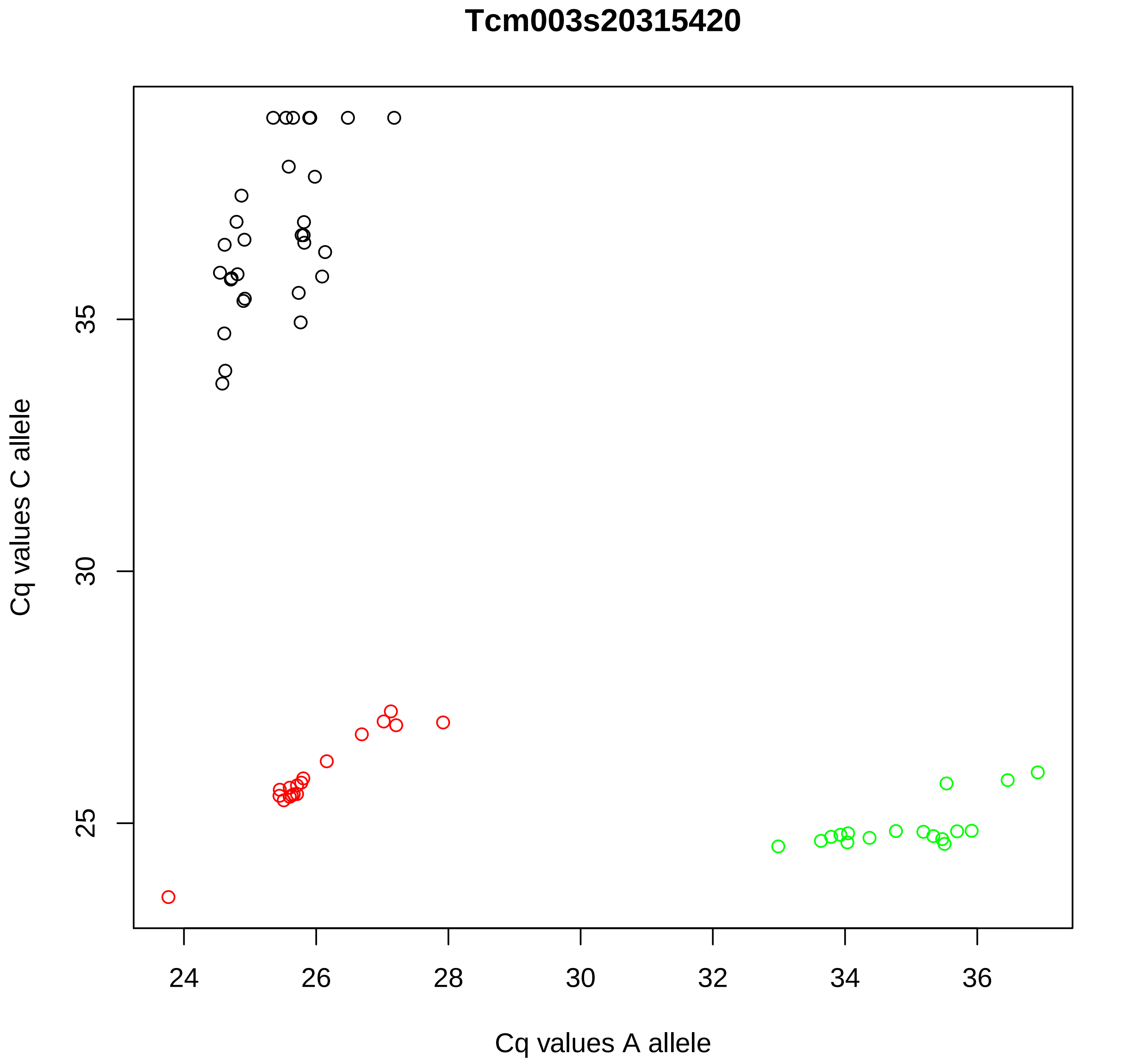

Tcm003s20315420
Cq values C allele
Cq values A allele
25
30
35
24
26
28
30
32
34
36

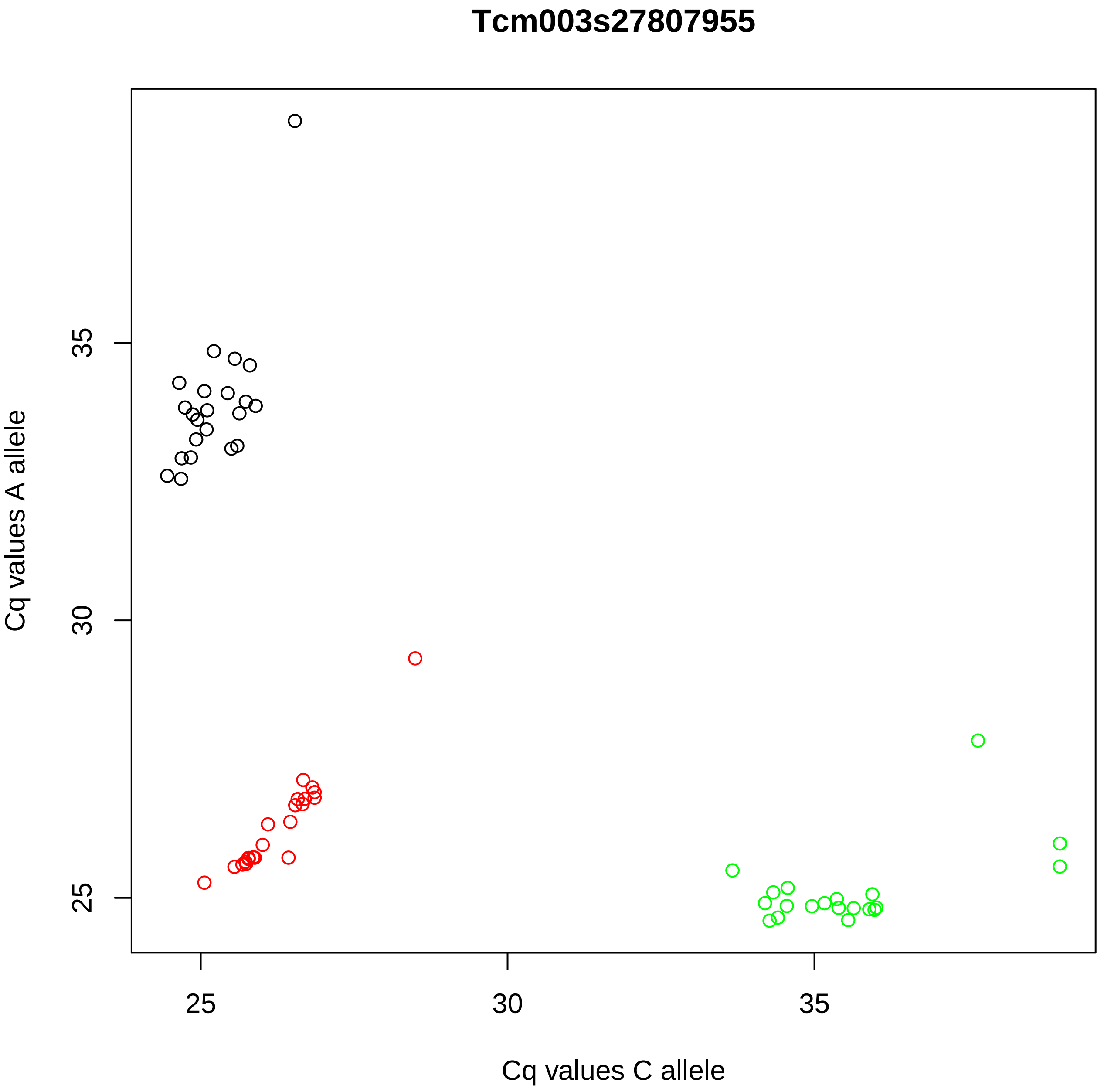

Tcm003s27807955
Cq values A allele
Cq values C allele
25
30
35
25
30
35

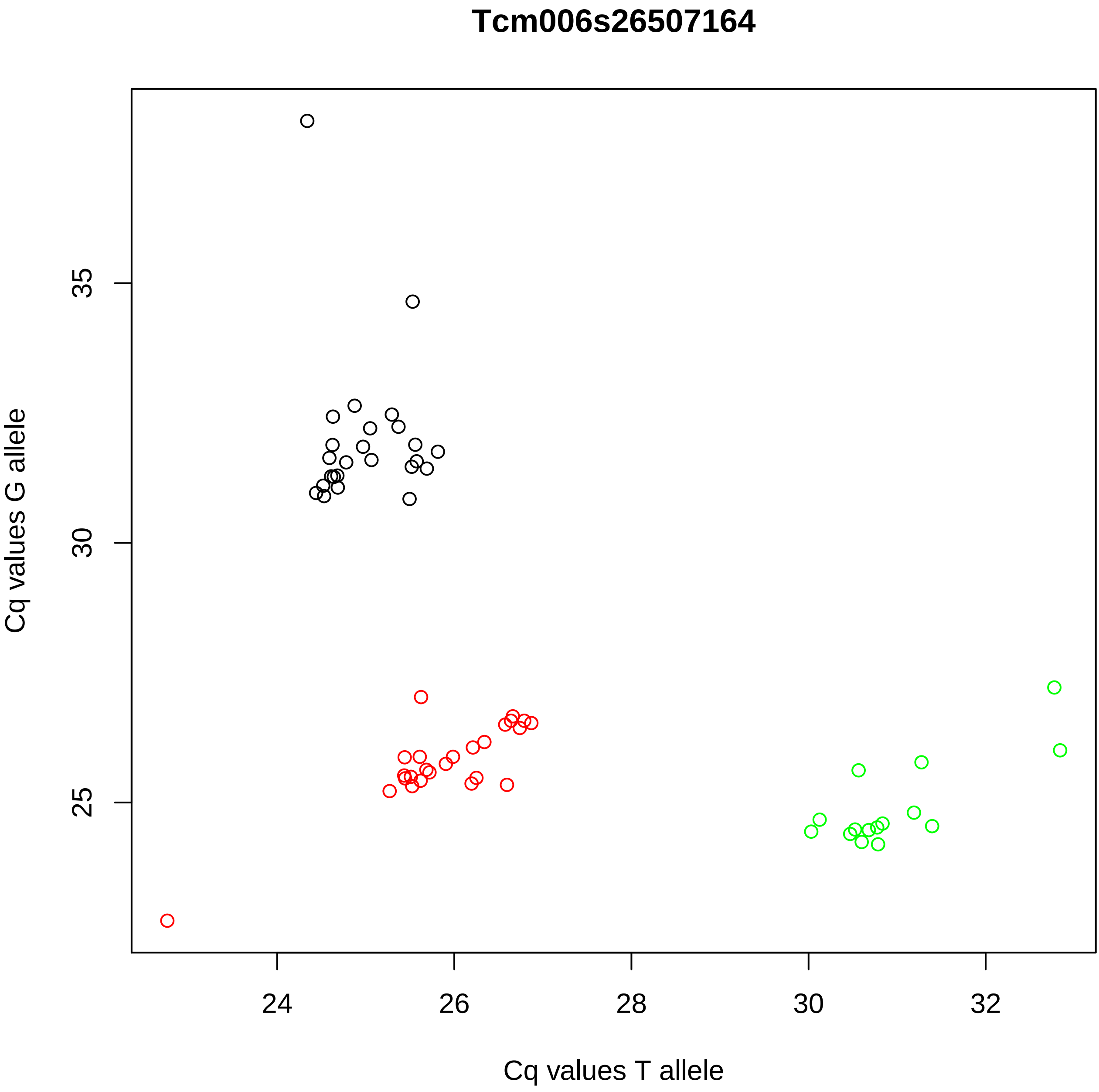
Tcm006s26507164
Cq values G allele
Cq values T allele
25
30
35
24
26
28
30
32

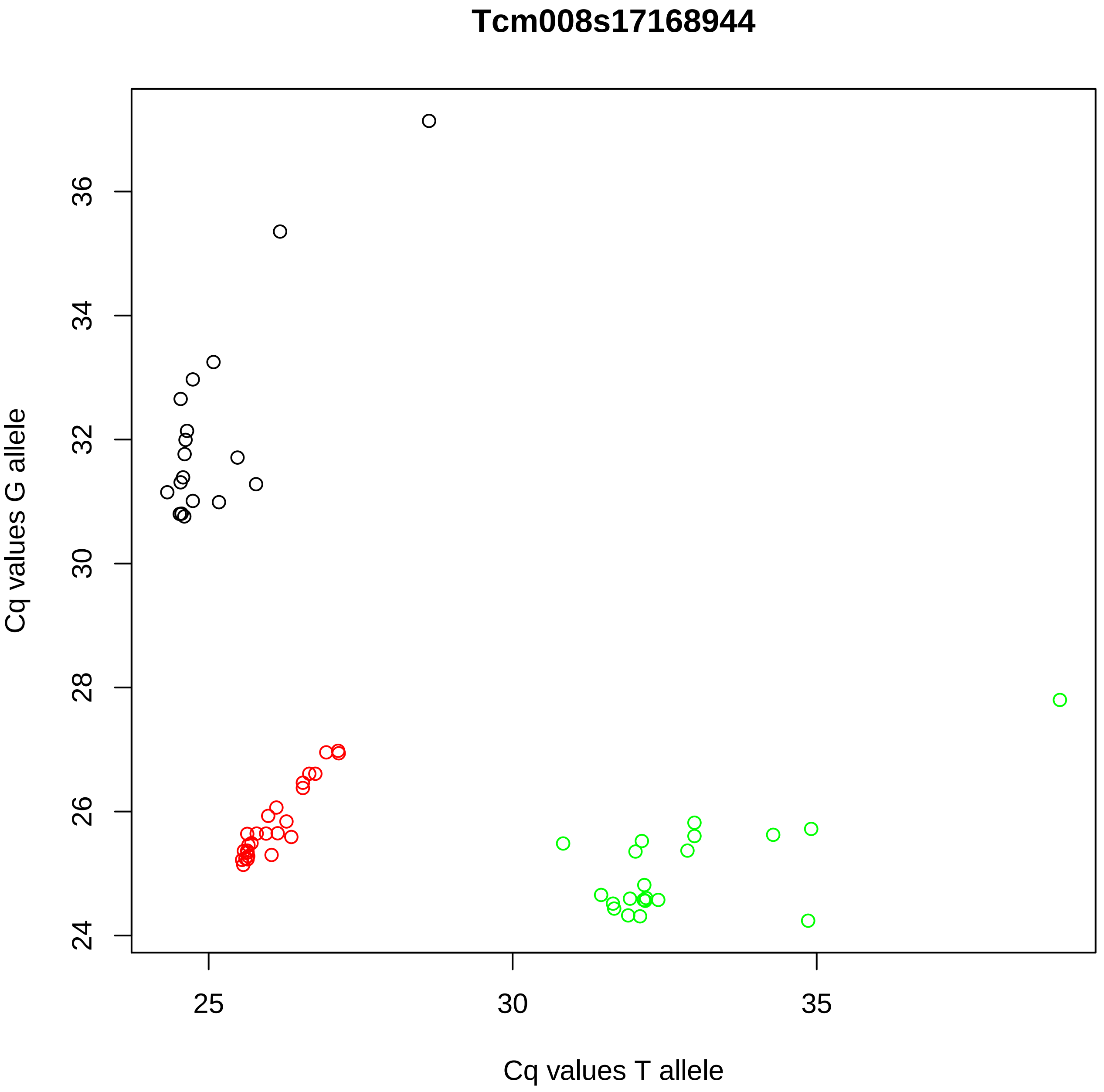
Tcm008s17168944
Cq values G allele
Cq values T allele
24
26
28
30
32
34
36
25
30
35

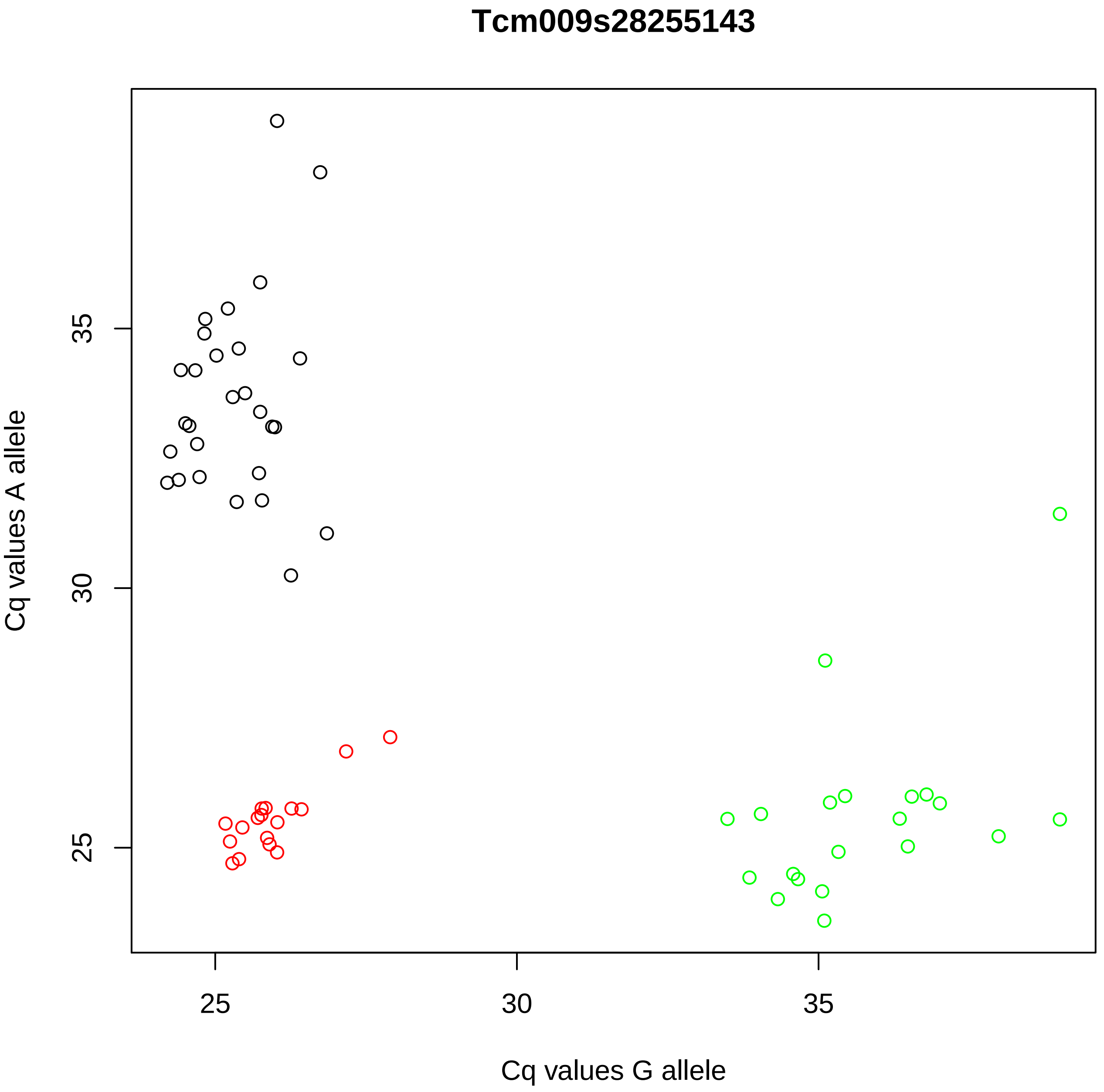

Tcm009s28255143
Cq values A allele
Cq values G allele
25
30
35
25
30
35

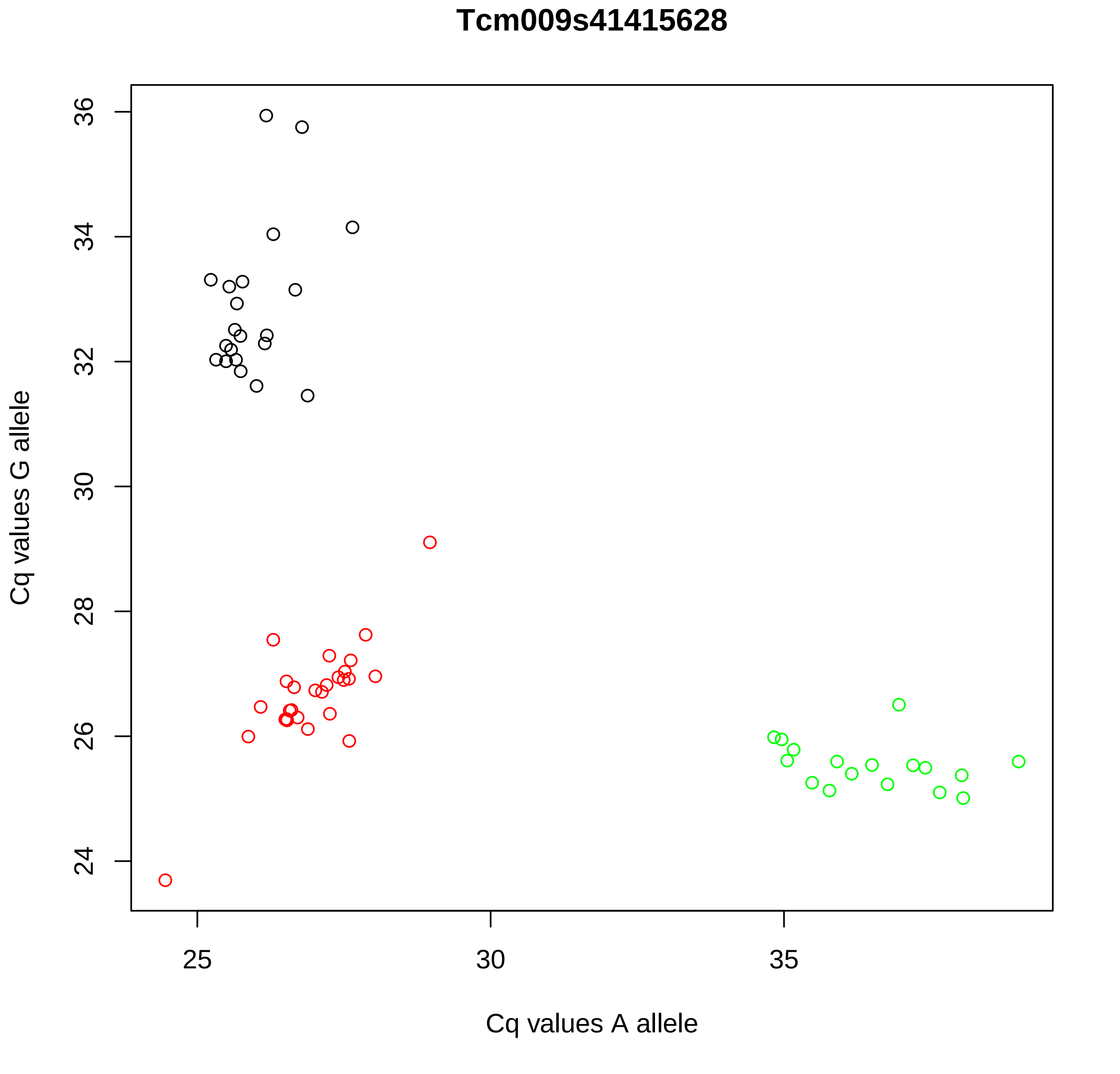
Tcm009s41415628
Cq values G allele
Cq values A allele
24
26
28
30
32
34
36
25
30
35
