# Supplementary material for: The development of a novel SNP genotyping assay to differentiate cacao clones
Source: Sci Rep. 2019 Jul 2;9:9512. doi: 10.1038/s41598-019-45884-8 (PMC6606624; doi:10.1038/s41598-019-45884-8)
Supplement: Supplementary file 2 — Supplementary Dataset 2 [file 41598_2019_45884_MOESM2_ESM.pdf]

The development of a novel SNP genotyping assay to differentiate cacao clones

Jocelyn De Wever, Helena Everaert, Frauke Coppieters, Hayley Rottiers, Koen Dewettinck, Steve Lefever, Kathy Messens

Appendix 2: DMAS-qPCR Difference plots

One difference plot per SNP assay

Two dimensional plot created by plotting the ΔCq (Cq_{ref} –Cq_{alt}) against the clones analyzed. In these plots heterozygous samples will show Cq differences around zero, while homozygous samples show either negative or positive differences. The plots below display the results of each DMAS-qPCR SNP assay used for genotyping different cacao accessions.

X/ Y values

X-axis : cacao accessions analyzed

**1**	LCTEEN37/A
**2**	IFC5[CIV]
**3**	PA137[PER]
**4**	ICS1
**5**	ICS43
**6**	APA4
**7**	MA12[BRA]
**8**	LCTEEN62/S-4
**9**	POUND16/B
**10**	MAN15/2[BRA]
**11**	IMC67
**12**	PA88[PER]
**13**	PA156[PER]
**14**	PA70[PER]
**15**	PA120[PER]
**16**	SCA6
**17**	SCA9
**18**	NA32
**19**	NA33
**20**	MO81
**21**	IMC53
**22**	AMAZ15/15
**23**	PA127
**24**	NA149
**25**	SIAL339
**26**	SPEC41/6-18
**27**	FSC13
**28**	LCTEEN302
**29**	GU114/P
**30**	KER6
**31**	KER3
**32**	GU133/C

**33**	GU261/P
**34**	PA121[PER]
**35**	PA13[PER]
**36**	NA26
**37**	NA232
**38**	NA399
**39**	SCA11
**40**	SCA9
**41**	U45[PER]
**42**	U70[PER]
**43**	IMC60
**44**	IMC103
**45**	COCA3370/5[CHA]
**46**	POUND12/A
**47**	LCTEEN163/A
**48**	LCTEEN401
**49**	LCTEEN261/S-4
**50**	NAPO25[CHA]
**51**	RB47[BRA]
**52**	LCTEEN412
**53**	EBC148
**54**	RB46[BRA]
**55**	ICS1
**56**	UF676
**57**	ICS95
**58**	UF613
**59**	MO20
**60**	U26[PER]
**61**	EET183[ECU]
**62**	EET19[ECU]
**63**	EET95[ECU]
**64**	CRIOLLO21[CRI]
**65**	CRIOLLO11[CRI]

Y –axis: ΔCq (Cq_{ref} –Cq_{alt})

Set Thresholds:

---------------- (black, striped line) = Manual upper and lower boundary

…………………. (blue, pointed line) = Calculated upper and lower boundary (µ ± 3xSD(H)[1])

⬄ Boundaries were set at 0 for certain SNP markers, as they could not be calculated.

[1] with µ and SD(H) representing the average and standard deviation for all ΔCq values from the validated heterozygous samples

Table of content

1. TcSNP139
2. TcSNP144
3. TcSNP151
4. TcSNP174
5. TcSNP193
6. TcSNP226
7. TcSNP230
8. TcSNP364
9. TcSNP372
10. TcSNP413
11. TcSNP448
12. TcSNP577
13. TcSNP591
14. TcSNP602
15. TcSNP606
16. TcSNP751
17. TcSNP786
18. TcSNP852
19. TcSNP860
20. TcSNP872
21. TcSNP878
22. TcSNP891
23. TcSNP994
24. TcSNP1041
25. TcSNP1060
26. TcSNP1111
27. TcSNP1126
28. TcSNP1280
29. TcSNP1331
30. TcSNP1439
31. TcSNP1458
32. Tcm002s00644224
33. Tcm002s29938429
34. Tcm002s34015437
35. Tcm003s05554949
36. Tcm003s12502217
37. Tcm003s20315420
38. Tcm003s27807955
39. Tcm006s26507164
40. Tcm008s17168944
41. Tcm009s28255143

42. Tcm009s41415628

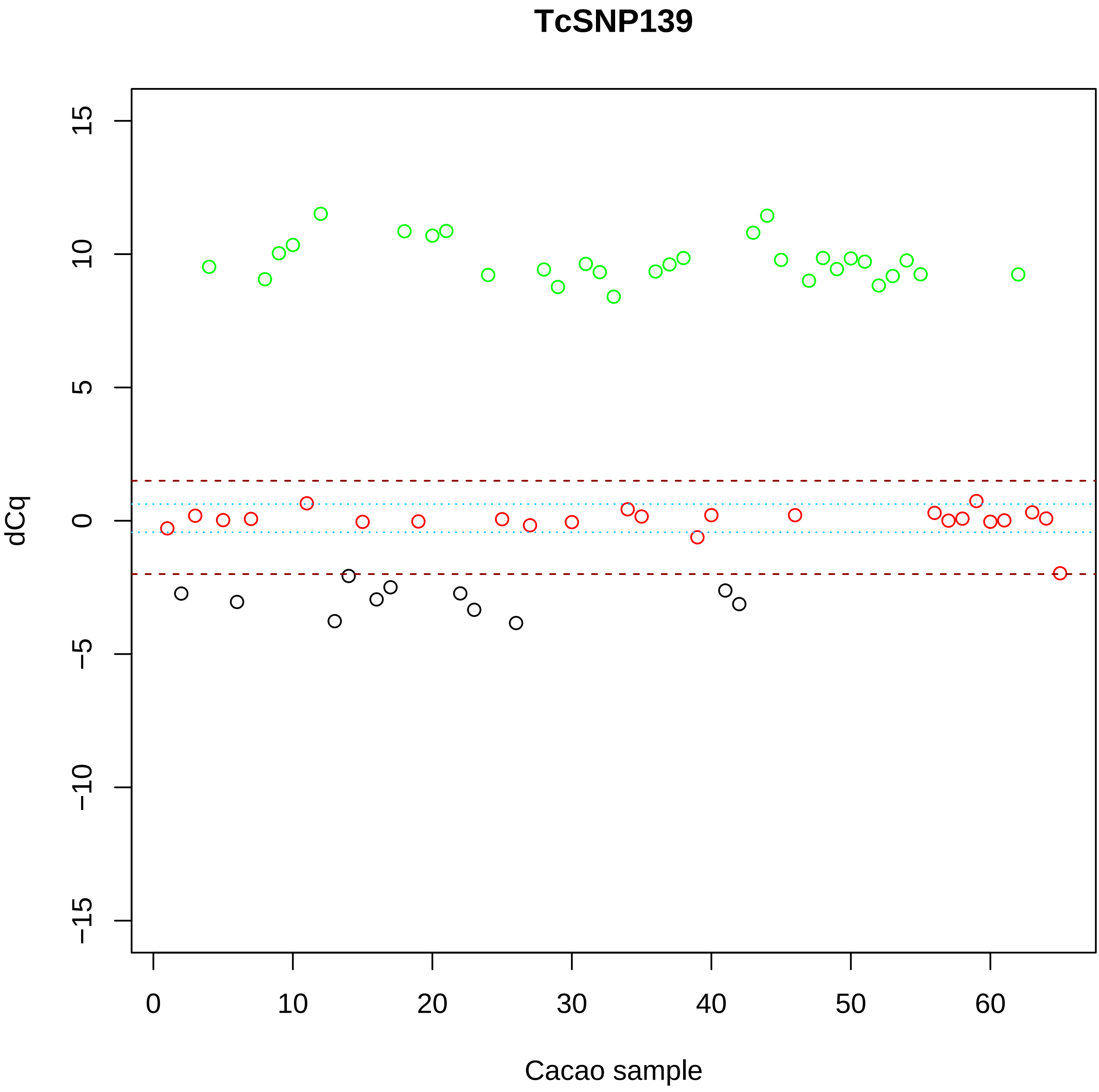

TcSNP139
dCq
Cacao sample
15
10
5
0
−5
−10
−15
0
10
20
30
40
50
60

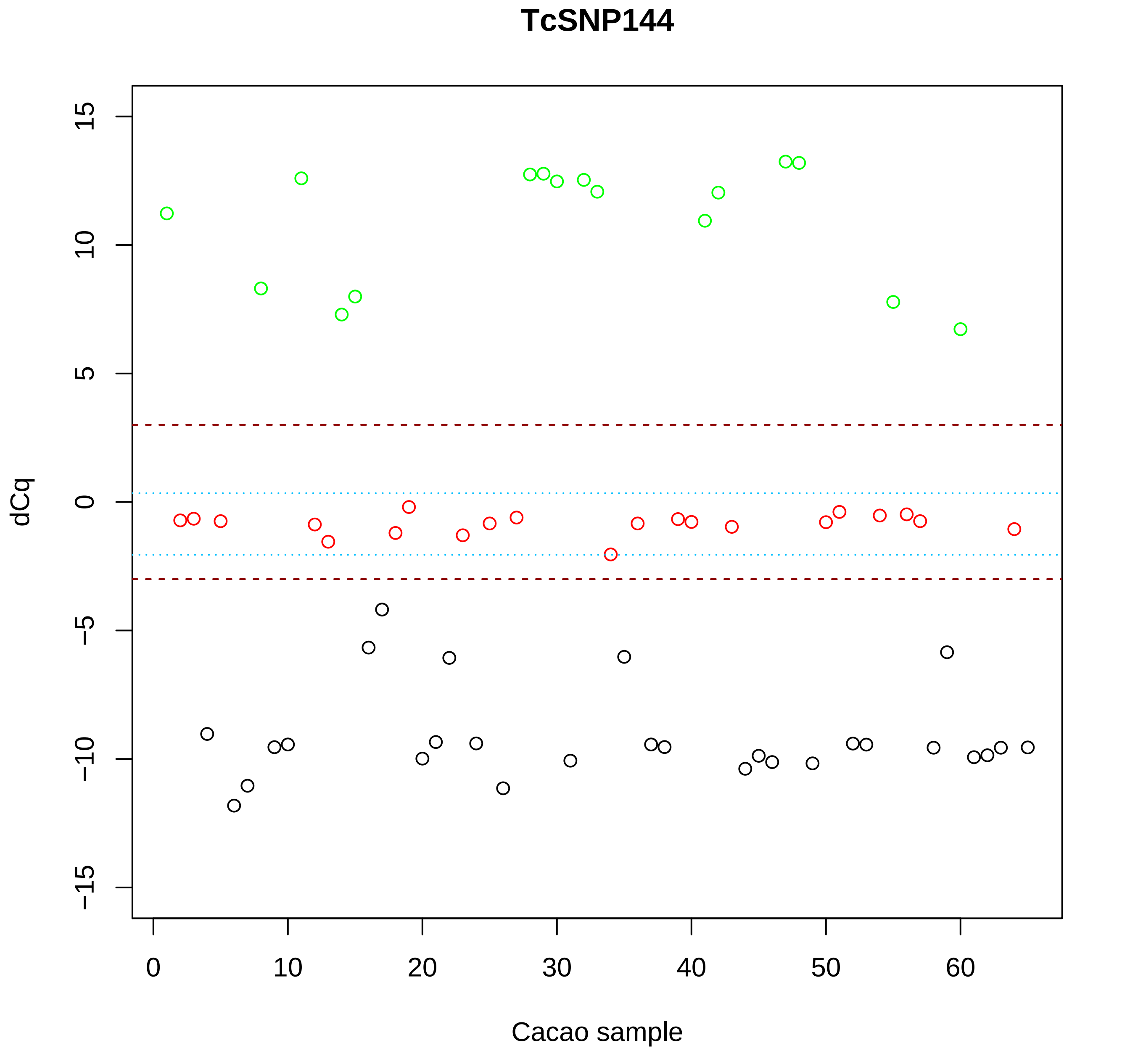
TcSNP144
dCq
Cacao sample
15
10
5
0
−5
−10
−15
0
10
20
30
40
50
60

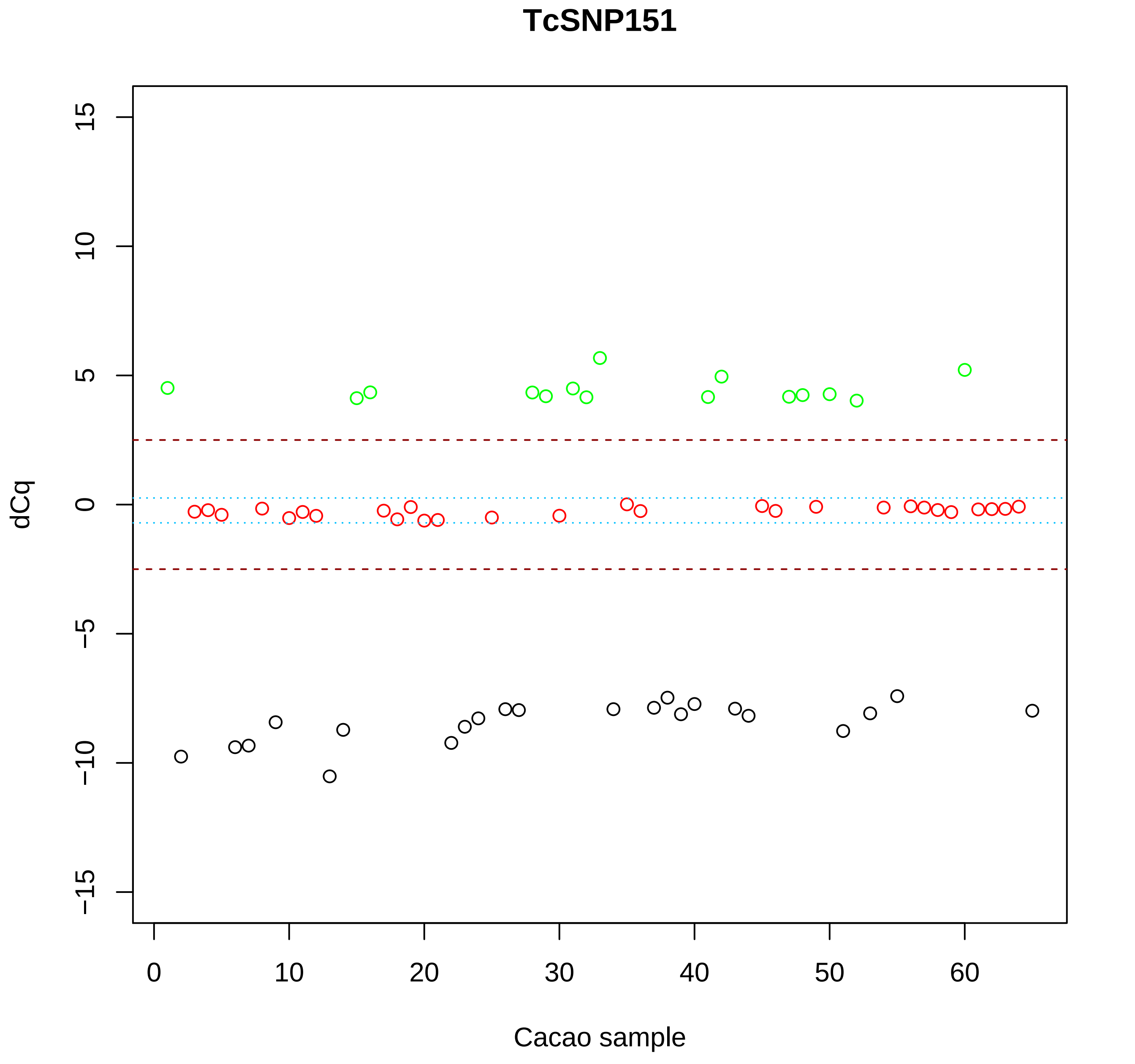

TcSNP151
dCq
Cacao sample
15
10
5
0
−5
−10
−15
0
10
20
30
40
50
60

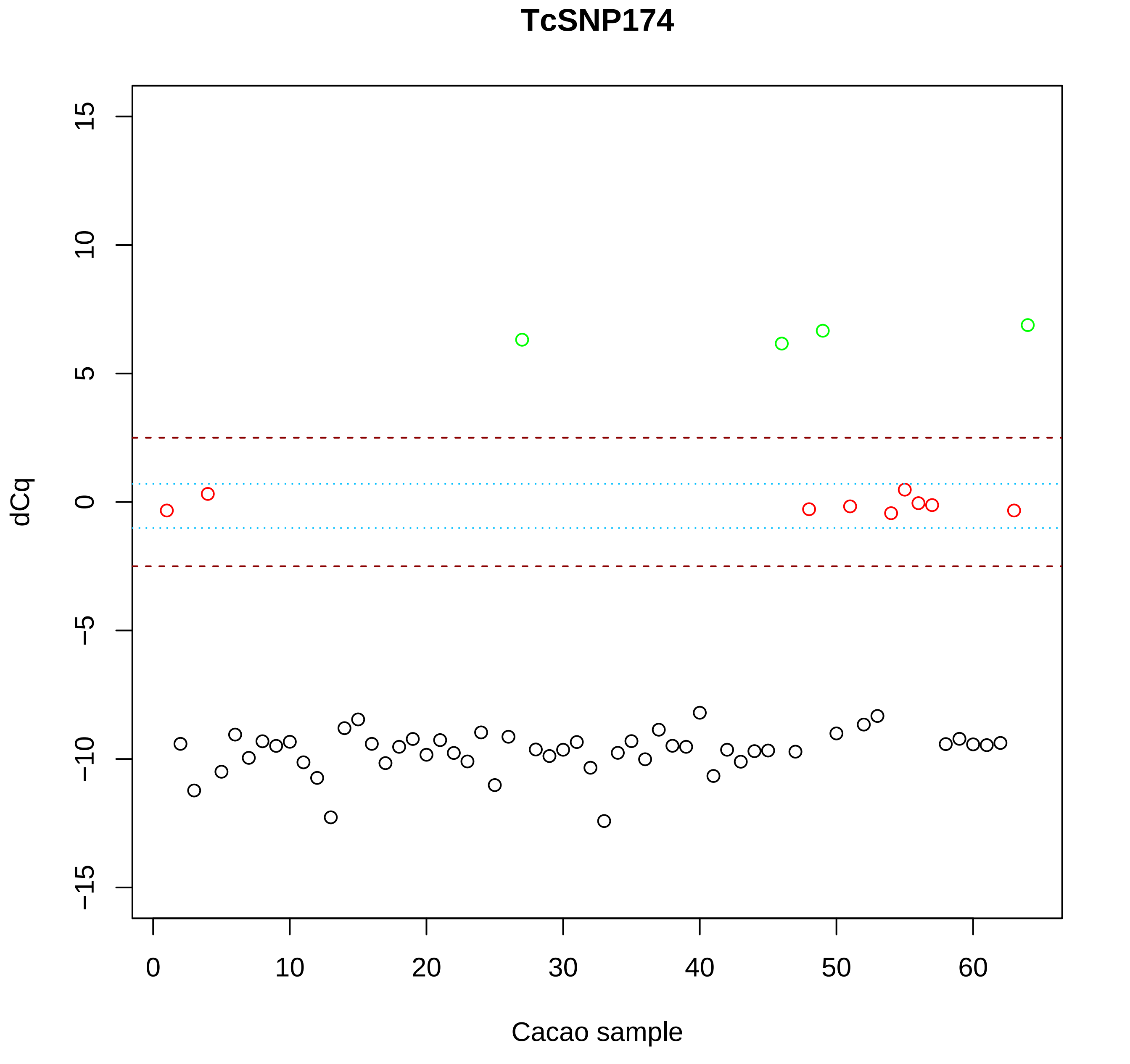
TcSNP174
dCq
15
10
5
0
−5
−10
−15
0
10
20
30
40
50
60
Cacao sample

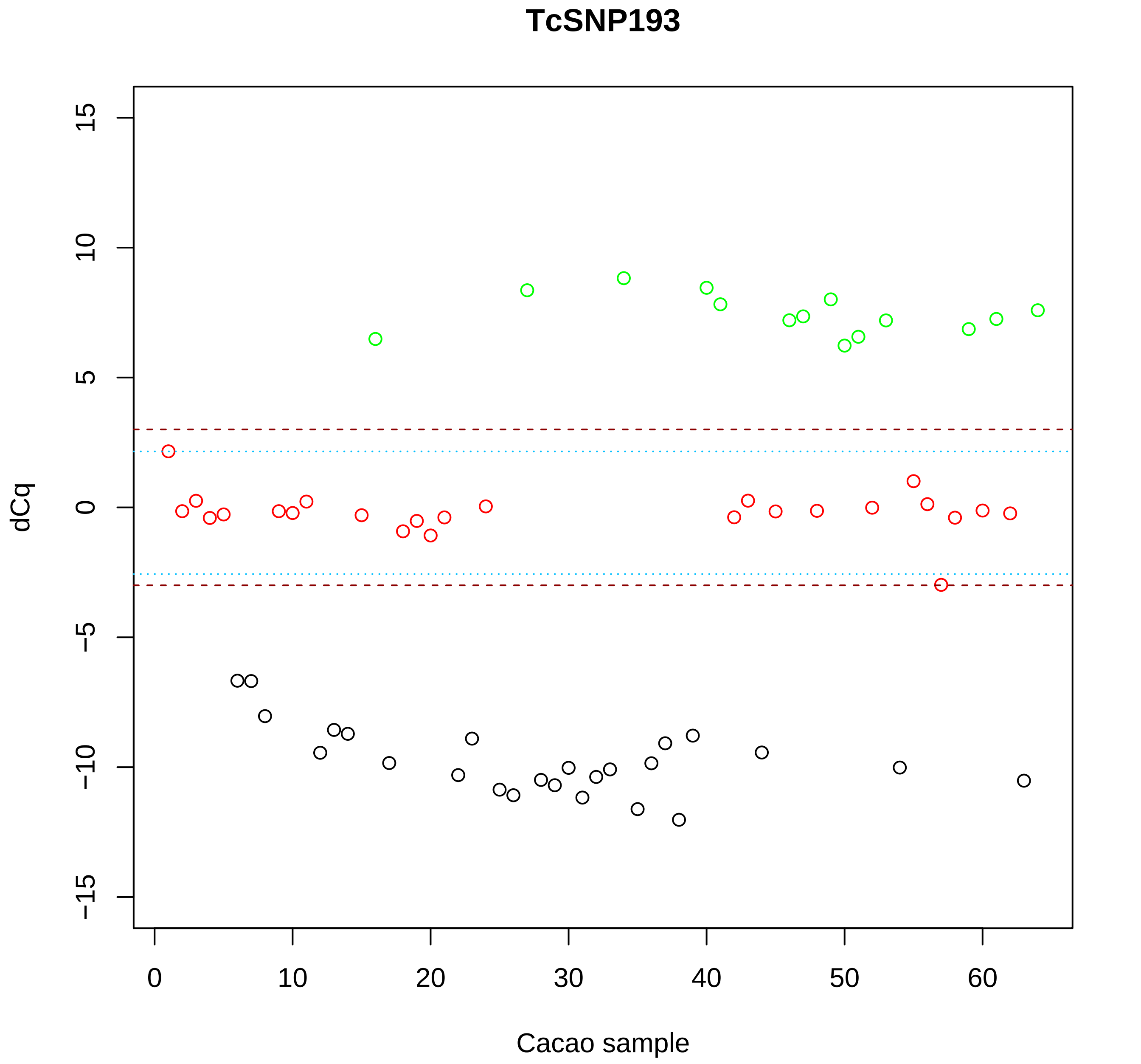
TcSNP193
dCq
15
10
5
0
−5
−10
−15
0
10
20
30
40
50
60
Cacao sample

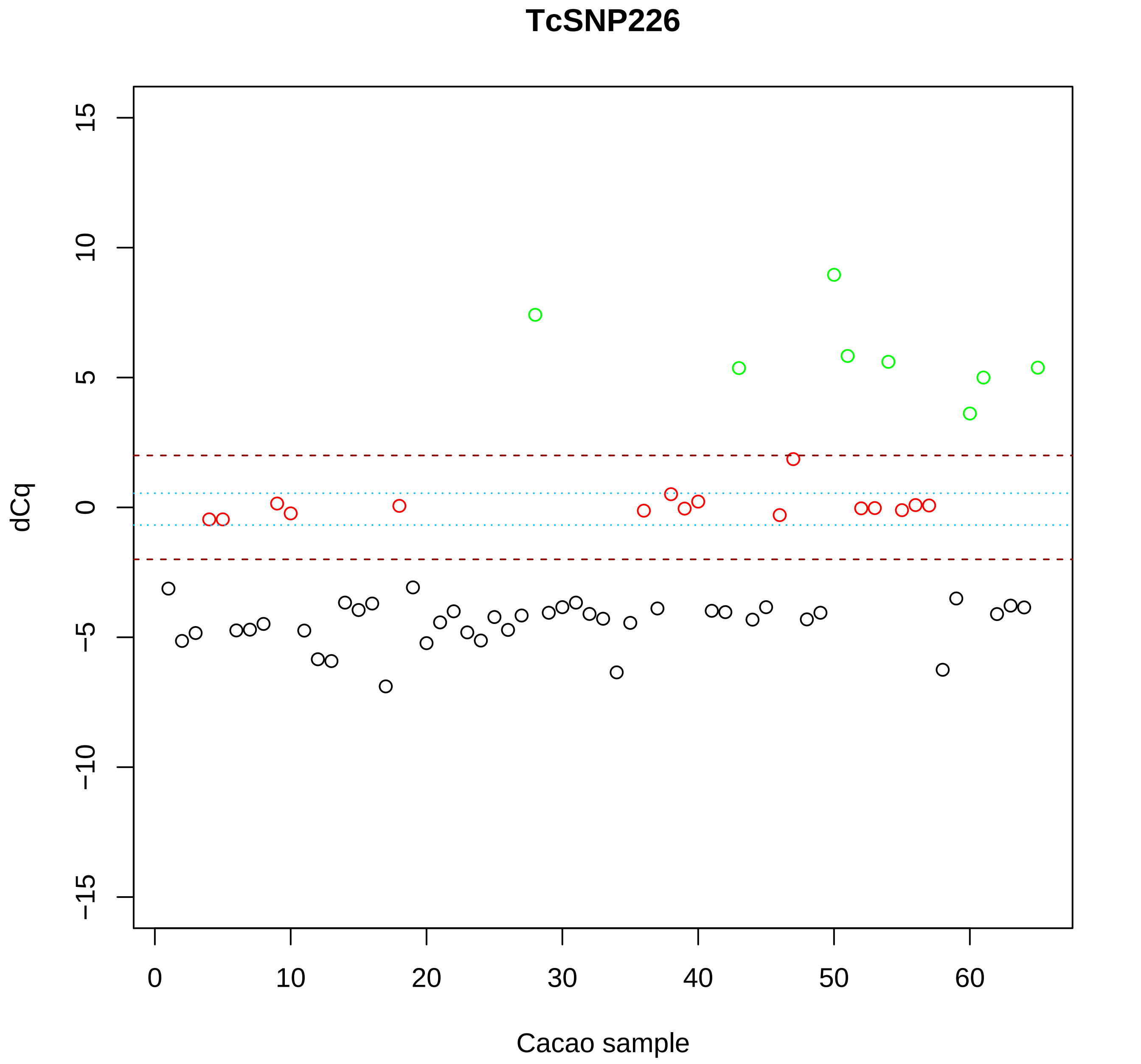
TcSNP226
dCq
Cacao sample
15
10
5
0
−5
−10
−15
0
10
20
30
40
50
60

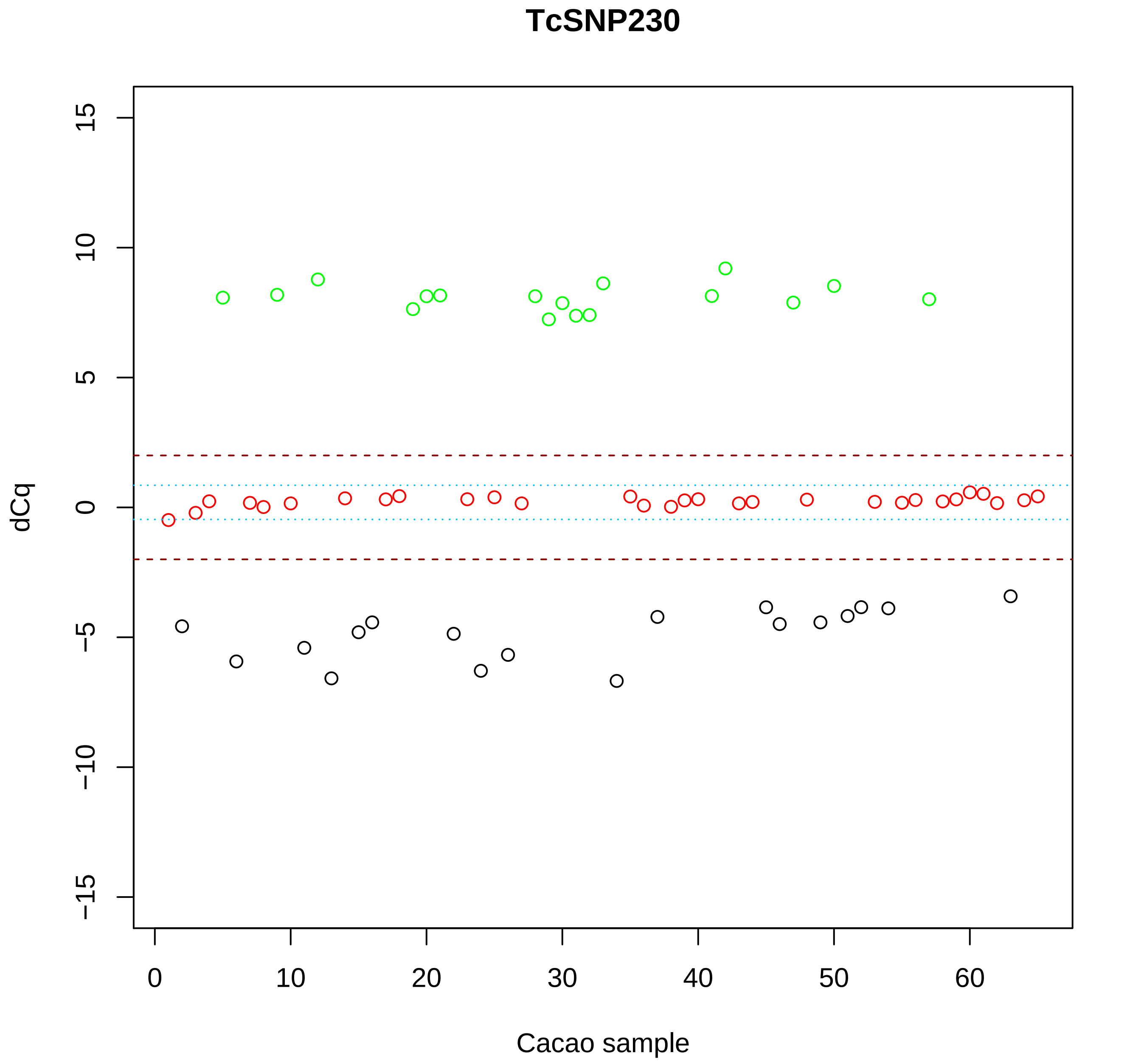
TcSNP230
dCq
15
10
5
0
−5
−10
−15
0
10
20
30
40
50
60
Cacao sample

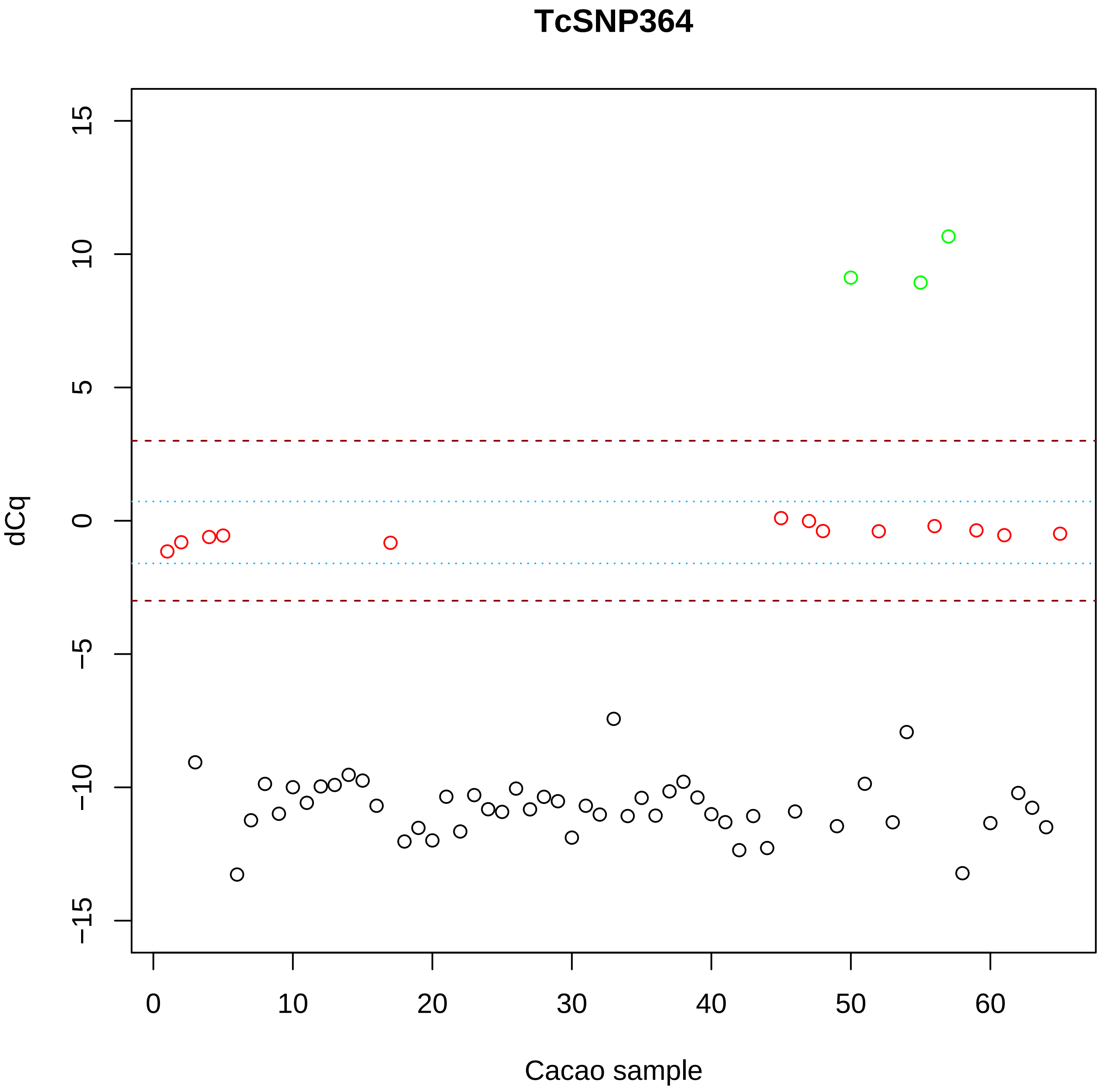
TcSNP364
dCq
Cacao sample
15
10
5
0
−5
−10
−15
0
10
20
30
40
50
60

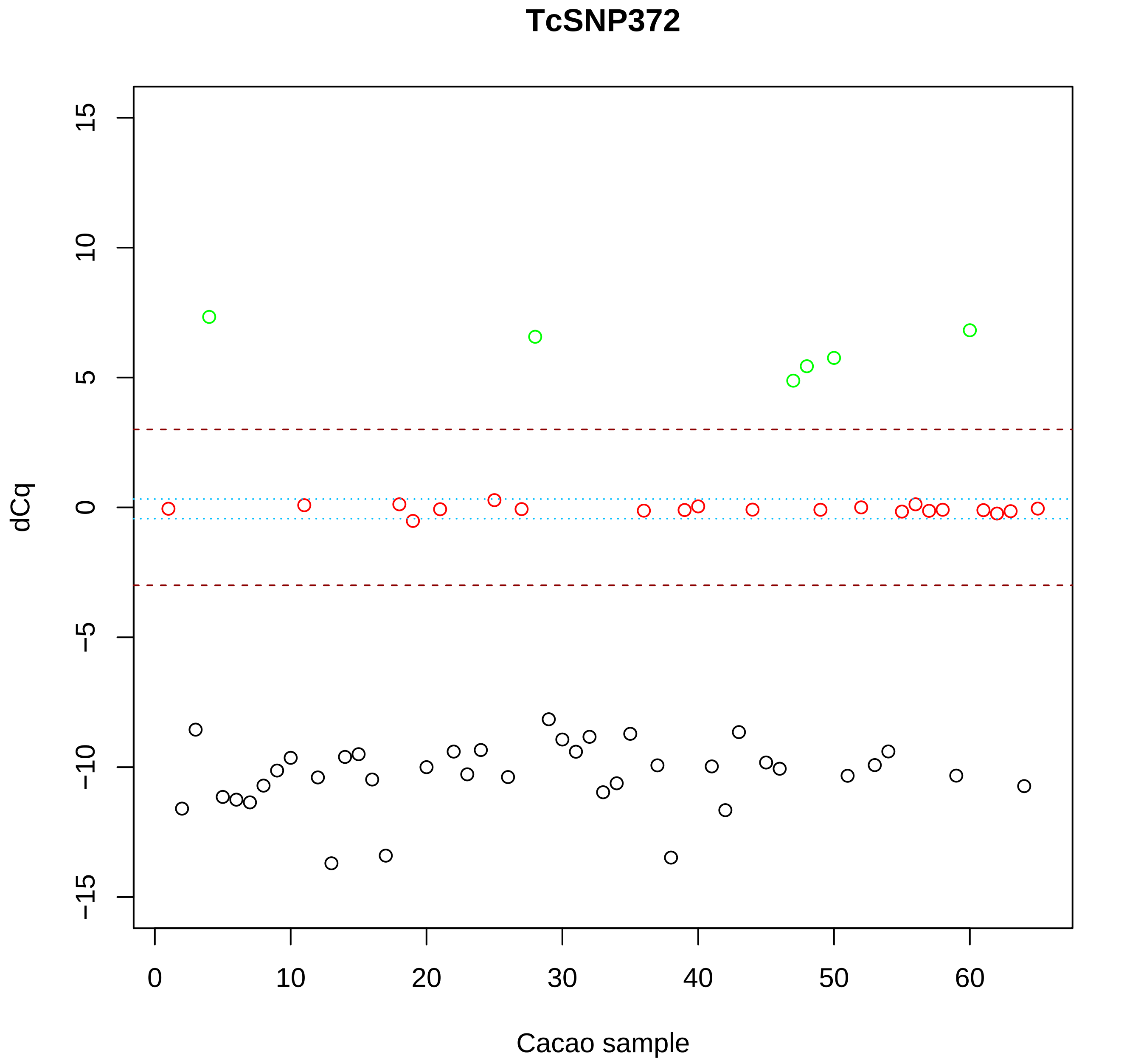
TcSNP372
dCq
Cacao sample
15
10
5
0
−5
−10
−15
0
10
20
30
40
50
60

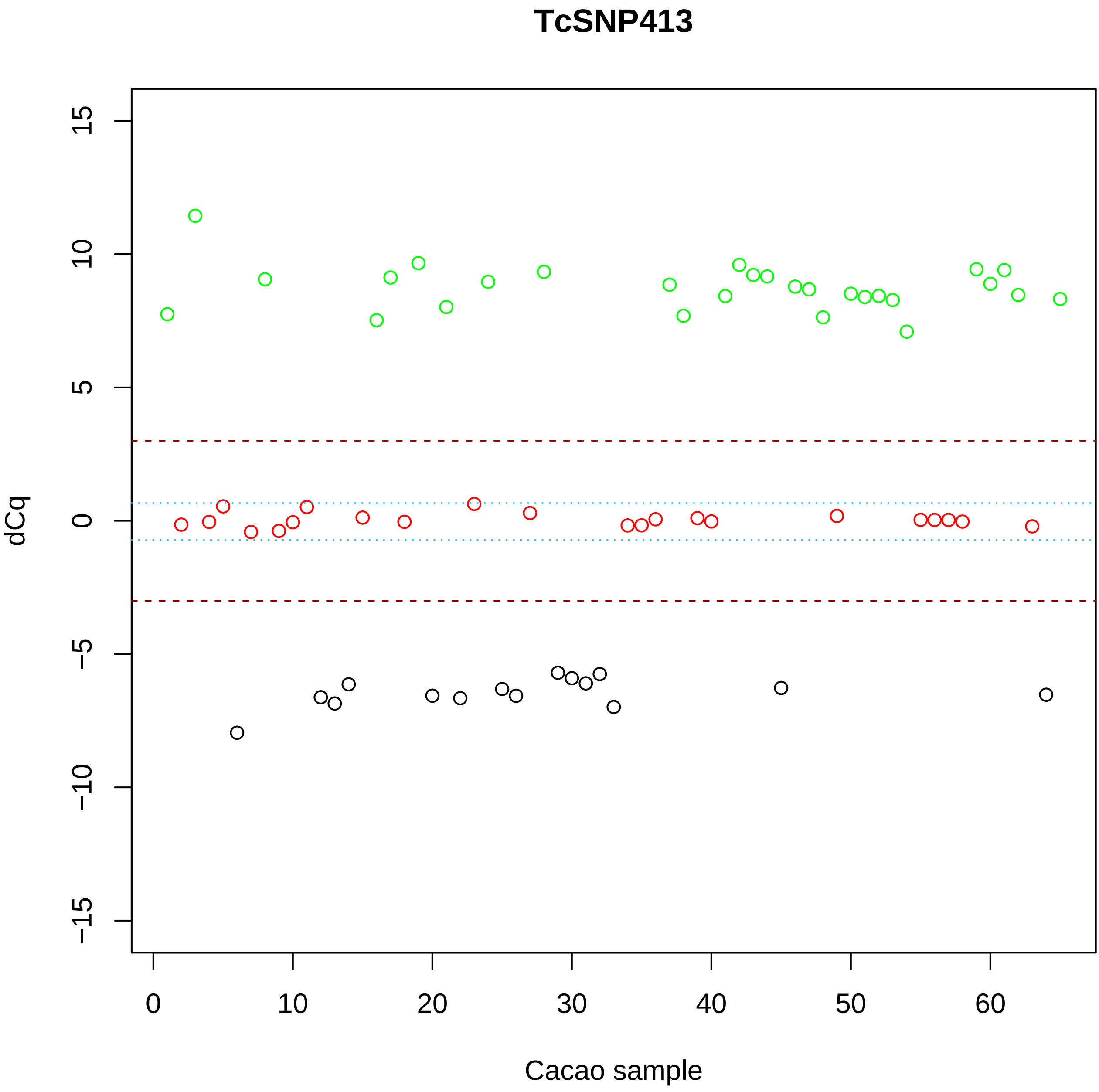
TcSNP413
dCq
Cacao sample
15
10
5
0
−5
−10
−15
0
10
20
30
40
50
60

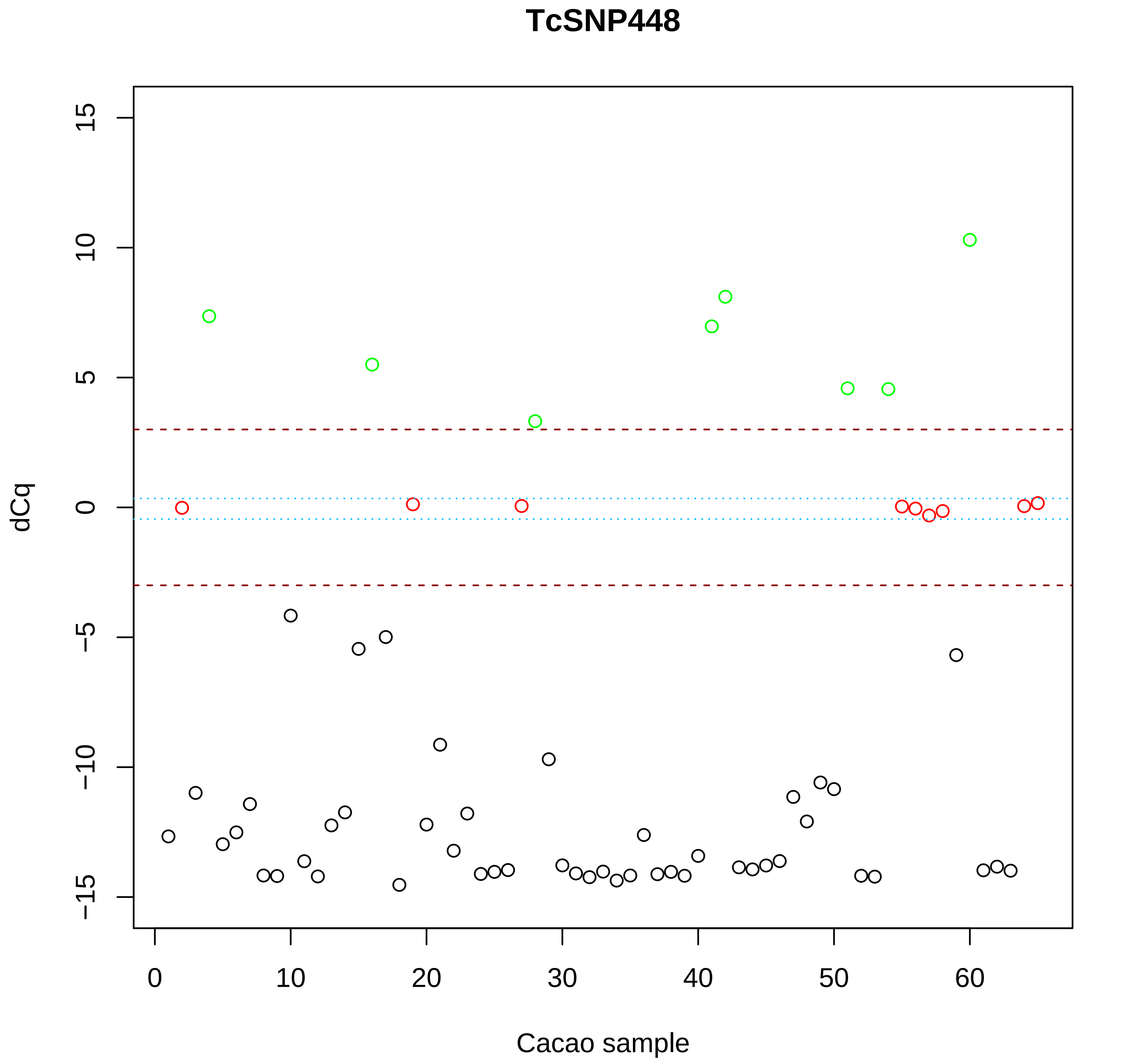

TcSNP448
dCq
Cacao sample
15
10
5
0
−5
−10
−15
0
10
20
30
40
50
60

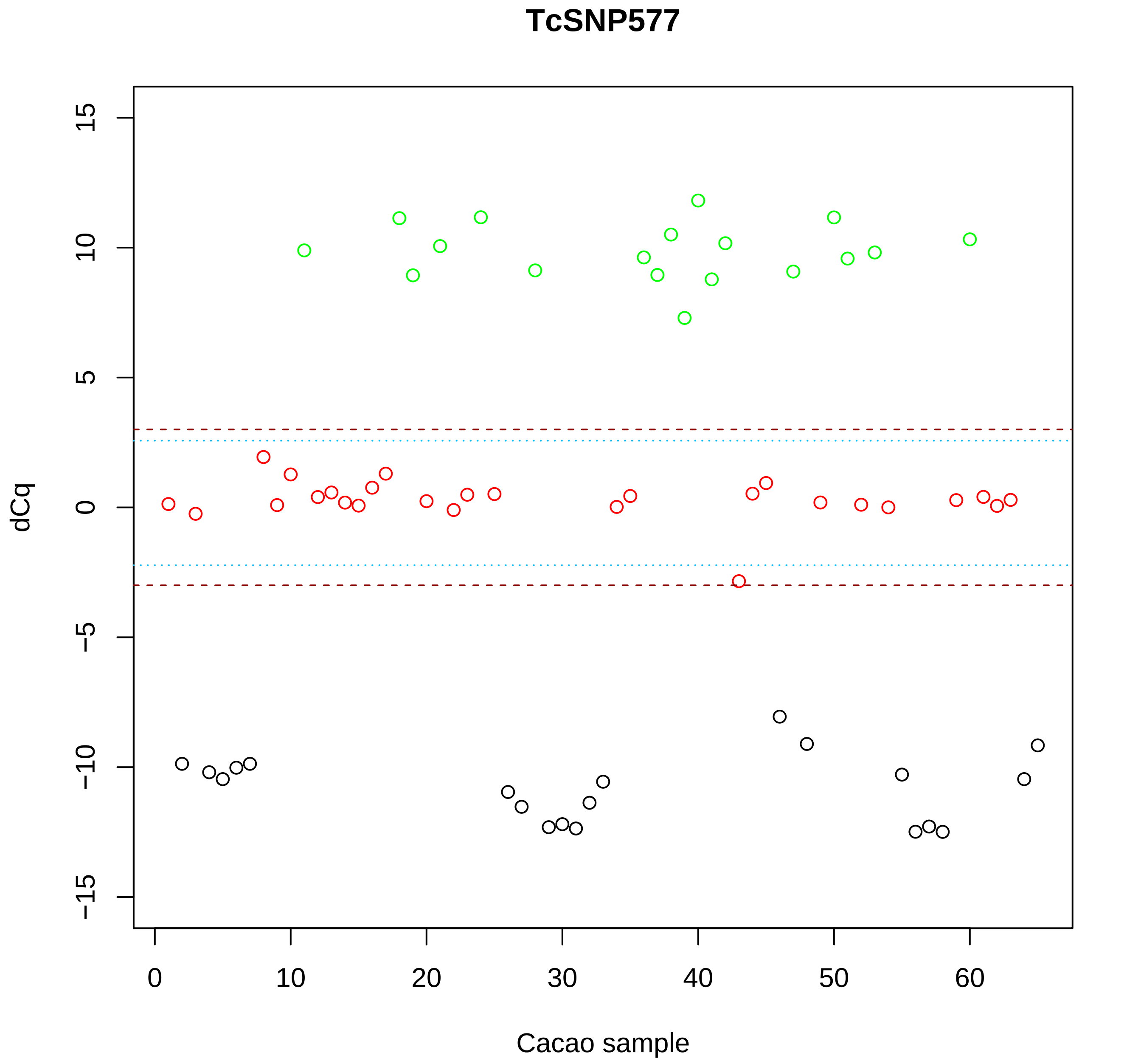
TcSNP577
dCq
Cacao sample
15
10
5
0
−5
−10
−15
0
10
20
30
40
50
60

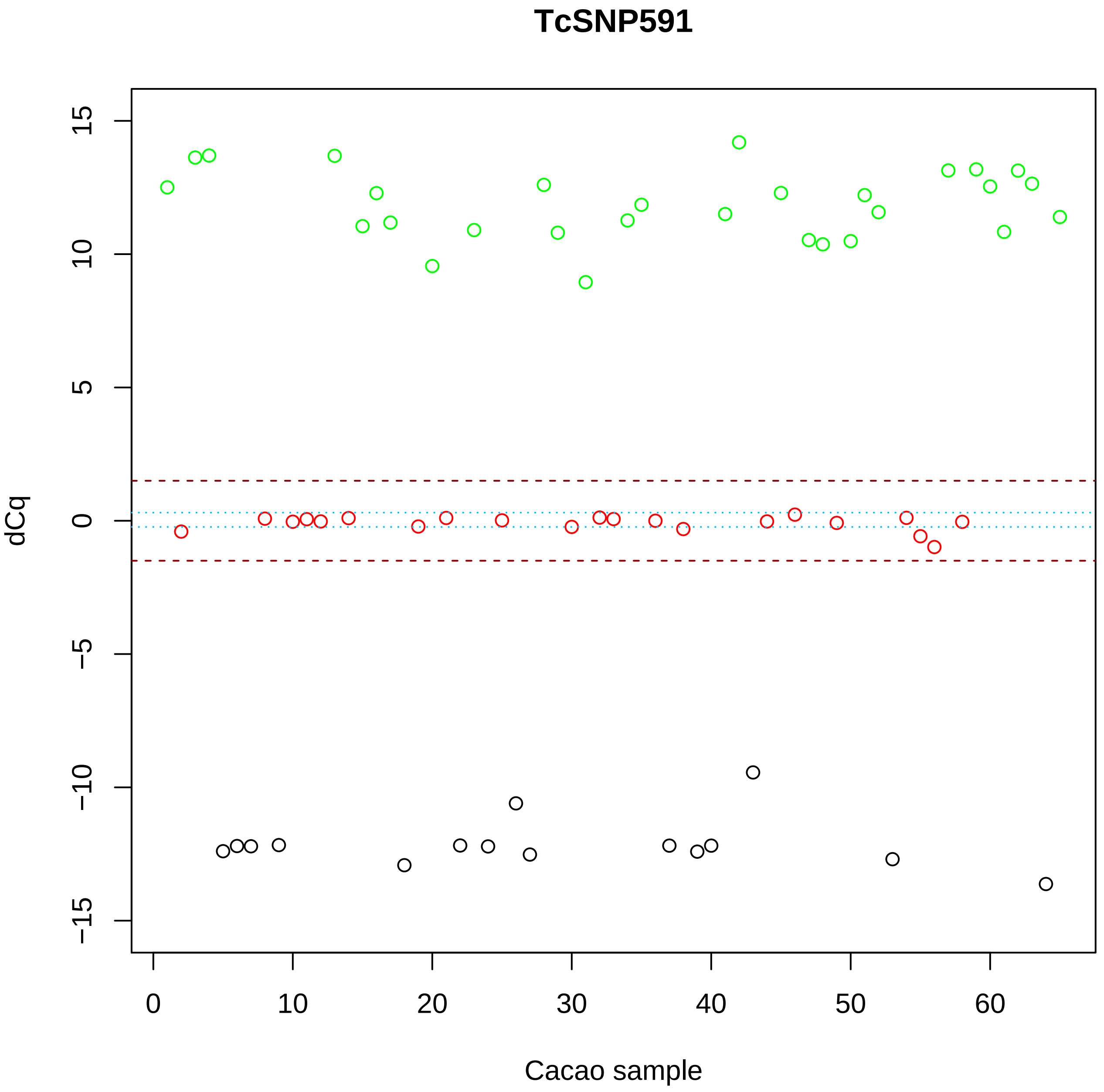

TcSNP591
dCq
Cacao sample
15
10
5
0
−5
−10
−15
0
10
20
30
40
50
60

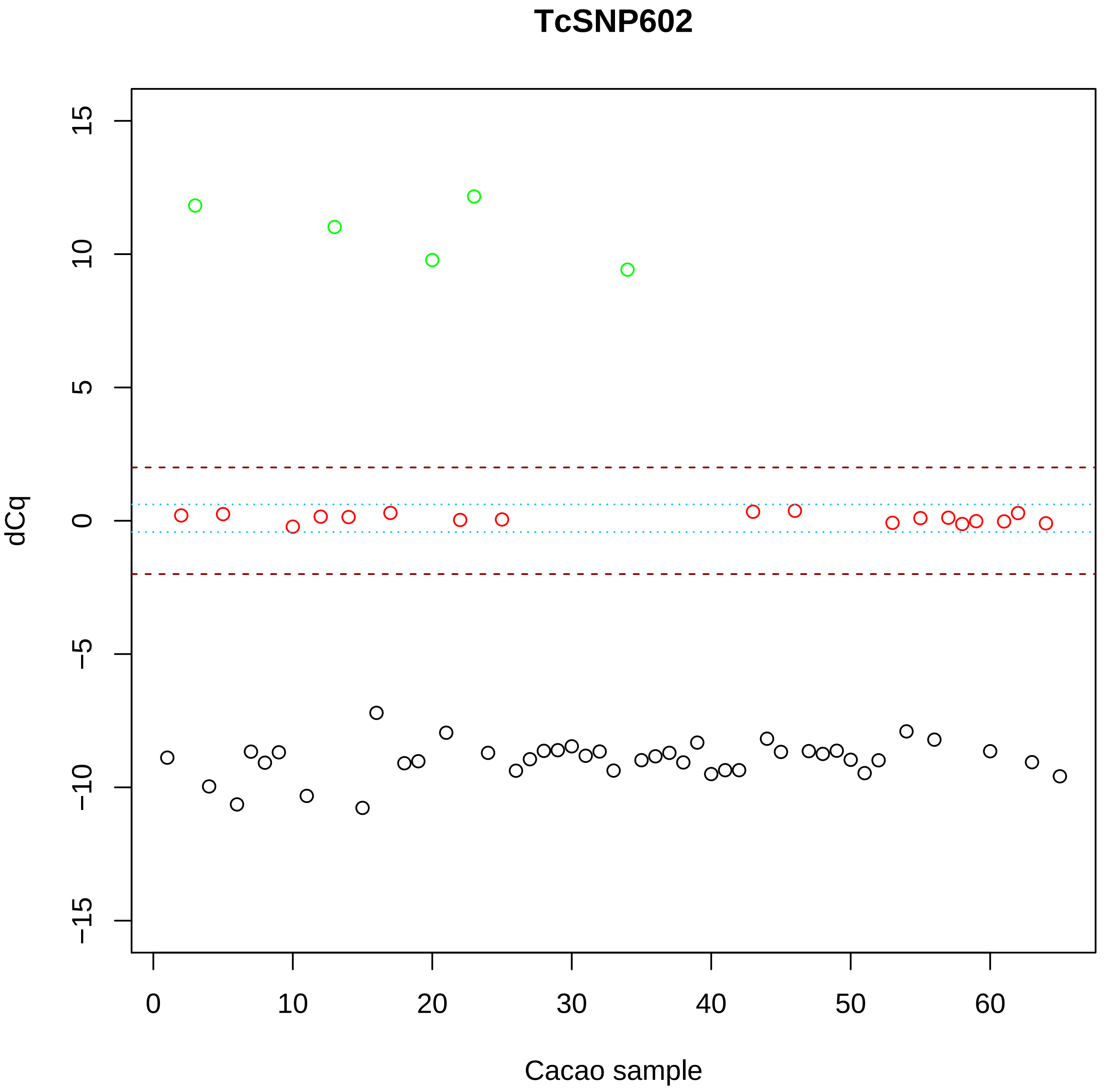
TcSNP602
dCq
Cacao sample
15
10
5
0
−5
−10
−15
0
10
20
30
40
50
60

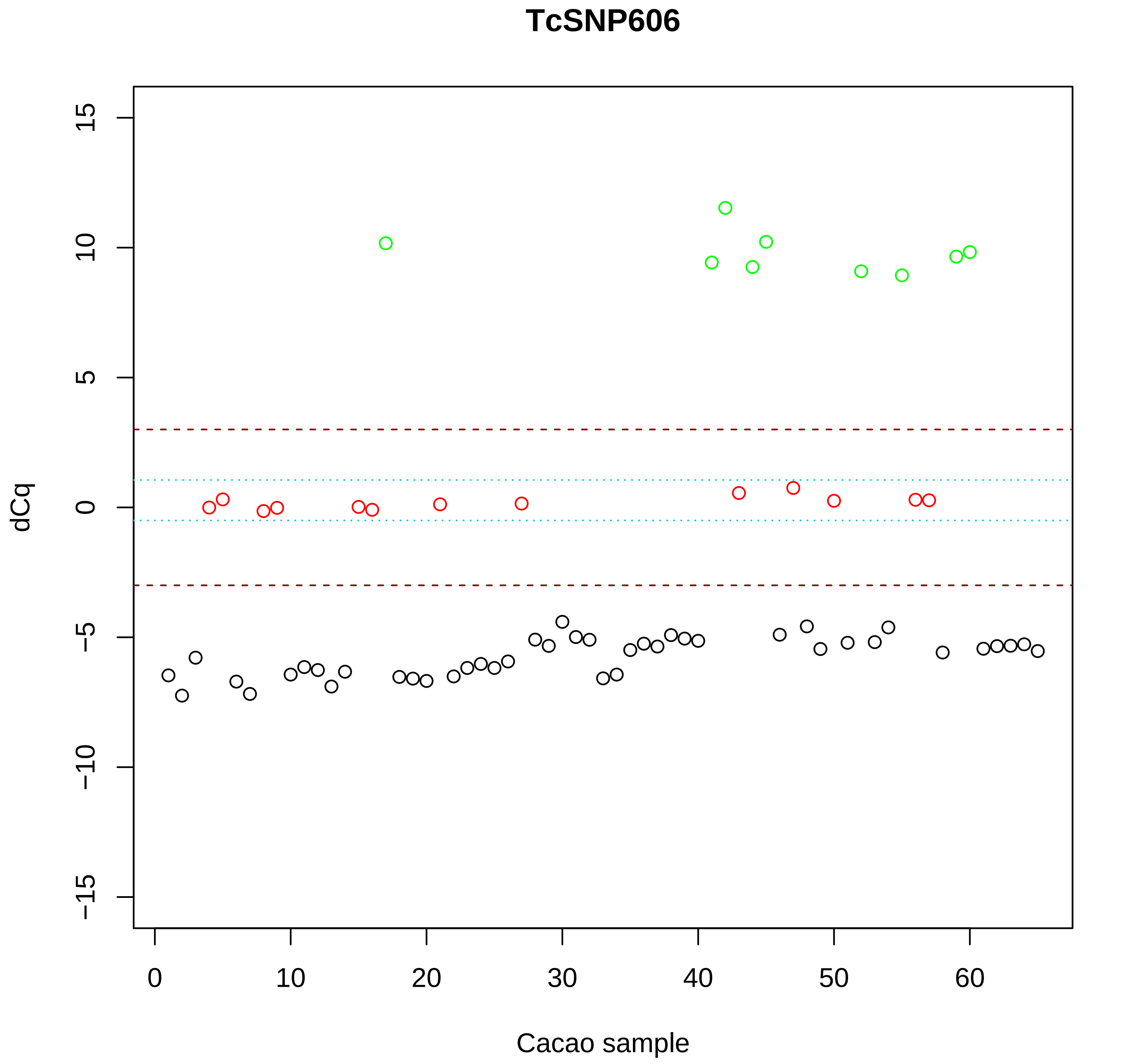
TcSNP606
dCq
Cacao sample
15
10
5
0
−5
−10
−15
0
10
20
30
40
50
60

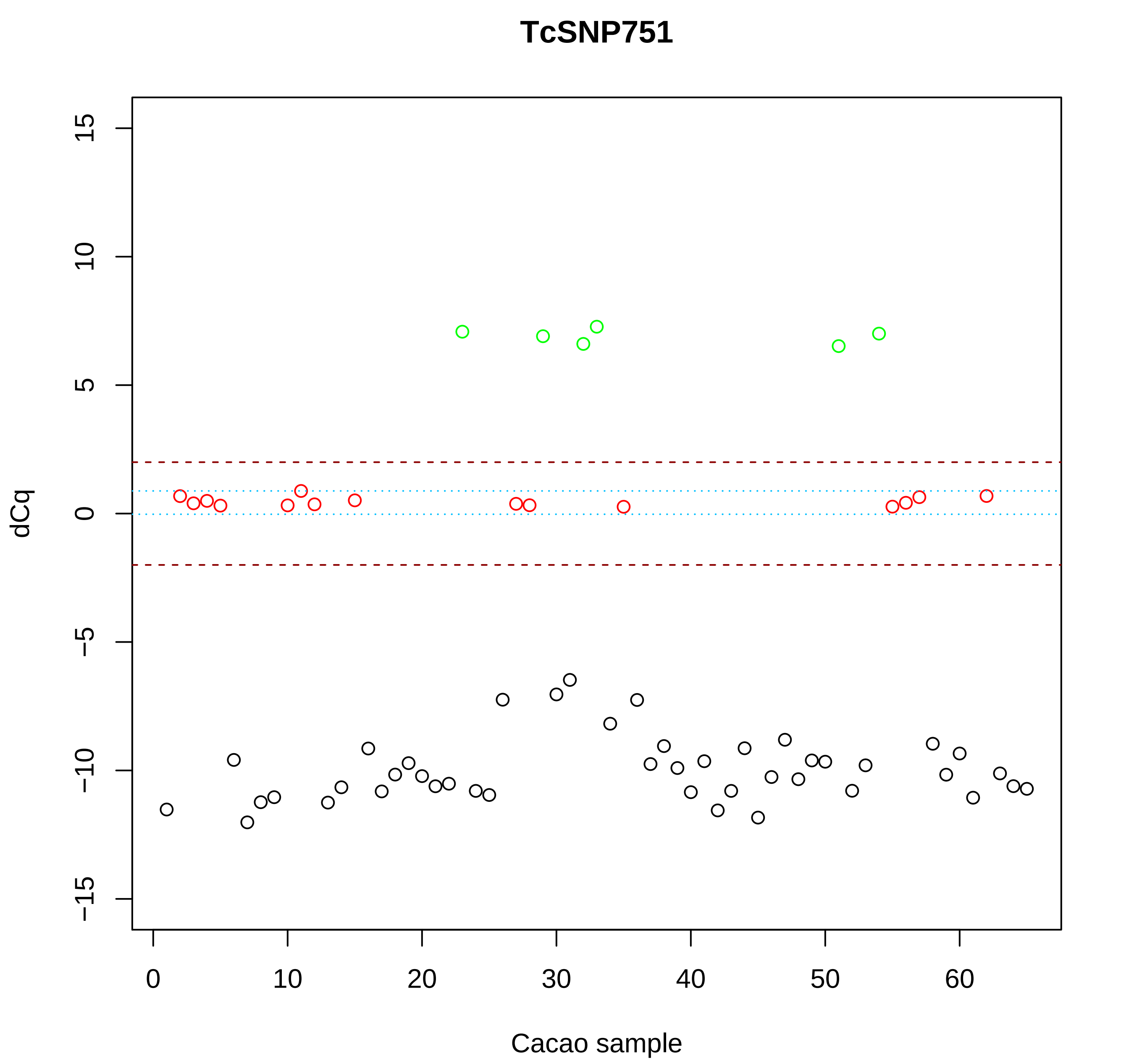

TcSNP751
dCq
15
10
5
0
−5
−10
−15
0
10
20
30
40
50
60
Cacao sample

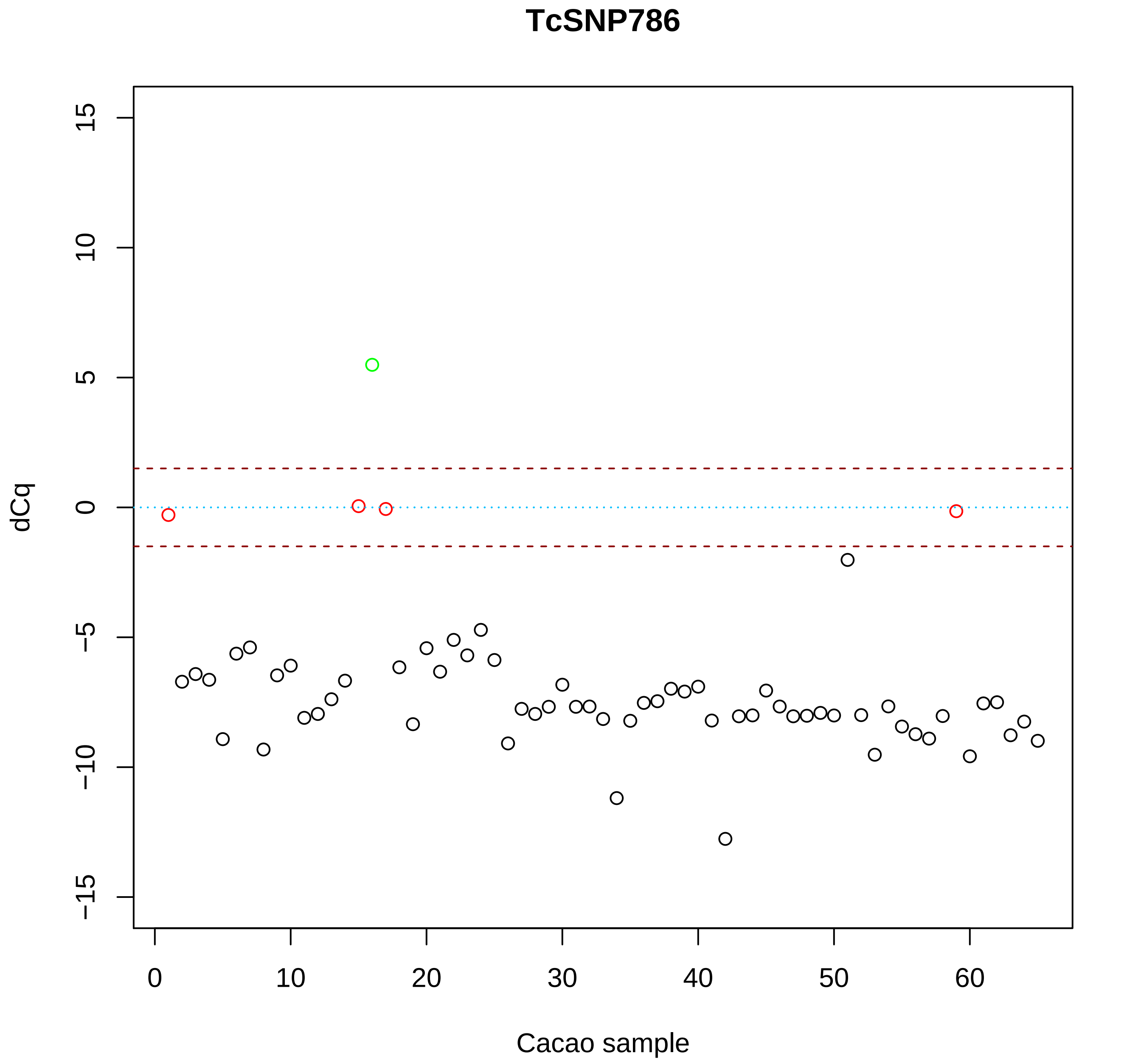
TcSNP786
dCq
15
10
5
0
−5
−10
−15
0
10
20
30
40
50
60
Cacao sample

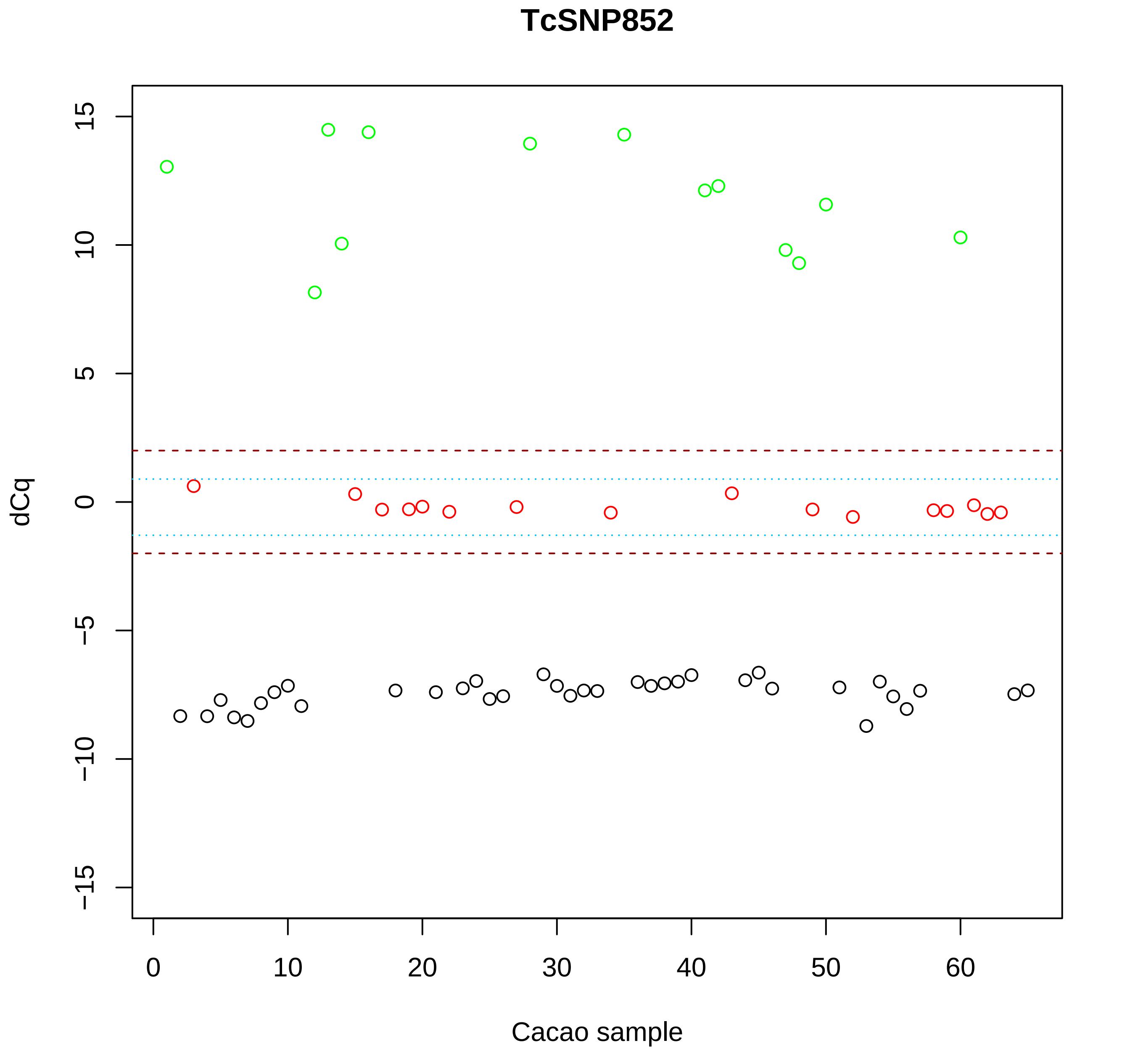
TcSNP852
dCq
Cacao sample
15
10
5
0
−5
−10
−15
0
10
20
30
40
50
60

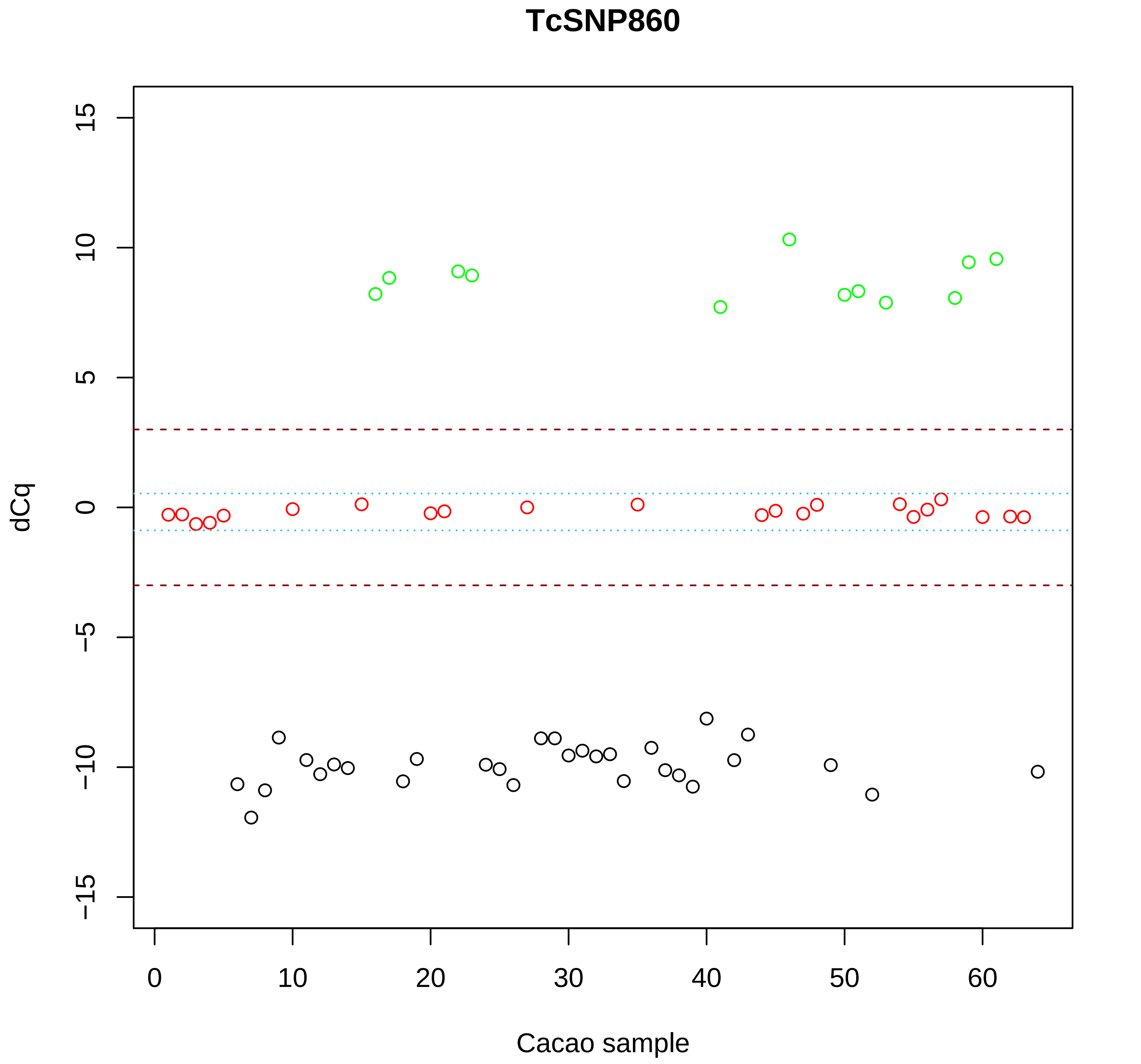
TcSNP860
dCq
Cacao sample
15
10
5
0
−5
−10
−15
0
10
20
30
40
50
60

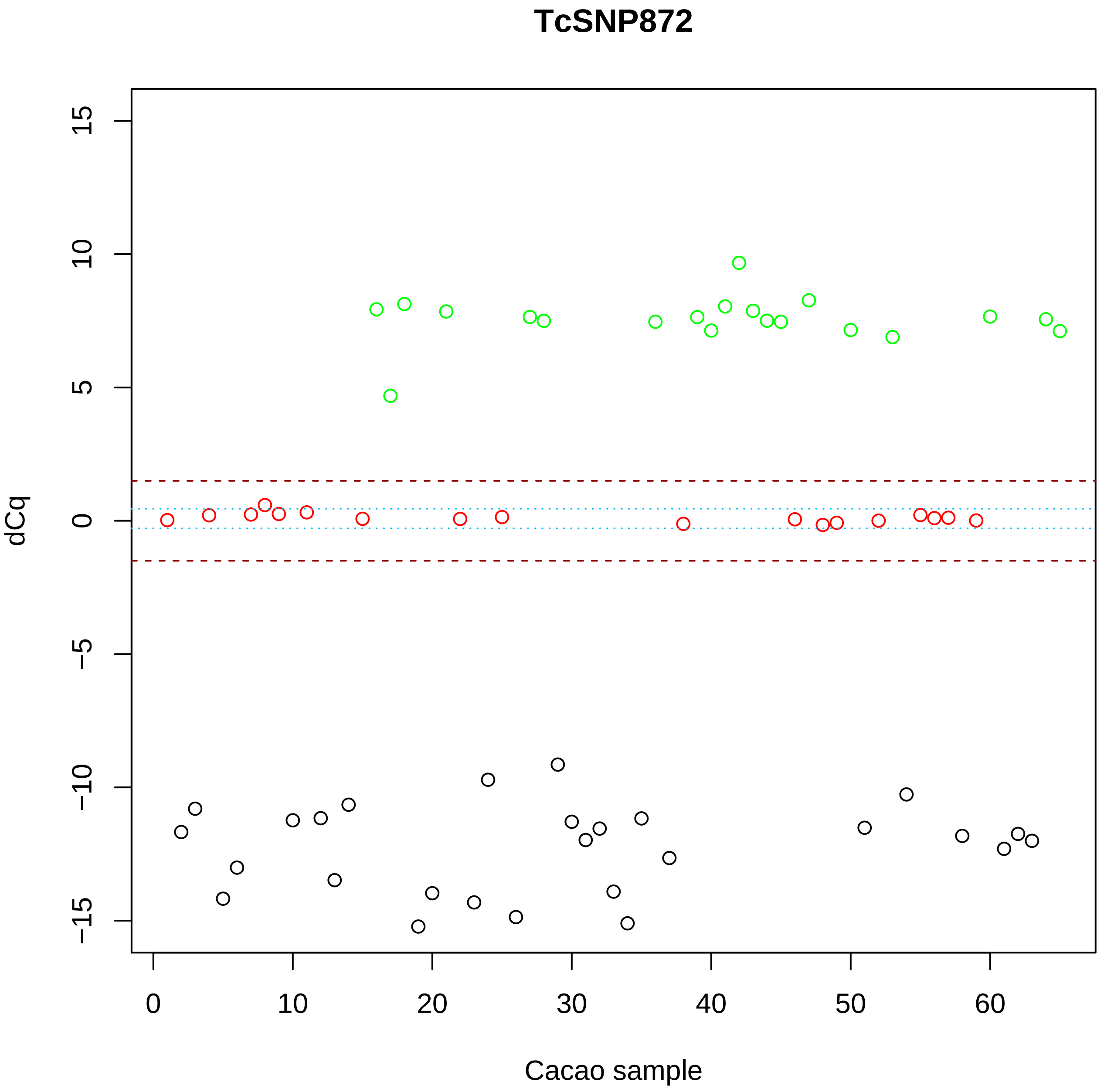

TcSNP872
dCq
Cacao sample
15
10
5
0
−5
−10
−15
0
10
20
30
40
50
60

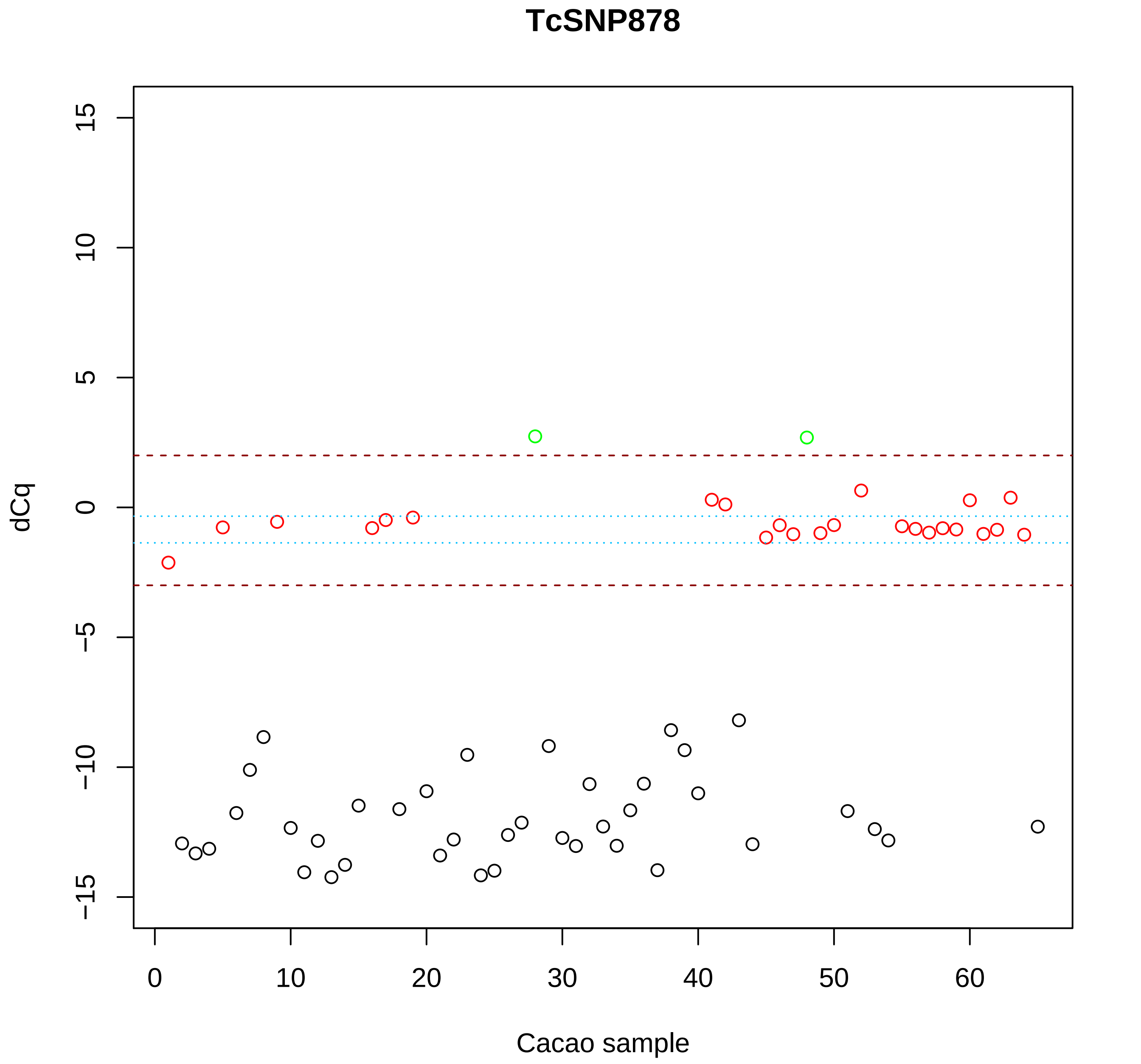

TcSNP878
dCq
Cacao sample
15
10
5
0
−5
−10
−15
0
10
20
30
40
50
60

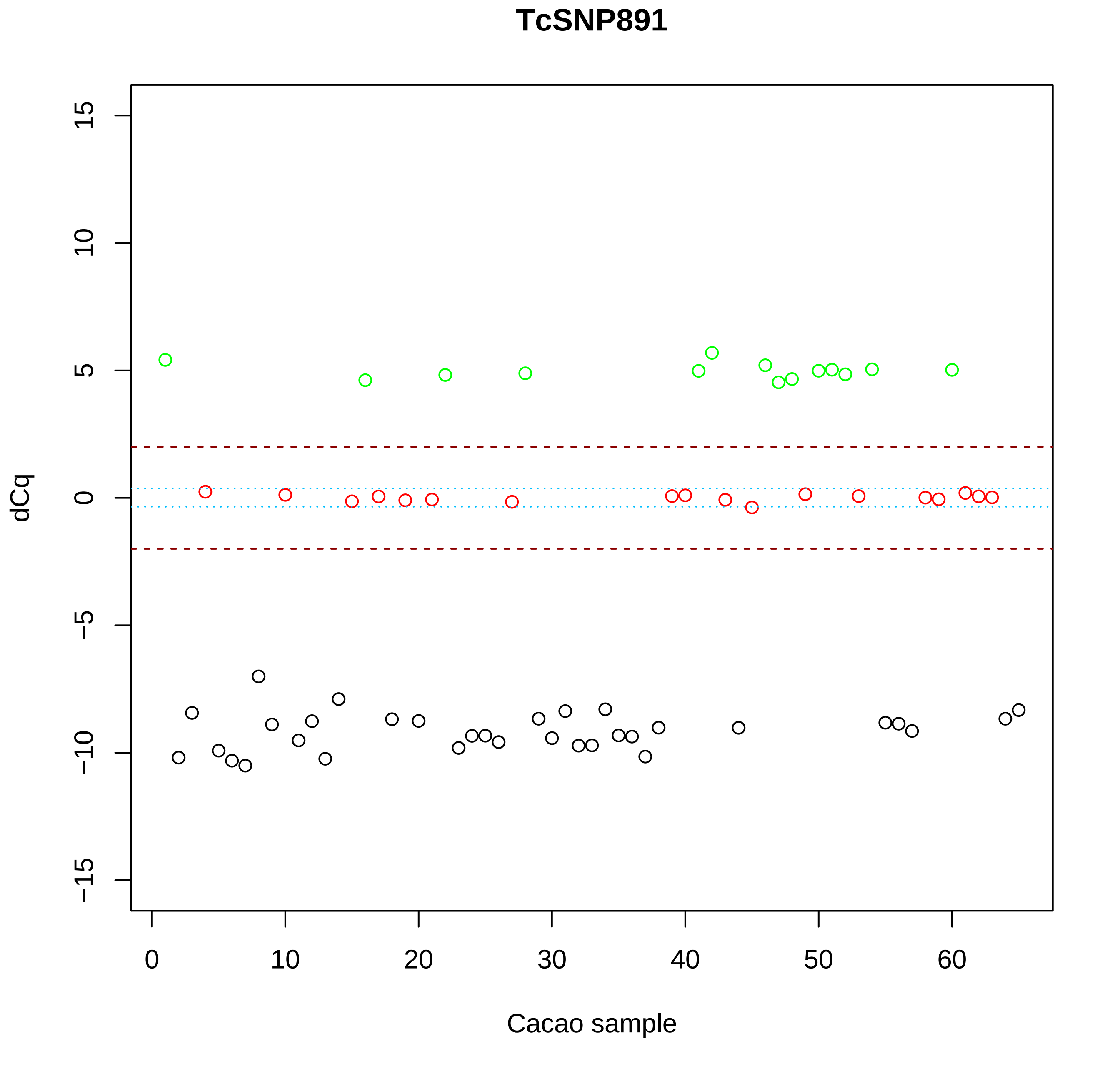

TcSNP891
dCq
Cacao sample
15
10
5
0
−5
−10
−15
0
10
20
30
40
50
60

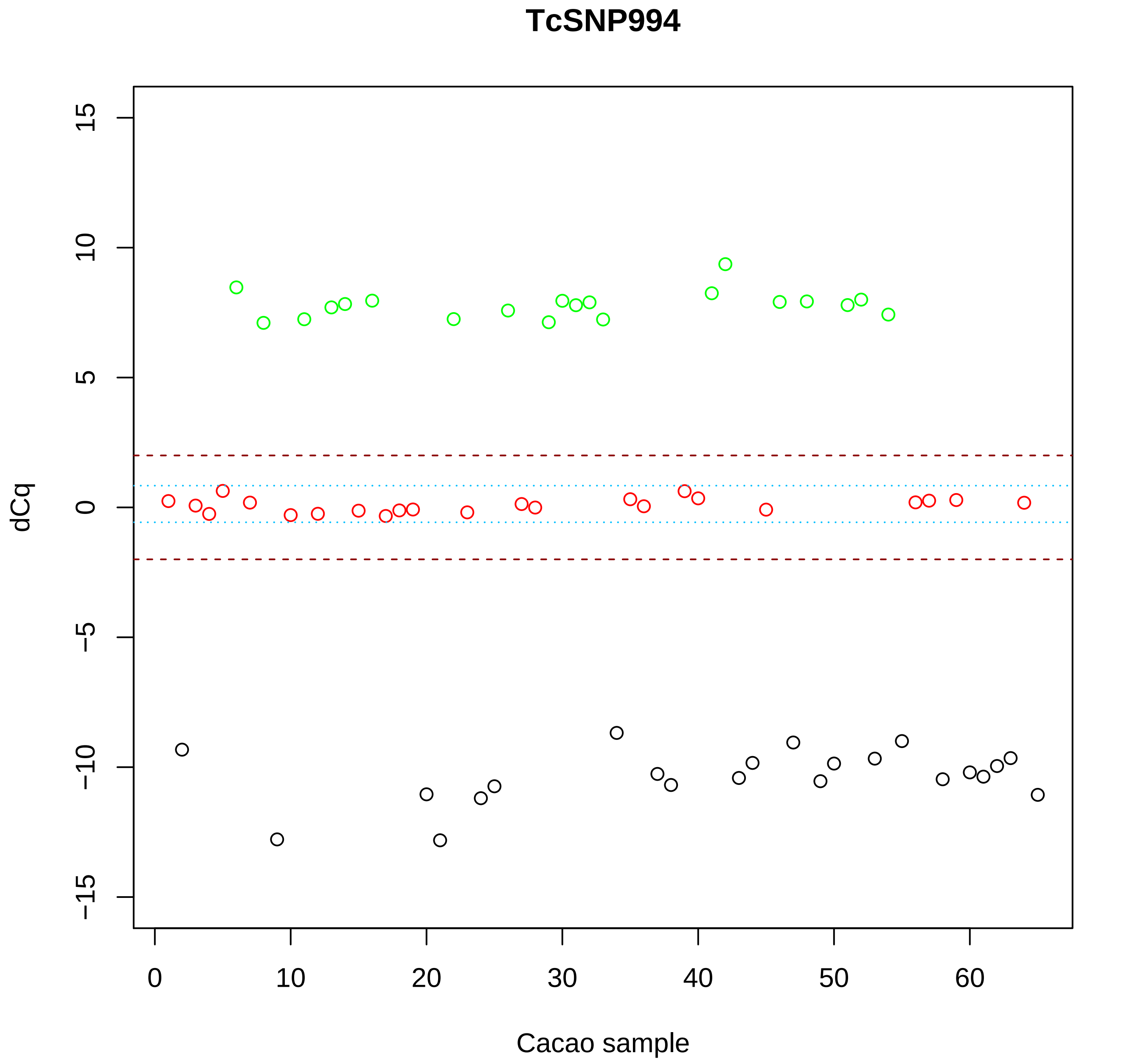
TcSNP994
dCq
15
10
5
0
−5
−10
−15
0
10
20
30
40
50
60
Cacao sample

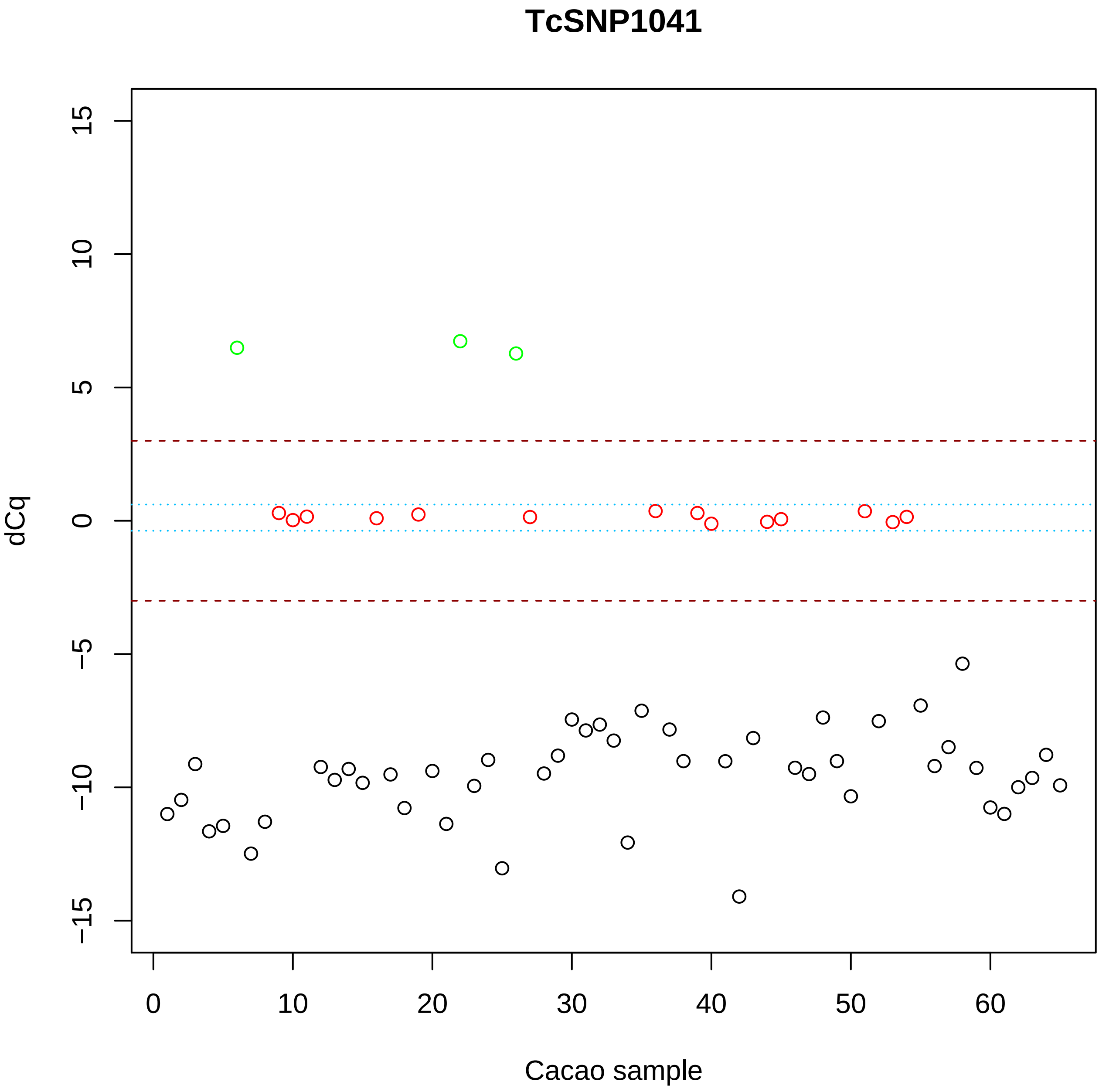

TcSNP1041
dCq
Cacao sample
15
10
5
0
−5
−10
−15
0
10
20
30
40
50
60

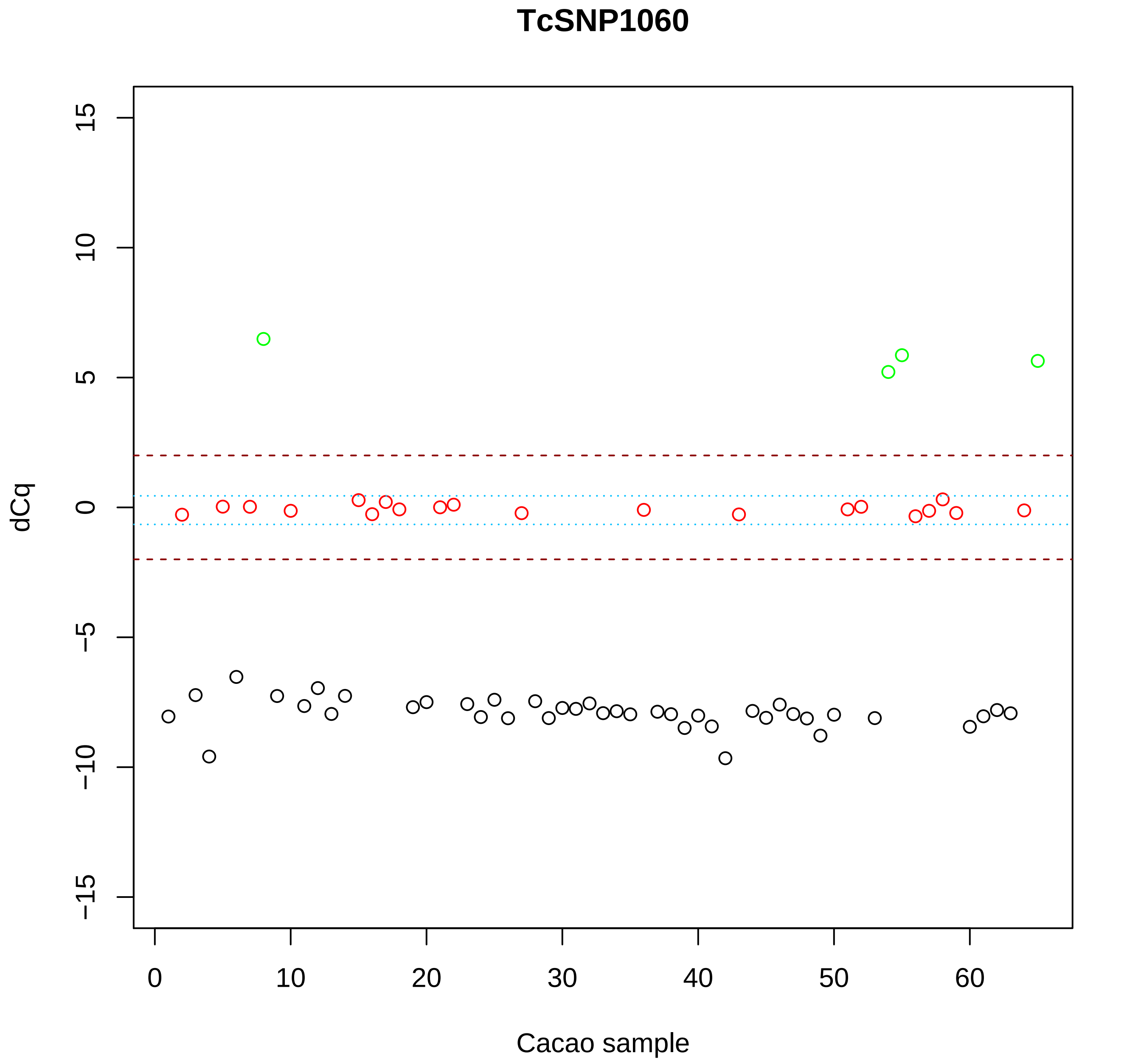

TcSNP1060
dCq
Cacao sample
15
10
5
0
−5
−10
−15
0
10
20
30
40
50
60

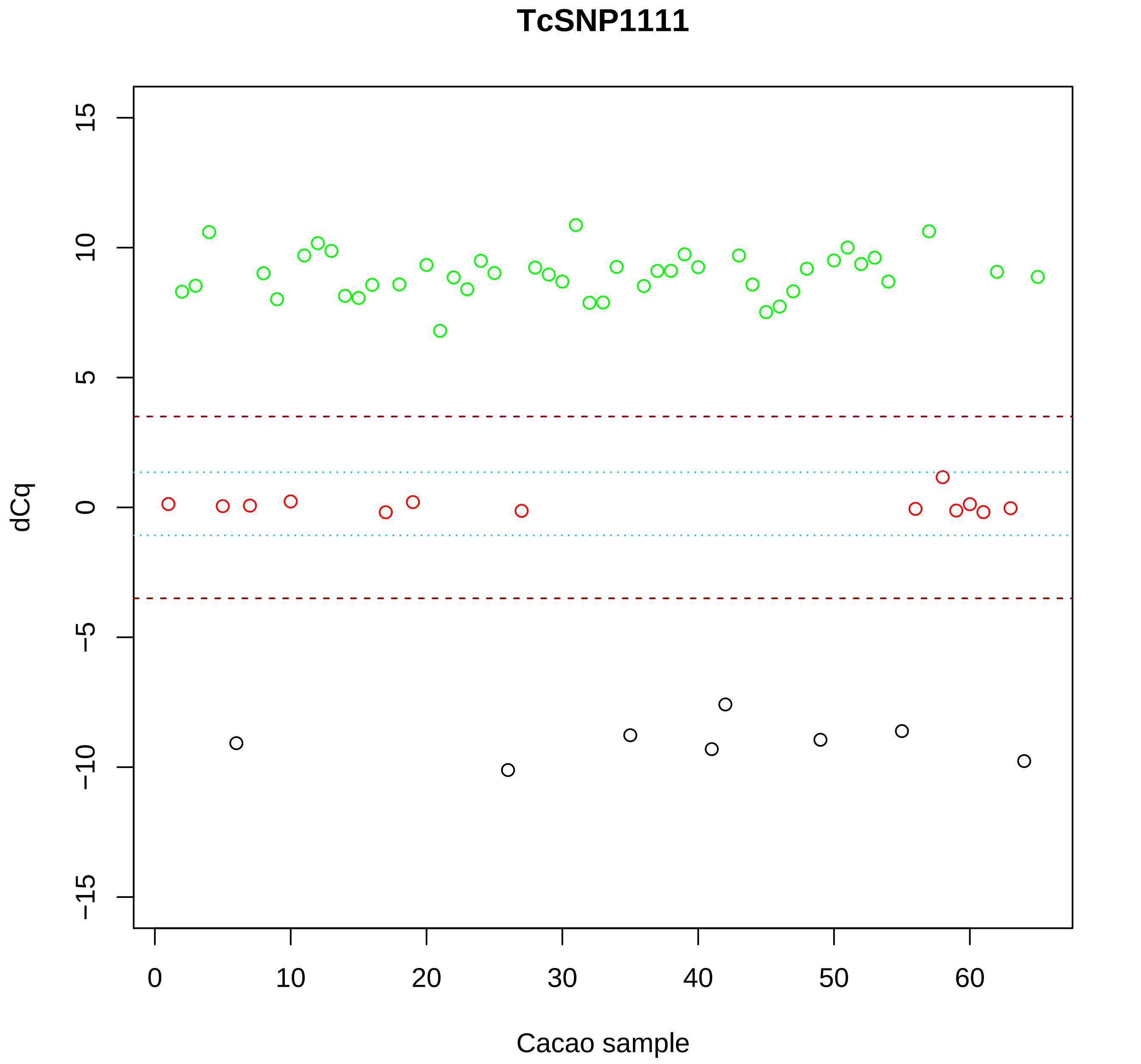

TcSNP1111
dCq
Cacao sample
15
10
5
0
−5
−10
−15
0
10
20
30
40
50
60

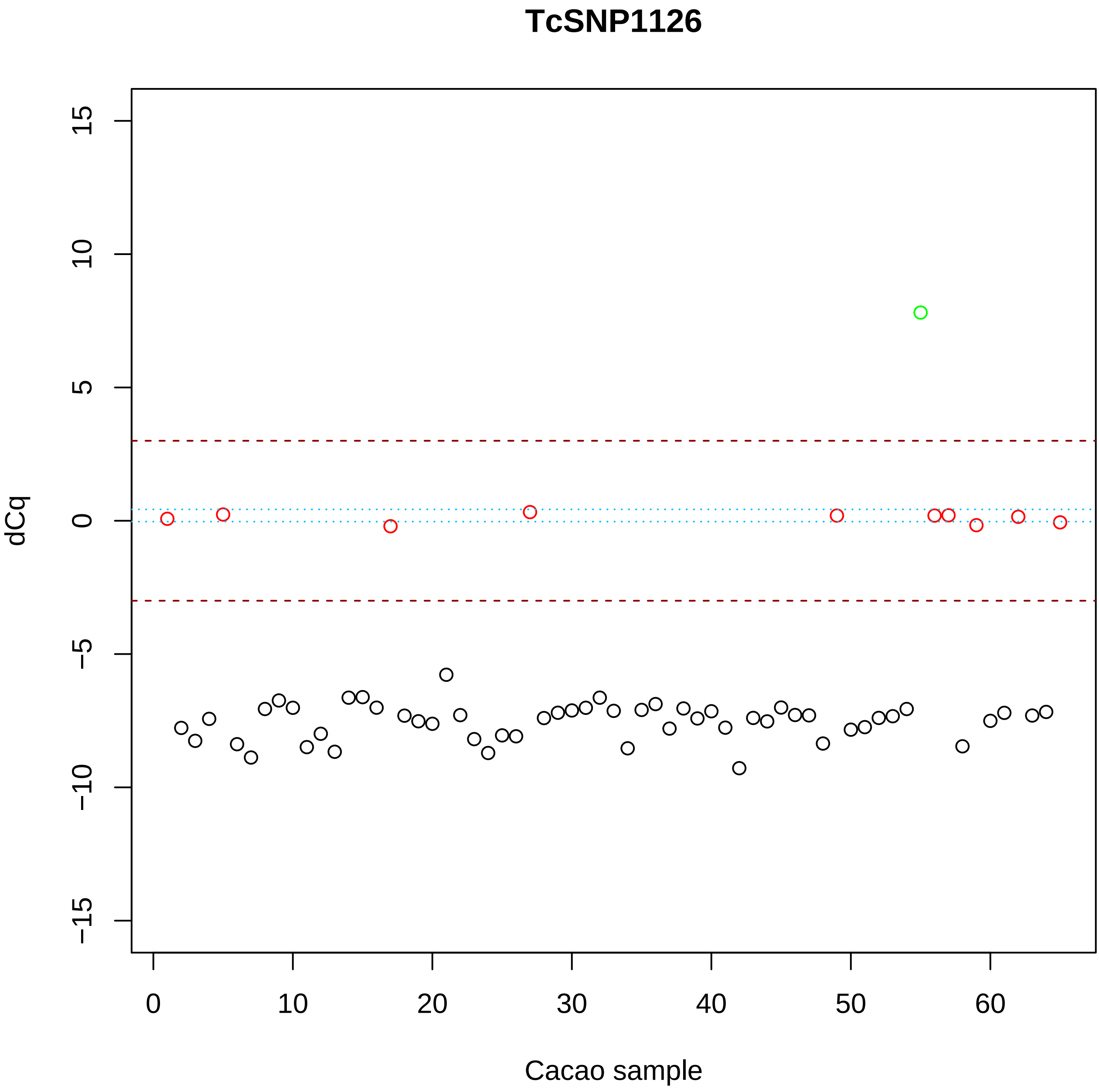
TcSNP1126
dCq
Cacao sample
15
10
5
0
−5
−10
−15
0
10
20
30
40
50
60

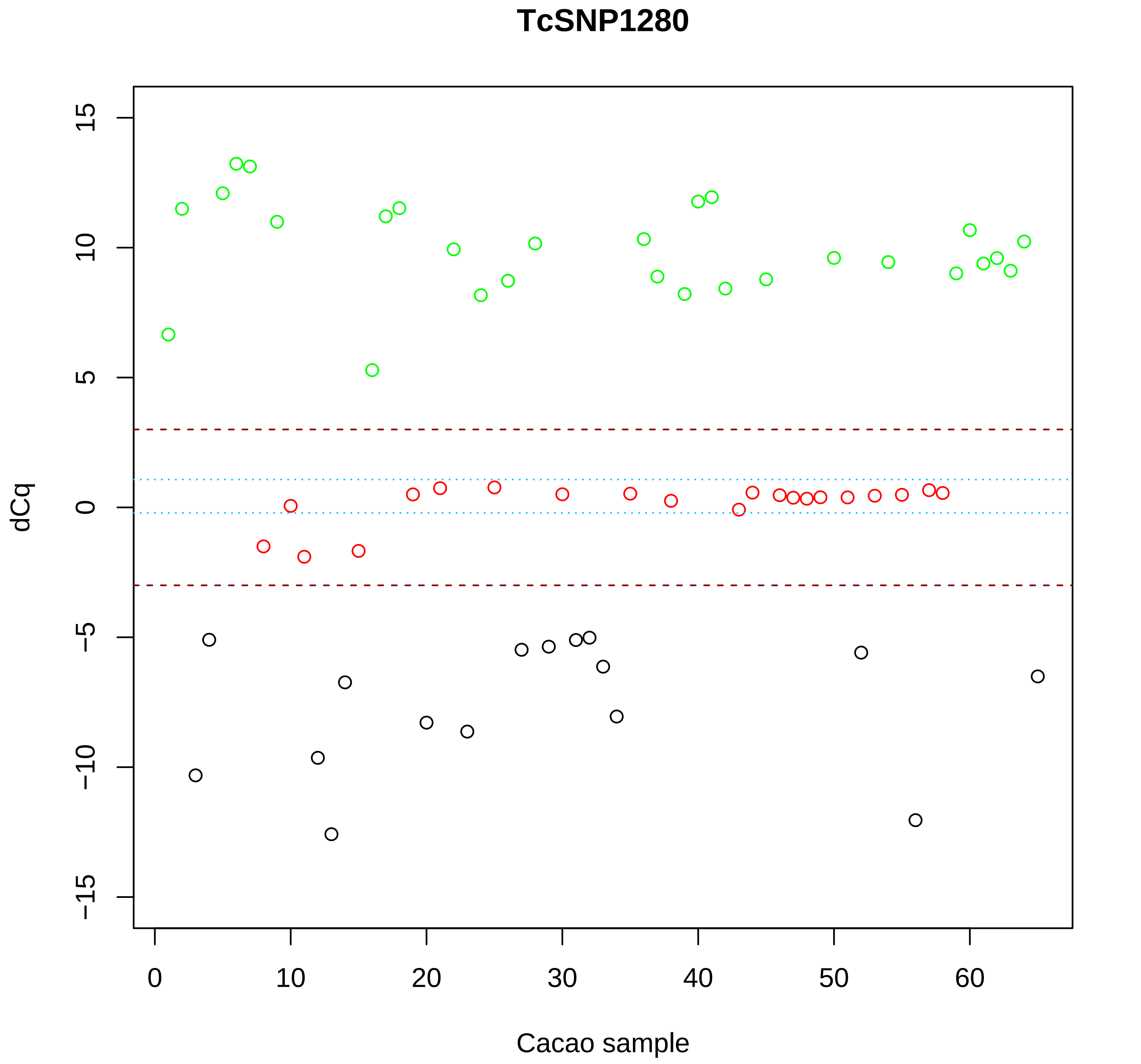

TcSNP1280
15
10
5
0
−5
−10
−15
dCq
0
10
20
30
40
50
60
Cacao sample

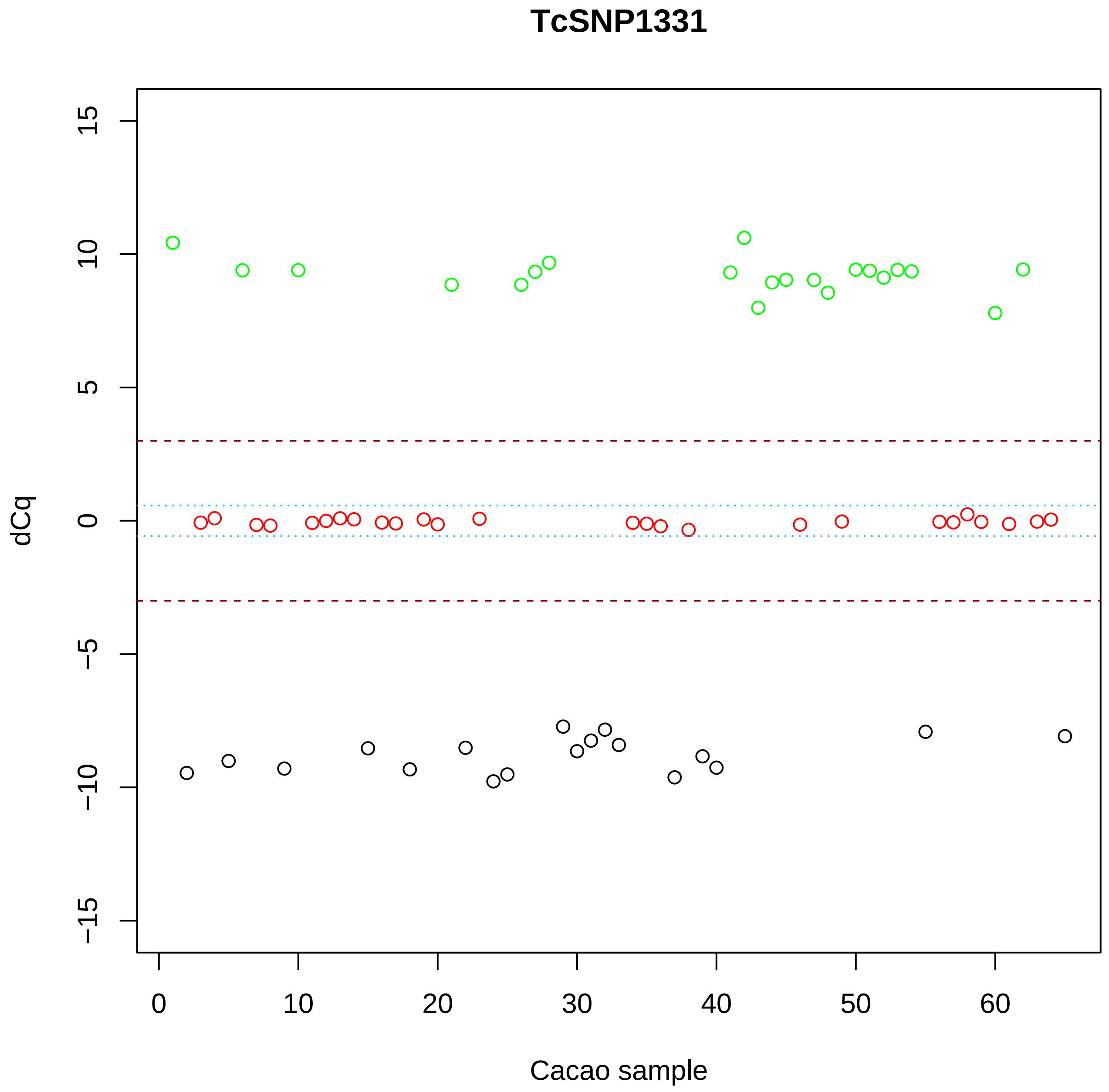
TcSNP1331
dCq
Cacao sample
15
10
5
0
−5
−10
−15
0
10
20
30
40
50
60

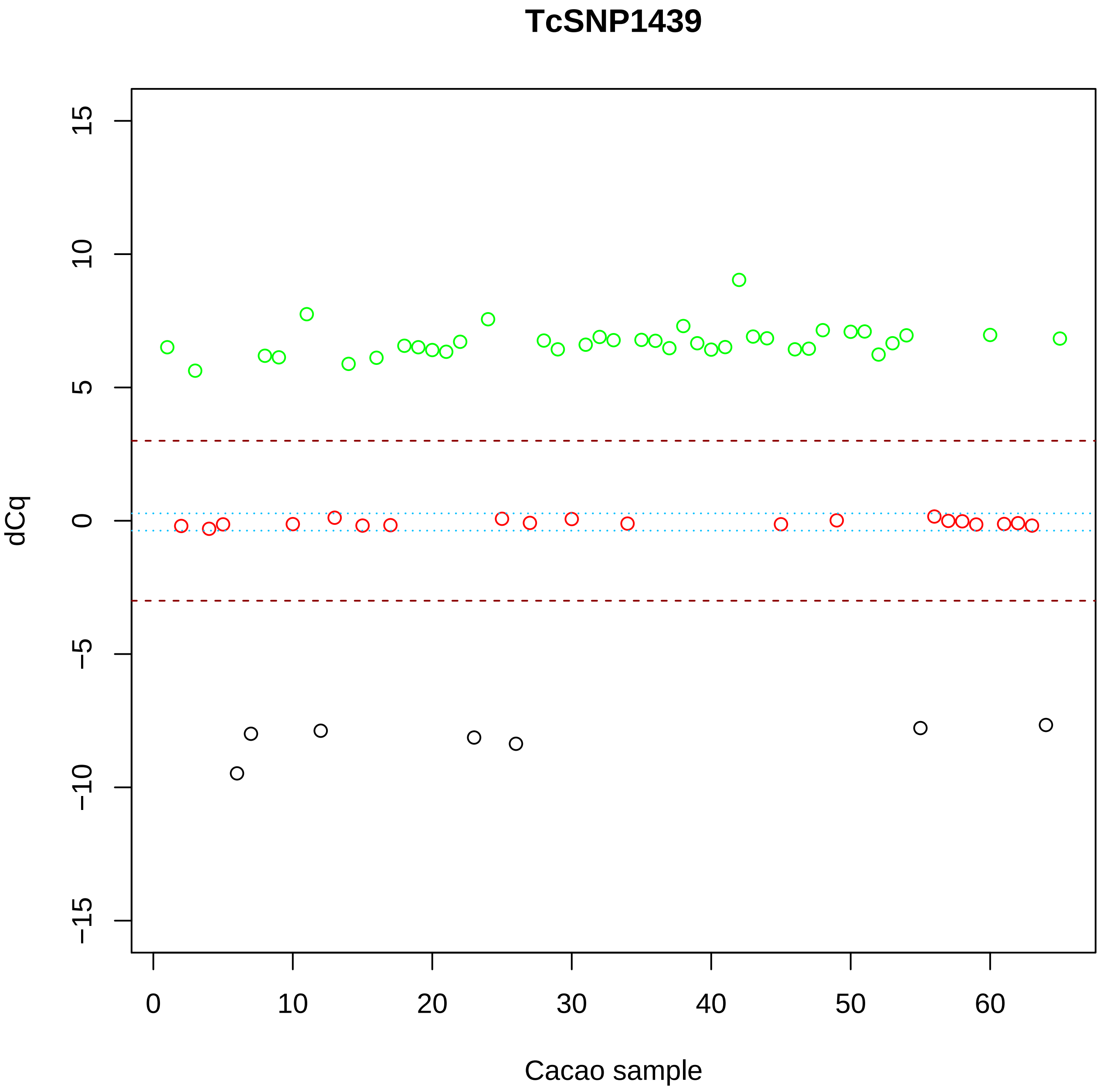
TcSNP1439
dCq
Cacao sample
15
10
5
0
−5
−10
−15
0
10
20
30
40
50
60

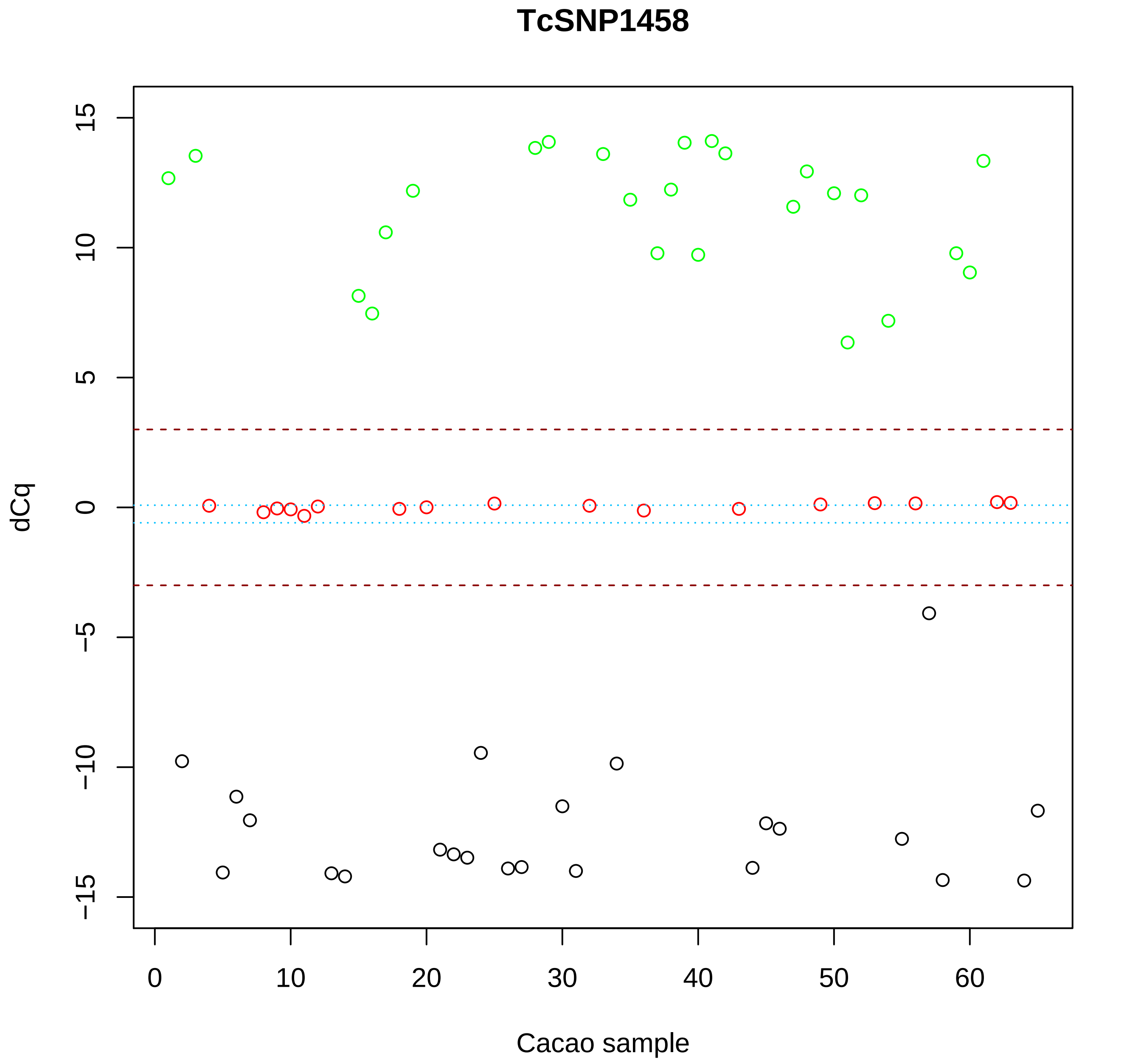
TcSNP1458
dCq
Cacao sample
15
10
5
0
−5
−10
−15
0
10
20
30
40
50
60

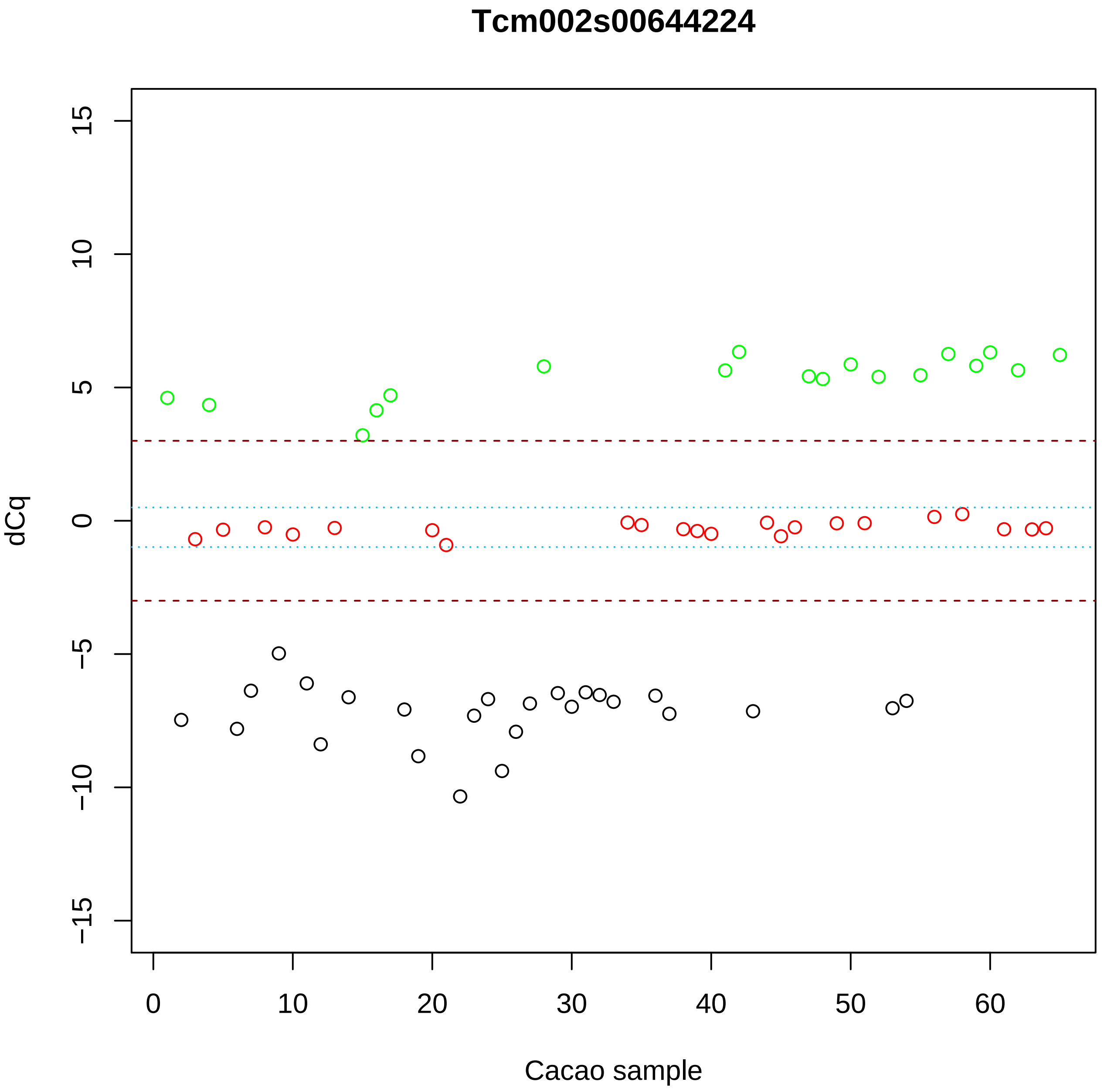

Tcm002s00644224
dCq
15
10
5
0
−5
−10
−15
0
10
20
30
40
50
60
Cacao sample

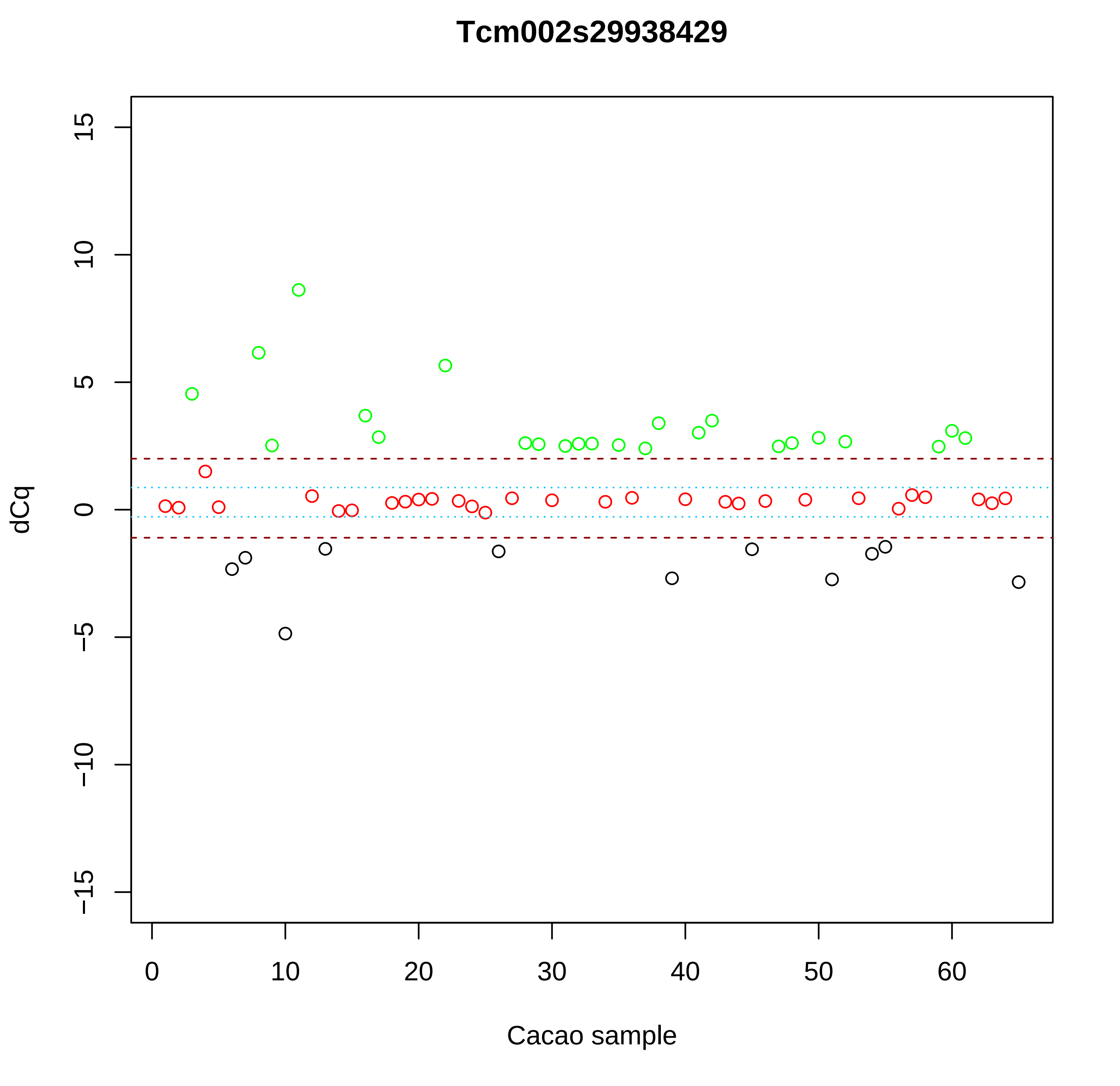

Tcm002s29938429
dCq
Cacao sample
15
10
5
0
−5
−10
−15
0
10
20
30
40
50
60

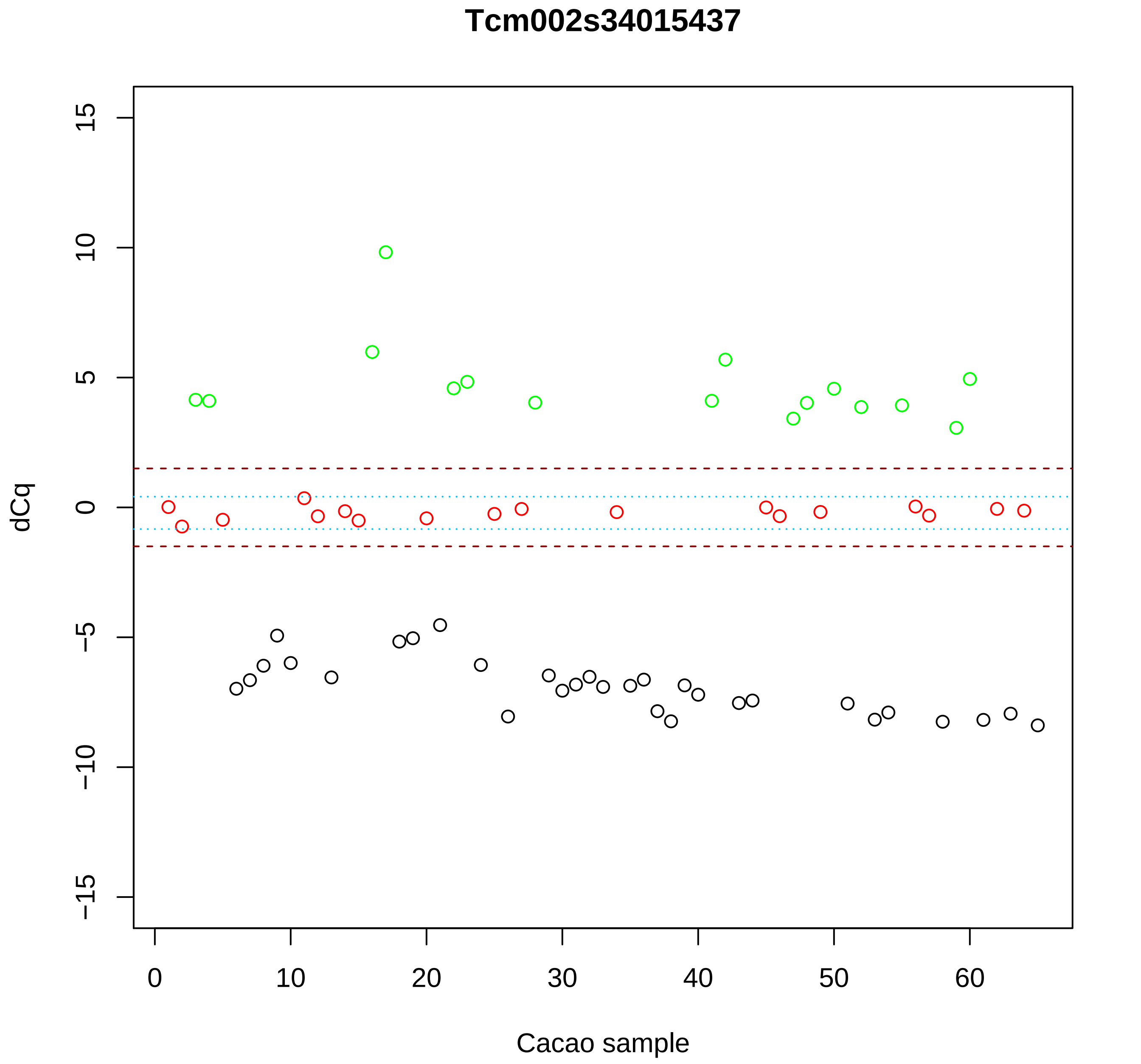
Tcm002s34015437
dCq
Cacao sample
15
10
5
0
−5
−10
−15
0
10
20
30
40
50
60

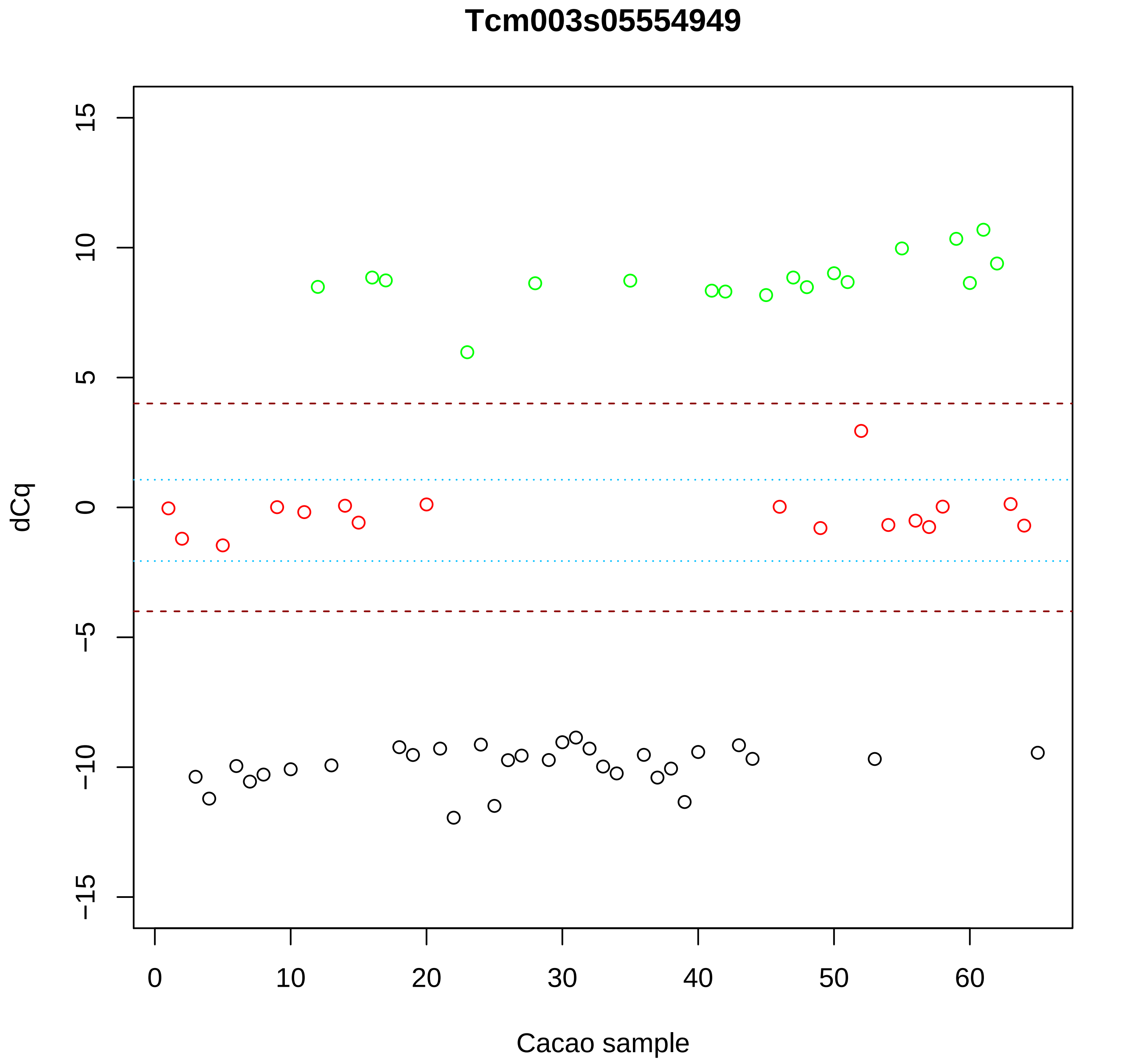

Tcm003s05554949
dCq
Cacao sample
15
10
5
0
−5
−10
−15
0
10
20
30
40
50
60

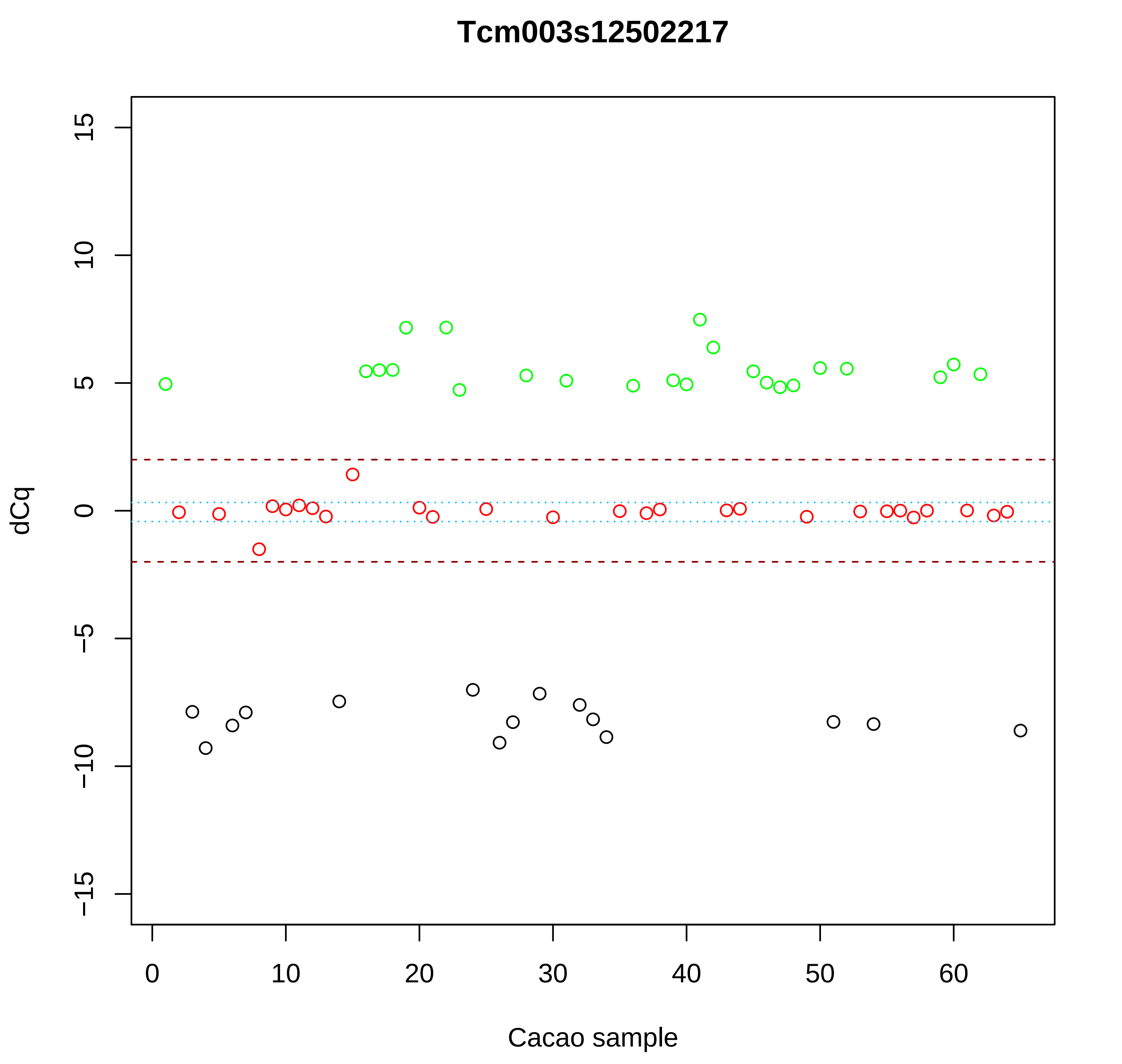
Tcm003s12502217
dCq
Cacao sample
15
10
5
0
−5
−10
−15
0
10
20
30
40
50
60

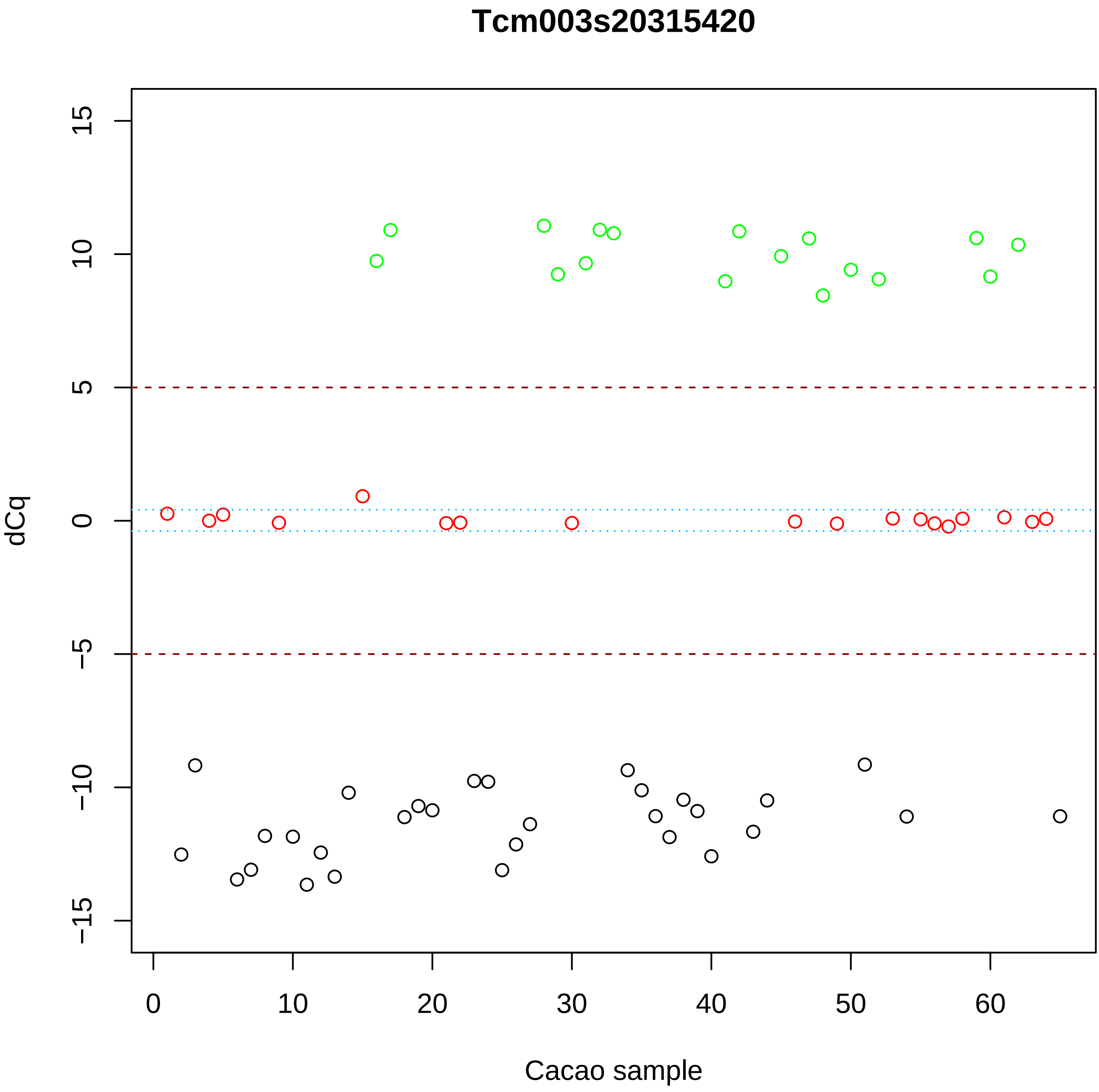

Tcm003s20315420
dCq
15
10
5
0
−5
−10
−15
0
10
20
30
40
50
60
Cacao sample

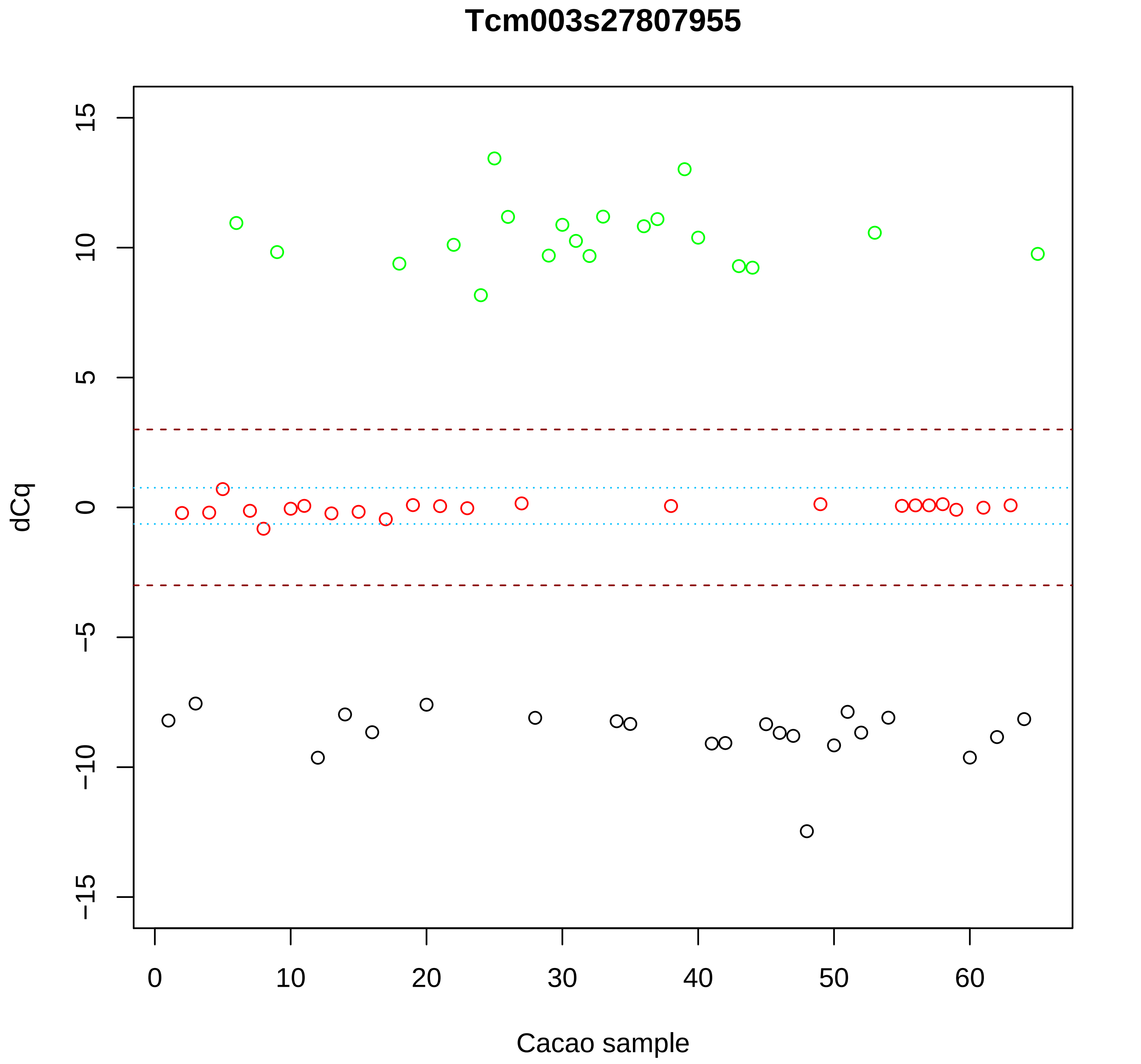

Tcm003s27807955
dCq
Cacao sample
15
10
5
0
−5
−10
−15
0
10
20
30
40
50
60

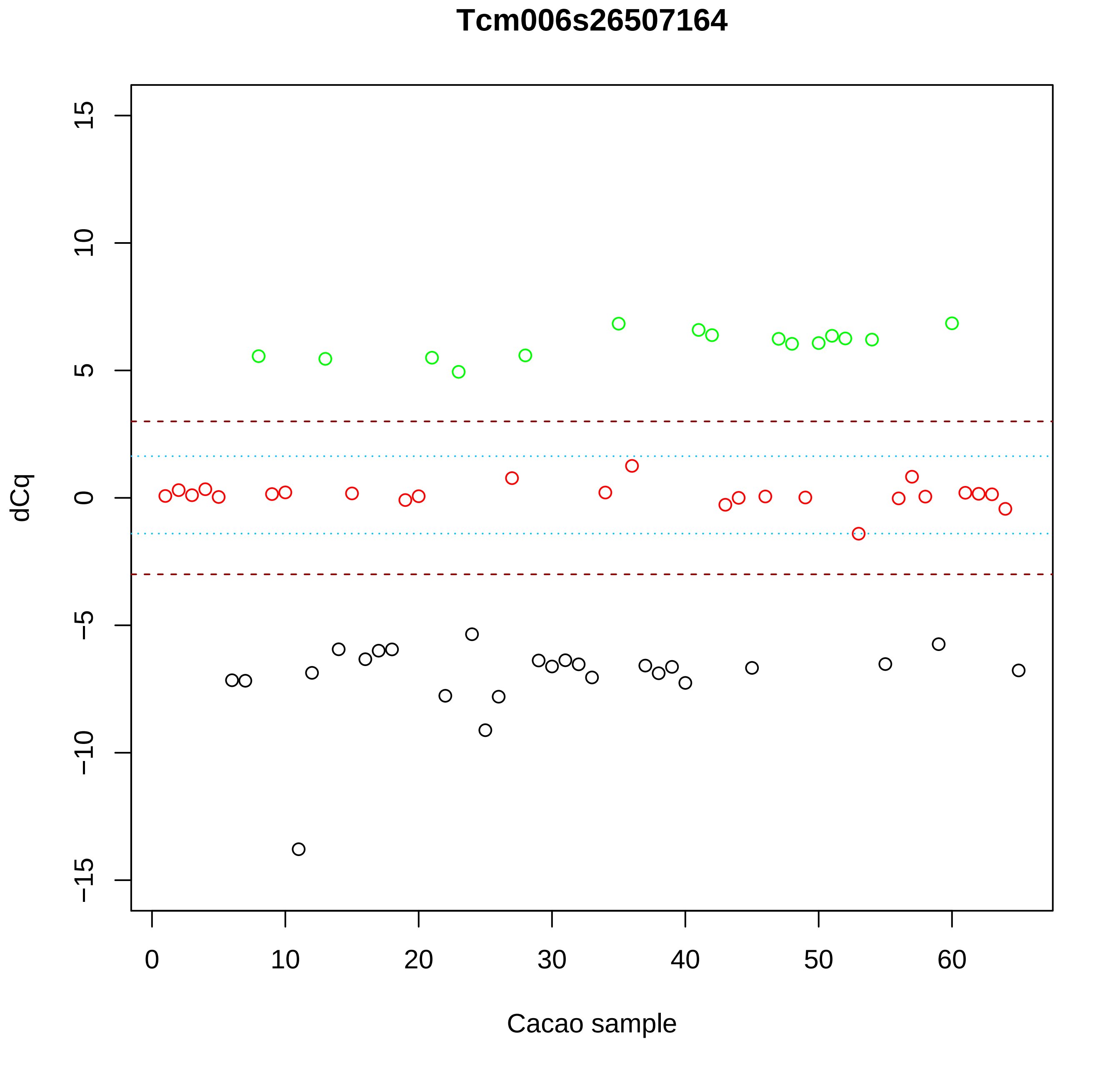
Tcm006s26507164
dCq
15
10
5
0
−5
−10
−15
0
10
20
30
40
50
60
Cacao sample

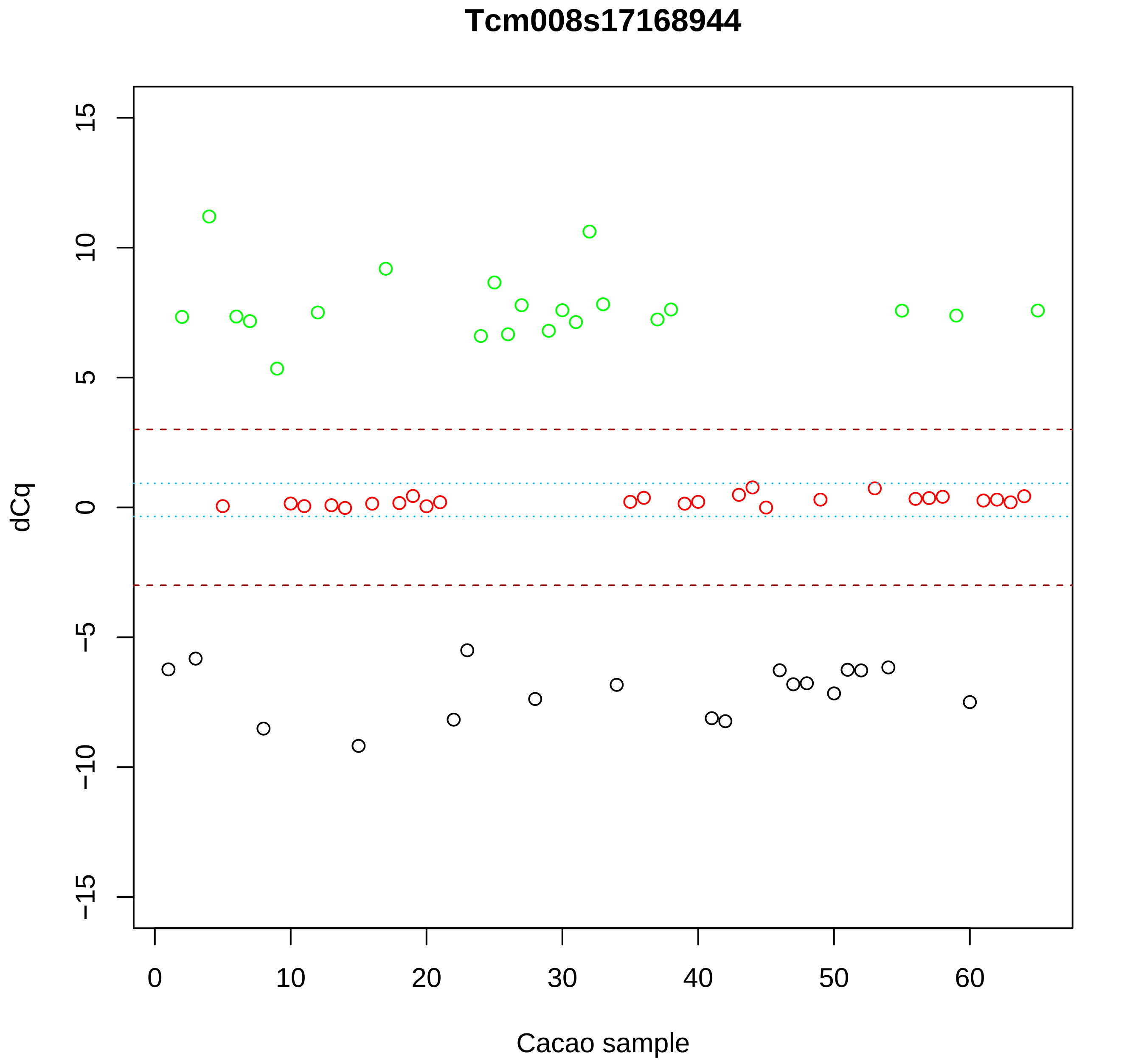

Tcm008s17168944
dCq
Cacao sample
15
10
5
0
−5
−10
−15
0
10
20
30
40
50
60

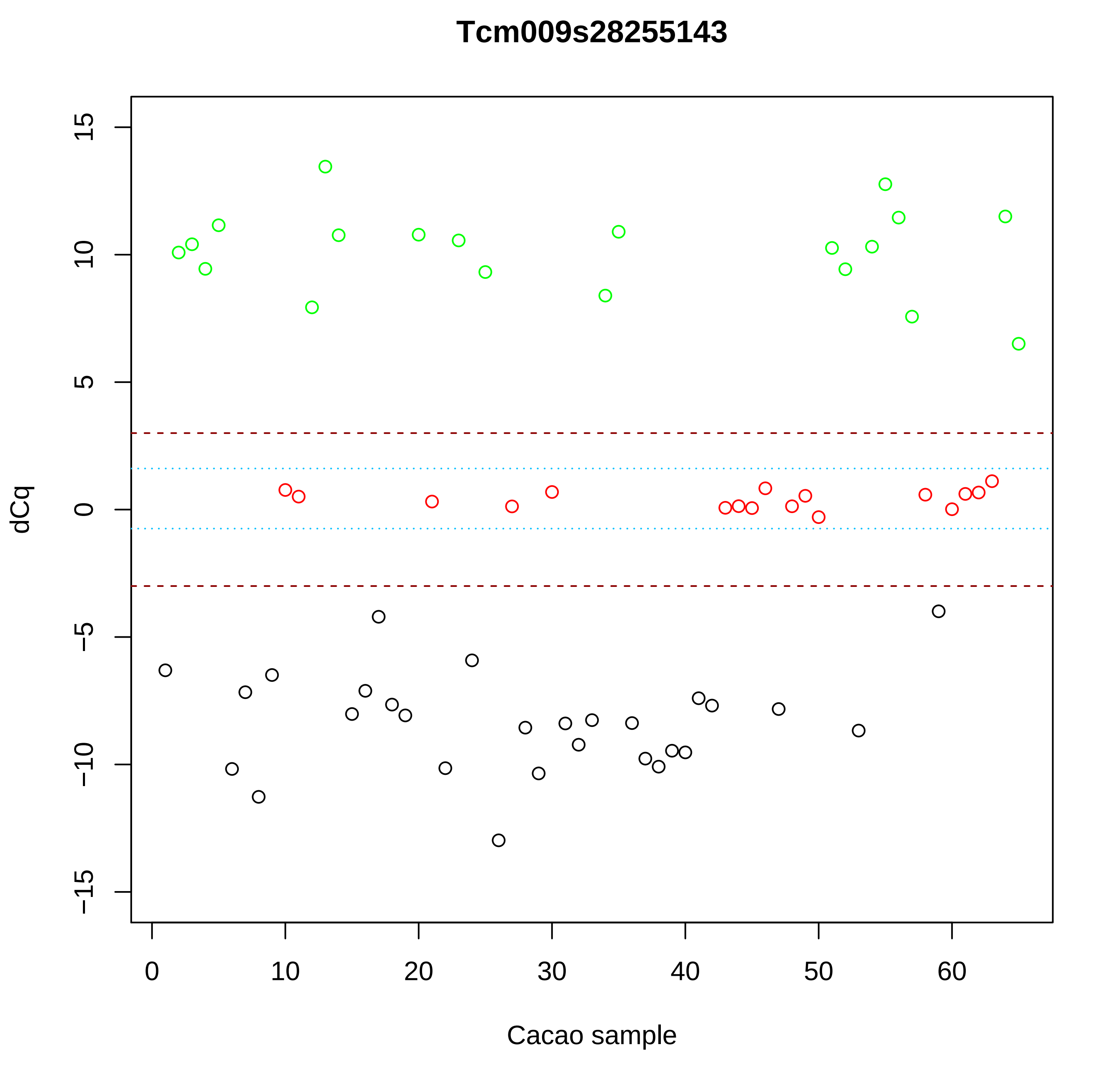
Tcm009s28255143
dCq
Cacao sample
15
10
5
0
−5
−10
−15
0
10
20
30
40
50
60

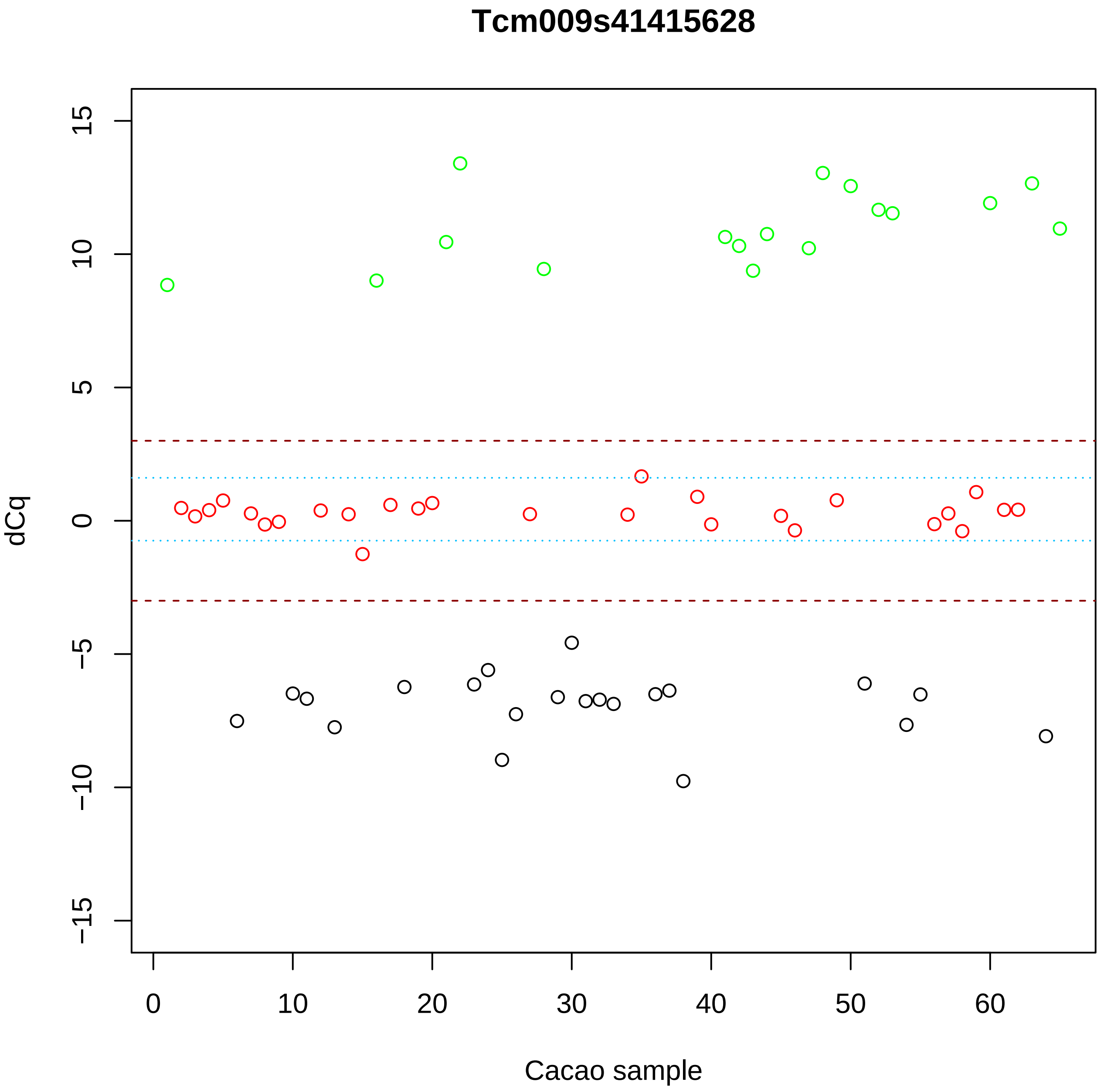

Tcm009s41415628
dCq
15
10
5
0
−5
−10
−15
0
10
20
30
40
50
60
Cacao sample
